# Significant Progress in the Study of African Freshwater Snails Over the Past 260 Years

**DOI:** 10.1002/ece3.71031

**Published:** 2025-02-20

**Authors:** Matabaro Ziganira, Colleen T. Downs

**Affiliations:** ^1^ Centre for Functional Biodiversity, School of Life Sciences University of KwaZulu‐Natal Pietermaritzburg South Africa; ^2^ KwaZulu‐Natal Museum Pietermaritzburg South Africa

**Keywords:** Africa, freshwater ecosystems, literature review, malacology, snails

## Abstract

Globally, freshwater ecosystems are threatened. Research progress concerning African freshwater snails was reviewed using a systematic review process. Since 1757, the number of publications produced has increased, particularly in the last decade. In the first 50 years (1757–1800), 0.1% of publications on freshwater snails in Africa were conducted, followed by 0% (1801–1850), 3.3% (1851–1900), 3.5% (1901–1950) and 48.7% (1951–2000). The last 23 years (2001–2024) exhibited a large increase (44.3%) in publications of the total conducted. Studies on freshwater snails varied in number across the 10 major African water basins, with the majority of studies in the Nile (21.7%), followed by the Congo Basin (17.6%) and Niger (12.4%). The Orange Basin and Lake Tanganyika also received a high number of studies (10.9%) and (7.2%), respectively. Most freshwater snail study objectives related to conservation and taxonomy (70%), followed by disease vector (20.5%), with genetics/genomic/DNA barcoding/eDNA receiving significant focus as well (5.2%). Studies focusing on geology and palaeontology (2.5%), followed by climate change (1.5%) and machine learning (0.4%). The modern phase in the study of African freshwater snails came around the early 20th century with the discovery of *Bulinus truncatus* and *Biomphalaria alexandrina* as intermediate hosts for the parasites causing human schistosomiasis. African freshwater malacology has since then benefited from African and overseas malacologists based at universities and medical laboratories across Africa and overseas. In addition to taxonomic studies, there was a steady rise in contributions relating to ecology, disease vectors, palaeontology and genetics. These contributed knowledge on local endemism and speciation, invasive species, species origins and distribution across African water basins, as well as the spread of infectious diseases and impacts of climate change. In the last decade, there have been shifts in methods with the application of DNA barcoding, genomics, environmental DNA and, most recently, machine learning approaches.

## Graphical abstract

Globally, freshwater ecosystems are threatened, and studies estimate rapid degradation compared with terrestrial and marine ecosystems. Like most other tropical regions, Africa still has far to go regarding discovery and documentation. Using a systematic review process, we evaluated freshwater African snail studies. We assessed freshwater snail publications from the earliest to the present to quantify trends regarding snail research and as vectors of diseases, ecology, genetics, taxonomy, pest control and invasive alien species in African countries.

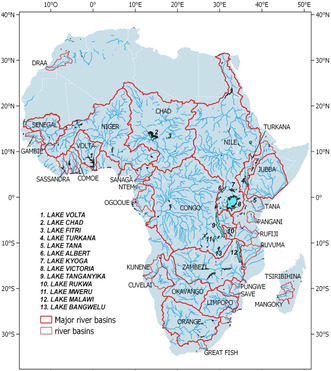

## 1 Introduction

Africa is the second largest continent in the world, both in size (~30 million km^2^) and population (1.4 billion inhabitants as of 2022) (Guttal 2021; United Nations 2019). The African continent hosts some of the largest freshwater ecosystems in the world, including the Nile, the longest river in the world, and the Congo River, the world's second‐largest basin both in terms of drainage area and discharge to the Atlantic Ocean (Dai et al. 2009; Laraque et al. 2020; Papa et al. 2023). In terms of area and volume, Africa also has three of the ten largest freshwater lakes on Earth, including Lake Victoria, Lake Tanganyika and Lake Malawi (Hernegger et al. 2021).

**FIGURE 1:**
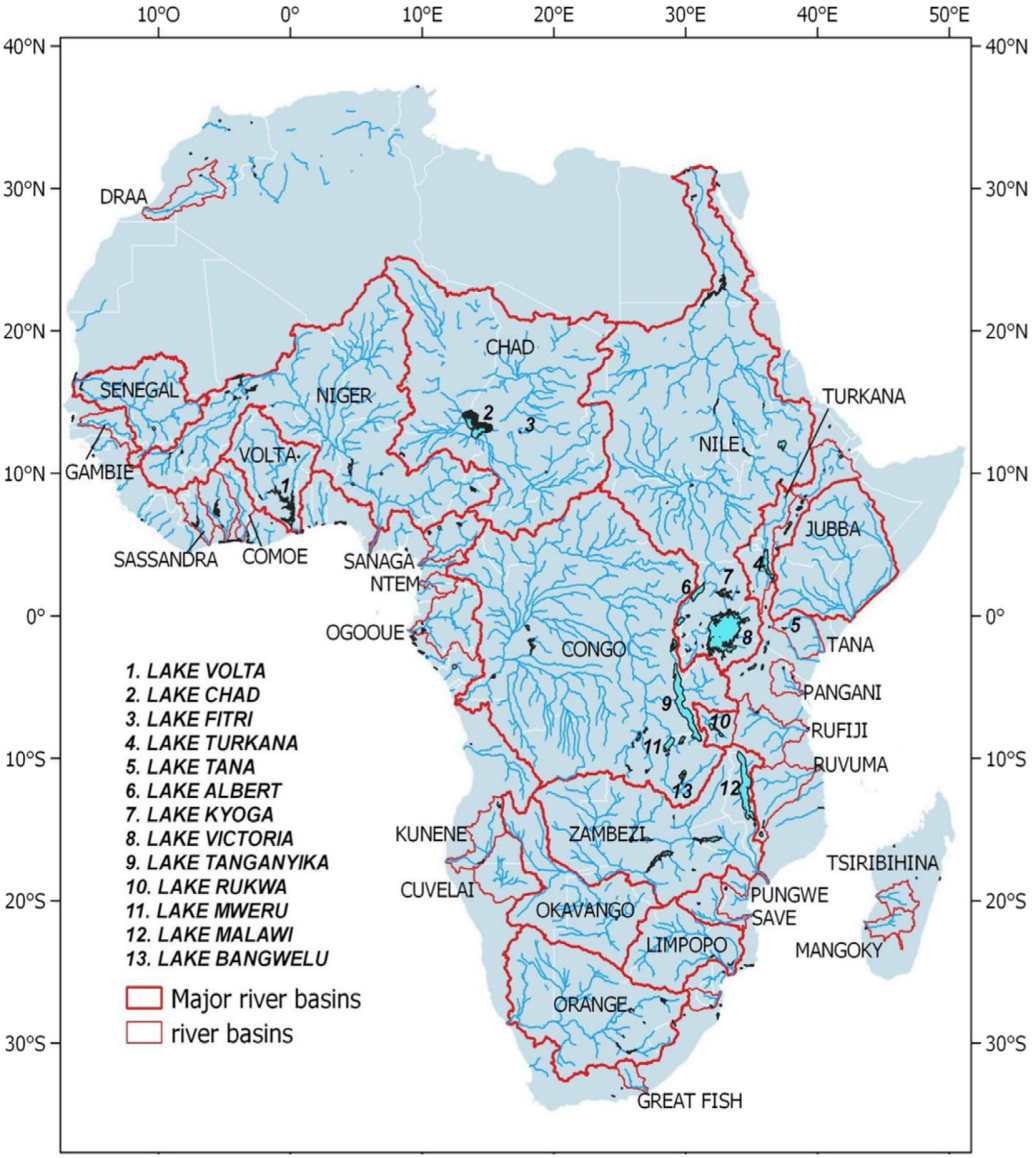
River basins and lakes in Africa (adapted from Papa et al. 2023).

The rivers and other surface water bodies in sub‐Saharan Africa are often shared by two or more countries (Table 1). Drainage basins with more than five basin countries are Chad, Volta, Zambezi, Niger, Congo and the Nile, and are of global significance for biodiversity and the carbon and nutrient cycles (Hastie et al. 2021; Lunt et al. 2019; Simaika et al. 2021; Figure 1). The African landscapes are also made up of smaller water systems, such as streams, reservoirs, ponds and tanks, which provide drinking water and agricultural resources to rural populations (Gardelle et al. 2010).

**TABLE 1:** The 10 largest surface water bodies in sub‐Saharan Africa shared by two or more countries (IPCC 2001).

Basin name	Number of countries	Basin area (1000 km^2^)	Basin countries
Congo	9	3720	Congo, Tanzania, Cameroon, Burundi, Rwanda, Zambia, DRC,^a^ Central African Republic, Angola
Nile	11	3031	Sudan, South Sudan, Ethiopia, Egypt, Uganda, Tanzania, Kenya, Rwanda, Burundi, DRC, Eritrea
Niger	9	2200	Mali, Nigeria, Niger, Guinea, Cameroon, Burkina Faso, Benin, Cote d'Ivoire, Chad
Lake Chad	6	1910	Chad, Niger, Central African Republic, Nigeria, Sudan, Cameroon
Zambezi	8	1385	Zambia, Angola, Zimbabwe, Mozambique, Malawi, Botswana, Tanzania, Namibia
Orange	4	950	South Africa, Namibia, Botswana, Lesotho
Okavango	4	529	Botswana, Angola, Namibia, Zimbabwe
Limpopo	4	385	South Africa, Botswana, Mozambique, Zimbabwe
Volta	6	379	Burkina Faso, Chana, Togo, Cote d'Ivory, Benin, Mali
Senegal	4	353	Mali, Mauritania, Senegal, Guinea

^a^ Democratic Republic of the Congo.

The Afrotropical region possesses a wide range of ecosystems with varying degrees of species diversity within them (Davis et al. 1994; Klopper et al. 2002; Lindsey et al. 2022; Stuart et al. 1990). Biodiversity is not evenly distributed on Earth, and the Afrotropical region is no exception. Species distribution within ecosystems in the Afrotropical region is influenced by climate, soil type and topography and has been recorded higher in lowland equatorial rainforest (Aquilas et al. 2022; Stuart et al. 1990) than in other ecoregions/zones. In Africa, topography and rainfall affect species richness and abundance, with more species recorded in regions with a wide variety of ecosystems (Papa et al. 2023; Stuart et al. 1990; Tumwebaze et al. 2022; Figure 1). The Democratic Republic of the Congo (DRC) for instance, is probably regarded as the richest nation in Africa for biodiversity because of its extensive lowland rainforests, northern and southern savannahs, large areas of wetlands and lakes and mountains (Aquilas et al. 2022; Stuart et al. 1990). While South Africa is considered the second richest country for biodiversity mainly because of its uniquely rich flora of the Cape regions, other African countries with internationally important biodiversity include Cameroon, Kenya, Nigeria, Tanzania, Uganda and Madagascar (Aquilas et al. 2022; Griffiths et al. 2010; Skowno et al. 2019; Stuart et al. 1990).

Globally, freshwater ecosystems are under threat (Vörösmarty et al. 2010), and studies estimate a rapid degradation compared with terrestrial and marine ecosystems (Darwall et al. 2011; Dudgeon et al. 2006; Reid et al. 2019; van Klink et al. 2020). Africa is well known for its renowned freshwater ecosystems (Masese et al. 2023; Figure 1). While many rivers in Africa are being dammed for hydropower and water supply for agriculture, forestry and urban activities (McClain 2013; O'Brien et al. 2021), lakes are being used for transportation, fishing, water supply and cultural activities. Unfortunately, water withdrawal, pollution, invasive species, overfishing and biomass harvesting around wetlands with human settlements pose threats to these ecosystems (Dalu and Wasserman 2022).

Studies have shown that freshwater ecosystems such as rivers, lakes and wetlands provide essential ecosystem services (food and water) that support livelihoods (Danaher et al. 2022; Kafumbata et al. 2014; McClain 2013; Sayer et al. 2018). Freshwater ecosystems also play an important role in preventing floods or droughts and are crucial to human existence by transporting nutrients and providing health as well as recreational benefits (Albert et al. 2021; Dudgeon et al. 2006; Vörösmarty et al. 2010). Molluscs (bivalves and gastropods) are found in a wide range of freshwater ecosystems (Seddon et al. 2011), contribute to bioturbation and biofiltration in freshwater lotic and lentic environments (Bogan 2008; Covich et al. 1999; Palmer et al. 1997). Also, molluscs play an important role in stabilising the food web by feeding on algae, bacteria, zooplankton, detritus, dissolved organic matter, diatoms and plants (Alzurfi et al. 2019), and some provide a source of protein and income to local communities (Köhler et al. 2012; Shivambu et al. 2020). Besides the ecosystem services, freshwater molluscs are constantly used as proxies in biodiversity research because of their wide range of distribution in freshwater habitats, varied life history strategies and complex ecological interactions (Seddon et al. 2011); and important vectors of parasitic diseases (Chimbari et al. 1997, 2020; Chimbari 2012; Kapour, Wambui, Madinga, et al. 2024a; Kapour, Wambui, Schols, et al. 2024b; Muzarabani et al. 2024).

It is imperative to gain an understanding of mollusc species diversity and distribution, and to prevent snail‐transmitted parasitic diseases in communities dependent on freshwater resources (Chingwena et al. 2004; EI‐Gindy 1957; Genner et al. 2007; Krings et al. 2021). In terms of molluscs, Lake Tanganyika belongs to the East African Great Lakes and is well known for harbouring a high proportion of endemic and morphologically distinct genera, and a natural laboratory to study speciation (Krings et al. 2021). Compared with Europe and North America, freshwater molluscs of Africa are less diverse, with an estimated 560 species, while North America and Europe have an estimated 880 species and 780 species, respectively (Bogan 2008; Seddon et al. 2011). It is estimated that freshwater molluscs in Africa are approximately represented by 75% gastropods and 25% bivalves (Seddon et al. 2011). Species diversity of freshwater molluscs in Africa varies with subregion, with the highest diversity recorded in the East African Great Lakes (Lake Victoria, Lake Tanganyika and Lake Malawi), the Upper Congo catchment around Lake Mweru, the river rapid regions of western Africa and the Lower Congo Rapids (Seddon et al. 2011). The regions of North‐western Africa (the Maghreb), the East African coastal rivers, the Lower Nile River, the Lake Chad basin and the Malebo Pool in the Congo Basin are also considered important for freshwater mollusc biodiversity (Seddon et al. 2011).

However, like most other tropical regions, freshwater snail diversity of Africa has drawn interest from both foreign and local scientists and collectors. Scientific study of African freshwater snails began nearly 260 years ago, with the arrival of European traders and explorers (Brown 1994). Presently, significant advances have been made in all the main fields of research: conservation, taxonomy, genetics, climate change, ecology, public health and more. Despite these, there are still gaps that need to be addressed, including (1) lack of snail life history studies with regard to freshwater basin connectivity on the continent; and (2) lack of categorisation of fields of study in publication records over the past four centuries. It could be suggested that research on freshwater snails is underrepresented in some African regions and has been largely sidelined compared with terrestrial and marine molluscs. To date, little has been done to document the significant progress in the study of African freshwater snails over the past 260 years. The aim of this literature review was to assess the significant research progress in the study of African freshwater snail biology over the past four centuries. We believe that understanding the historical and present research efforts on freshwater snails across the continent is fundamental for addressing various challenges associated with biodiversity conservation, sustainable development and climate change.

## 2 Methods

### 2.1 Data Collection

We conducted a literature search on research on freshwater snails in rivers, lakes, wetlands and ponds across Africa and its islands. We compiled data from international peer‐reviewed published journals and conference proceedings of freshwater snail research across all African countries. Because of the geo‐political reasons on the continent, not all literature was in English. Because of this, literature in French, Portuguese and Spanish were included (d'Ailly 1896; Ancey 1906; Azevedo et al. 1961; Bacci 1951; Baluku et al. 1989; Barré et al. 1982; Binder 1957, 1968; Bourguignat 1885, 1886, 1889, 1890; Casaca and de Lima Carvalho 1955; Dupouy et al. 1980; Germain 1908, 1920, 1923, 1925; Julvez et al. 1990; Kristensen 1985; Leloup 1953; Lévêque 1967; Morais 1975; Sellin et al. 1980). Editorials, letters, comments and books were also included. Article titles were found using the Biodiversity Heritage Library, Google Scholar, Scopus and Web of Science online search engine databases using the search phrase ‘Freshwater snails in Africa’ with the following keyword (*) basin name, ancient and new name of lakes, rivers, wetlands, snails, gastropod, bivalve and individual country in Africa (new and ancient names). The search string used was not limited to publication date, ensuring all freshwater snail publications conducted in Africa were included, with the first being published in 1757 (Adanson 1757). Publications were incorporated in the review if they represented malacology‐based research focused on African water basins, including rivers, lakes, wetlands and ponds. We recorded the results for each publication in a Microsoft Excel spreadsheet, and data were refined/sorted by year of publication.

### 2.2 Analyses of Research Focus on Freshwater Snails

**FIGURE 2:**
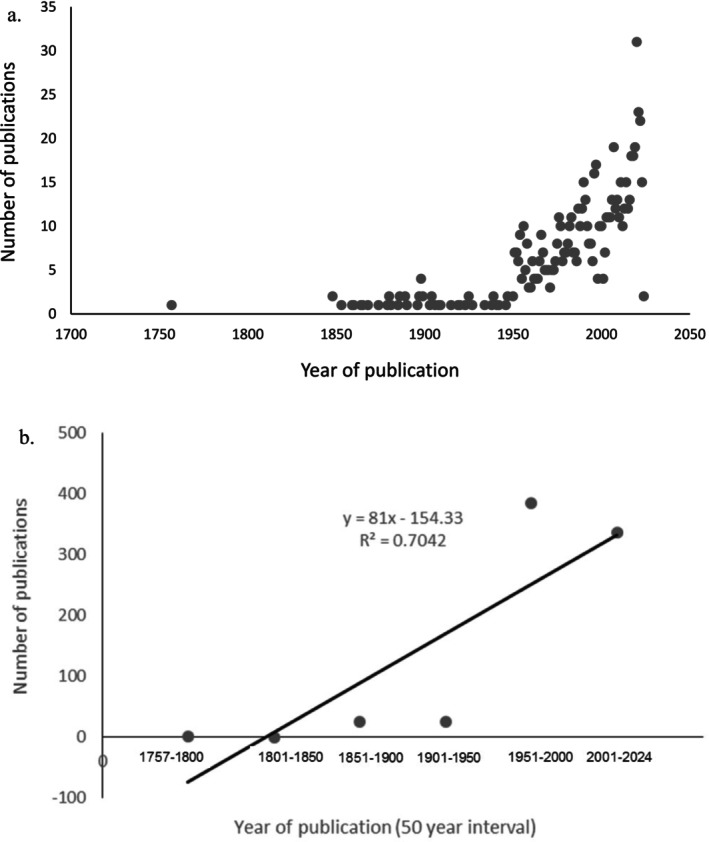
Number of published studies on freshwater snails conducted in Africa between 1757 and 2024. Data are grouped at 50‐year intervals in both a and b. The last 23 years (2001–2024) as shown in b, exhibited a large increase in publications, with 44.3% of publications on freshwater snails of the total conducted in Africa during this time.

**FIGURE 3:**
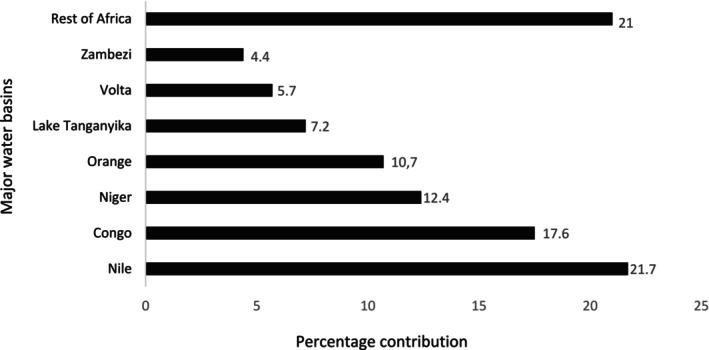
Percentage contribution of African major water basins showing published studies on freshwater snails conducted in Africa between 1757 and 2024 across African water basins.

To investigate the various interdisciplinary fields of study, we recorded and summarised a range of variables. These included the year of publication, the water basin or country in which the study was conducted and the fields of study. We used the year of publication of each study to assess temporal trends in research (Figure 2). The year of publication dates ranged from 1757 (18th century) to 2024 (21st century). Research publications were assessed in the 10 largest surface water basins in sub‐Saharan Africa and their associated countries (Figure 3; Table 1).

**FIGURE 4:**
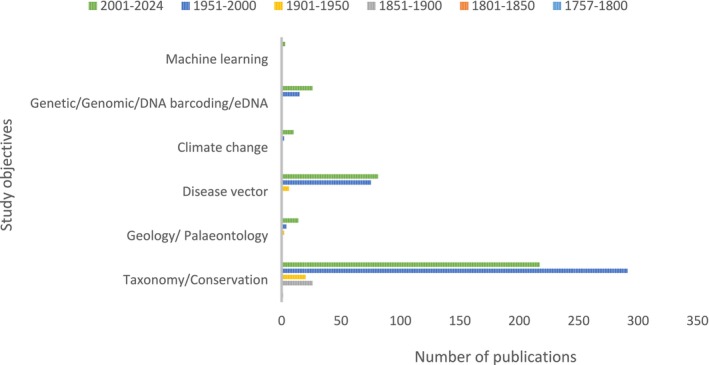
Total number of freshwater snail study objectives recorded across African water basins between 1757 and 2024.

To better understand knowledge gaps, we assessed each publication against six primary fields of study. Studies were grouped into a maximum of six primary fields of study because some publications covered multiple aspects of biology, conservation or ecology. The six primary fields of study identified were as follows: 1. taxonomy and conservation (biodiversity and ecology‐related studies); 2. geology and palaeontology (paleoecology and palaeogeography); 3. disease vector studies on parasitic infections such as schistosomiasis (bilharzia), *Hepatic distomatosis* and *Paramphistomum daubneyi*; 4. climate change; 5. genetic/genomic and DNA barcoding/eDNA; and 6. machine learning approaches (Figure 4).

### 2.3 Statistical Analyses

We collated all these data and used descriptive statistics to show trends. We used regression analysis and percentages generated using Microsoft Excel 2013 to show trends.

## 3 Results and Discussion

### 3.1 Publications

Our literature research generated 791 publications from books, editorials and peer‐reviewed journals (Table S1; Ziganira and Downs 2024). Since 1757, the number of subsequent publications produced has increased rapidly, particularly in the last decade (Figure 2, Table 2). In the first 50 years (1757–1800), 0.1% of publications on freshwater snails in Africa were conducted, followed by 0% (1801–1850), 3.3% (1851–1900), 3.5% (1901–1950) and 48.8% (1951–2000). The last 23 years (2001–2024) exhibited a large increase in publications, with 44.3% of publications on freshwater snails of the total conducted in Africa during this time (Figure 2, Table 2).

**TABLE 2:** Total number of freshwater snail studies conducted in Africa for each field of study between 1757 and 2024, and also presented as a percentage of the total number of studies.

Year interval	Total number of studies conducted for each field of study	Percentage of total studies (%)
1757–1800	1	0.1
1801–1850	0	0
1851–1900	26	3.3
1901–1950	28	3.5
1951–2000	385	48.7
2001–2024	351	44.3

### 3.2 Distribution of Research on Freshwater Snails in Africa

Studies on freshwater snails varied in number across major African water basins between 1757 and 2024, with several water basins displaying considerably higher numbers of freshwater snail publications than the rest. Of the 54 African countries, studies on freshwater snails were conducted in seven major water basins (625/791, 79%), with studies (21%) occurring across multiple water basins and countries (Figure 3; Table S2; Ziganira and Downs 2024). To determine the percentage contribution made by each African water basin, we treated each basin and associated countries (Table 1) listed in those publications. We found that 166 publications were shared with more than one basin or country, and were treated as separate. The result brought the total to 625 publications across all the African water basins. The majority of studies on freshwater snails in Africa were conducted in the Nile (136/625, 21.7%), followed by the Congo Basin (110/625, 17.6%) and Niger (78/625, 12.4%). The Orange Basin and Lake Tanganyika also produced a high number of studies (67/625, 10.7%) and (45/625, 7.2%), respectively. The number of studies on freshwater snails conducted across African water basins was disproportionate, with the top five water basins contributing 436 (69.7%) of the total studies of freshwater snails (Figure 3; Table S2; Ziganira and Downs 2024).

### 3.3 Field of Study Focus

The majority of freshwater snail study objectives related to conservation and taxonomy (553/791, 70%), followed by disease vector (162/791, 20.5%), with genetics/genomic/DNA barcoding/environmental DNA receiving focus lately (41/791, 5.2%). Studies focusing on geology and palaeontology (20/791, 2.5%), followed by climate change (12/791, 1.5%) and machine learning (3/791, 0.4%) (Figure 4; Table S3; Ziganira and Downs 2024).

### 3.4 Notable Pioneer of the 18th Century

The most notable pioneer in the study of plant and animal biodiversity in Africa in the 18th century was Michel Adanson, a French citizen from Aix‐en‐Provence, Southern France (Carteret 2012). At the end of 1748, Michel Adanson (1727–1806) was one of the most important naturalists of the eighteenth century. He left France for Senegal on an exploratory expedition as a botanist. He remained there for 5 years until 1753, moving from one trading post to another, collecting all kinds of biological specimens (plants and animals), including molluscs (Brown 1994; Carteret 2012; Paugy 2010). Nearly 260 years ago (1757), the first study volume dedicated to molluscs was published, and his real contribution covered 15 years of his work, from *Histoire naturelle du Sénégal* (published in 1757) to the *Cours d'histoire naturelle* (concerning zoology) which he taught between 1772 and 1774 (Carteret 2012; Fischer‐Piette 1942). From freshwater snails, Adanson described shells, anatomy and habitats using a binomial nomenclature system 1 year before Linnaeus introduced his own system (Brown 1994). He was the first to describe ‘*Le Coret*’ (*Afrogyrus coretus*, now *Hovorbis coretus*) and ‘Le Bulin’ (now *Bulinus senegalensis*), which became the type of the genus *Bulinus* (Brown 1994). Later, the discovery in Egypt of the life cycle of *Schistosoma haematobium* would make *Bulinus* one of the most intensively studied snails in the world (Brown 1994).

### 3.5 The Rise of Freshwater Malacology in Africa in the 19th Century

During the 19th century, some scientists did not share Adanson's interest in anatomy and conchology. As a result, many species were never named according to their shell differences, and important characteristics were left out (Brown 1994). The political scramble for Africa can also be seen in the way in which authors in Britain, France and Germany published their names for ‘new shells’ (Brown 1980, 1994). Numerous species were rapidly named from shells that reached Europe in increasing numbers during the 19th century (Brown 1980, 1994).

#### 3.5.1 Southern Africa, Especially South Africa

The publication of F. Krauss's ‘Die südafrikanischen Mollusken’ in 1848 was the first major step to studying the molluscan fauna in South Africa (Kilburn 1999). Towards the end of the 19th century, local interest increased when amateur malacologists such as W.H. Turton began collecting shells in the Eastern Cape and KwaZulu‐Natal Provinces to send them overseas (Kilburn 1999). The Deutsche Tiefsee Expedition (1898–1899) and the Cape and Natal governments Expedition (1897–1901) led by Pieter Faure were the first to scientifically sample molluscs in South Africa (Kilburn 1999). Most of the South African materials were first studied by overseas malacologists in the 19th century, but that rapidly changed in the 20th century and beyond.

#### 3.5.2 Central East‐Africa, Lake Tanganyika

Lake Tanganyika is the second deepest lake in the world (maximum 1470 m, south basin), after Lake Baikal in Russia. Situated at an altitude of 773 m and an area of 32,900 km^2^, Lake Tanganyika's varied shoreline has rocky outcrops and sandy beaches, swamps and estuaries (Brown 1994). The lake and its surrounding rivers have attracted international expeditions and subsequent malacological descriptions since the 1850s (Smith 1880a, 1880b), and some unresolved controversy about the origin and evolution of the lake and its fauna is still being studied (Glaubrecht and Strong 2004, 2007; Glaubrecht 2008; Krings et al. 2021; Michel et al. 1992, 2003; Michel 2000; West and Michel 2000; Wilson et al. 2004).

The first scientific expeditions to study freshwater organisms in tropical Africa were sent by the Royal Society in 1858 and 1899 to Lake Tanganyika, partly to investigate the remarkable prosobranch snails [*Melania* (now *Lavigeria*) *nasa* and *Lithoglyohys* (now *Spekia*) *zonata* (Woodward 1859)], collected by John Speke in 1858 (Brown 1994). With a total of 60 species (Brown and Mandahl‐Barth 1987), Lake Tanganyika has been a remarkable freshwater ecosystem in which to study speciation (Krings et al. 2021). Lake Tanganyika also presents some of the most ancient scientific and nonscientific studies on freshwater snails of Africa dated back to the 1880s (Ancey 1906; Bourguignat 1885, 1886, 1890; Germain 1908; Hubendick 1952; Hudleston 1904; Moore 1897, 1898a, 1898b, 1898c, 1899a, 1899b, 1903; Pelseneer 1886; Pilsbry and Bequaert 1927; Smith 1880a, 1880b, 1881, 1889, 1904; Schwetz and Dartevelle 1948; Yonge 1938). Most of the studies of freshwater snails conducted on Lake Tanganyika covered various fields of study, including biodiversity, conservation, ecology and taxonomy (Table 2).

Our literature search in Google Scholar, Web of Science, Scopus and the Biodiversity Heritage Library revealed that 26 studies were conducted between 1848 and 1899. These studies covered large regions of Africa, including Southern Africa, Central Africa and North‐East Africa, and the focus of these studies was on taxonomy, conservation and biodiversity (Table 3). Our literature search also found that many of the studies conducted in the 19th century (15/26, 57.7%) were conducted in or around the Lake Tanganyika region. In terms of molluscs, Lake Tanganyika belongs to the East African Great Lakes and is well known for harbouring a high proportion of endemic and morphologically distinct genera and a natural laboratory to study speciation (Krings et al. 2021).

**TABLE 3:** Some of the most outstanding freshwater snail publications highlighting the contributions of the 18th and the 19th centuries.

Author	Year	Field of study	Country/region
**18th century**			
M. Adanson	1757	Taxonomy	Senegal
**19th century**			
F. Krauss	1848	Taxonomy & Conservation	South Africa
G. Dunker	1853	Taxonomy & Conservation	Angola
S.P. Woodward	1859	Taxonomy & Conservation	Lake Tanganyika
E. Martens	1860	Taxonomy & Conservation	Mozambique
H. Dohrn	1864	Taxonomy & Conservation	Lake Victoria
H. Dohrn	1865	Taxonomy & Conservation	Lake Malawi
A. Morelet	1868	Taxonomy & Conservation	Angola
C.F. Jickeli	1874	Biodiversity	North‐East Africa
E. Martens	1879	Taxonomy & Conservation	Mozambique
E.A. Smith	1880a, 1880b	Taxonomy & Conservation	Lake Tanganyika
E.A. Smith	1881	Taxonomy & Conservation	Lake Tanganyika
M.J.R. Bourguignat	1885	Taxonomy & Conservation	Lake Tanganyika
M.J.R. Bourguignat	1886	Taxonomy & Conservation	Lake Tanganyika
P. Pelseneer	1886	Biodiversity	Lake Tanganyika
E.A. Smith	1889	Taxonomy & Conservation	Lake Tanganyika
M.J.R. Bourguignat	1889	Taxonomy & Conservation	North‐East Africa and East Africa
M.J.R. Bourguignat	1890	Taxonomy & Conservation	Lake Tanganyika
A. d'Ailly	1896	Taxonomy & Conservation	Cameroon
E. Martens	1897	Taxonomy & Conservation	East Africa
J.E.S. Moore	1897	Biodiversity	Lake Tanganyika
J.E.S. Moore	1898a	Biodiversity	Lake Tanganyika
J.E.S. Moore	1898b	Taxonomy	Lake Tanganyika
J.E.S. Moore	1898c	Biodiversity	Lake Tanganyika
R. Sturany	1898	Taxonomy & Conservation	Southern Africa
J.E.S. Moore	1899a, 1899b	Biodiversity	Lake Tanganyika

### 3.6 Modern Phase: 20th Century up to the End of the Second World War

The early 20th century in Africa was marked by the discovery that *Bulinus truncatus* (Audouin 1827) and *Biomphalaria alexandrina* (Ehrenberg 1831) are the intermediate hosts for the parasites causing human schistosomiasis in Egypt (Brown 1994; Leiper 1915, 1918). This discovery led to the modern study of African freshwater snails (Brown 1994; Table 4). Because of the high prevalence of tropical diseases such as malaria, health authorities on the continent were less preoccupied with human schistosomiasis. Nevertheless, two outstanding monographs were developed by amateur and professional malacologists describing the snails of the former Belgian Congo (now Democratic Republic of the Congo) (Pilsbry and Bequaert 1927) and South Africa (Brown 1994; Connolly 1939). Only after the Second World War did research on human schistosomiasis increase through the support of the World Health Organisation (WHO) (Brown 1994; Farooq 1973). There was also an urgent need to improve freshwater snail taxonomy, and a ‘Study Group’ on the Identification and Classification of the Bilharzia Snail Vectors in Africa was established in Paris in 1954 (Brown 1994). Through the financial support of the WHO and local national health authorities, African malacological research received a substantial boost, which advanced the knowledge of many snail species beyond the medical or veterinary fields of study (Brown 1994).

**TABLE 4:** Some of the most outstanding freshwater snail publications highlighting the contributions of the 20th century up to 1951.

Author	Year	Field of study	Country/Region
J.E.S. Moore	1903	Biodiversity	Lake Tanganyika
E.A. Smith	1904	Taxonomy & Conservation	Lake Tanganyika
W.H. Hudleston	1904	Geology	Lake Tanganyika
C.F. Ancey	1906	Taxonomy & Conservation	Lake Tanganyika
L. Germain	1908	Taxonomy & Conservation	Central‐North Africa, Lake Tanganyika
W. Kobelt	1909	Taxonomy & Conservation	North‐East Africa
M. Connolly	1912	Taxonomy & Conservation	Southern Africa (first really comprehensive checklist)
H.A. Pilsbry	1919	Taxonomy & Conservation	Belgian Congo (now D.R. Congo)
L. Germain	1920	Taxonomy & Conservation	East Africa
L. Germain	1923	Taxonomy & Conservation	East Africa
L. Germain	1925	Taxonomy & Conservation	Central and West Africa
M. Connolly	1925	Taxonomy & Conservation	Portuguese East Africa (now Mozambique)
H.A. Pilsbry & J. Bequaert	1927	Taxonomy & Conservation	Belgian Congo (now D.R. Congo)
M. Connolly	1930	Taxonomy & Conservation	South‐West Africa (now Namibia)
E. Degner	1934	Taxonomy & Conservation	West Africa
C.M. Yonge	1938	Conservation	Lake Tanganyika
M. Connolly	1939	Taxonomy & Conservation	Southern Africa
A. Mozley	1939	Ecology & Disease vector	Tanzania
J. Bequaert & W.J. Clench	1941	Taxonomy & Conservation	Belgian Congo (now D.R. Congo)
G. Bacci	1951	Taxonomy & Conservation	Abyssinia/Somalia (now Ethiopia and various states to the East)

### 3.7 Outstanding Contributions of the 20th Century Post‐1951

In the 1950s, there was a sudden increase in studying freshwater snails to primarily understand which snail species played intermediate hosts to several platyhelminth parasites of considerable significance to humans and livestock health (Brown 1994; De Bont and De Bont Hers 1952; Fain 1951a, 1951b; Gillet 1950; LaGrange and Fain 1952; Schwetz 1950, 1951a, 1951b; WHO 1958). Over 200 million people in the world suffer from schistosomiasis, of which about 80% live in sub‐Saharan Africa (Kokaliaris et al. 2022; WHO 1958). Through the financial support of the WHO and local national health authorities, African malacological research received a substantial boost, which advanced the knowledge of many snail species beyond the medical or veterinary fields of study (Brown 1994). The initiative greatly boosted African freshwater malacology and left an everlasting legacy on the continent (WHO 1958).

Human schistosomiasis (bilharzia) is the second most important tropical parasitic disease after malaria (WHO 2016) and ranked the most important water‐borne disease (Steinmann et al. 2006). Over 700 million people in 78 countries worldwide are at risk of infection, especially in sub‐Saharan Africa (WHO 2016), where up to 90% of rural communities are affected (Bergquist et al. 2017). Schistosomiasis is also probably one of the most neglected diseases, as funding priorities were redirected to HIV/AIDS, malaria and other new viruses (Hotez et al. 2007; Utzinger et al. 2009). However, in the last decade, there has been a growing interest (King et al. 2020; Shiff 2017; WHO 2012), and studies on freshwater snails have remarkably advanced in all the main fields of study such as taxonomy, ecology, distribution, snail‐borne parasitic infections and genetics (Brown 1994; Khalloufi et al. 2017; Klopper et al. 2002; Miller et al. 2023; Mukaratirwa et al. 1996a, 1996b; Mukaratirwa, Siegismund, Kristensen, et al. 1996; Ndassa, Mimpfoundi, and Elizabeth 2007; Zein‐Eddine et al. 2017). Presently, different ecological approaches, including DNA barcoding, environmental DNA and machine learning, are being used to control the distribution of intermediate host snails of schistosomiasis (Alzaylaee, Collins, Rinaldi, et al. 2020; Alzaylaee, Collins, Shechonge, et al. 2020; Lawton et al. 2018; Sato et al. 2018; Standley and Stothard 2012; Tabo et al. 2022, 2024; Tchakonte et al. 2023; Webster et al. 2013).

One of the most outstanding contributors in terms of African freshwater malacology during the past 70 years was David Brown, whose work covered the regions of North East Africa (Brown 1965; Brown et al. 1984), West Africa (Brown 1974; Brown and Kristensen 1993; Table S1; Ziganira and Downs 2024), Southern Africa (Brown 1967, 1978; Brown and Kristensen 1989; Brown et al. 1992), Lake Tanganyika (Brown and Mandahl‐Barth 1987; Table S4; Ziganira and Downs 2024) and East Africa (Brown et al. 1981; Table 4). David Brown, in his *Freshwater Snails of Africa and their Medical Importance* (two editions, 1980, revised 1994), provided an enormously valuable synthesis of a large body of work focusing on the taxonomy, biogeography, biology, ecology and, of course, parasitology of Africa's freshwater snail fauna. His standout contribution to African freshwater malacology has been significant in the last 70 years.

The other notable contributor to African freshwater malacology during the past 70 years was Georg Mandahl‐Barth, who published extensively on African freshwater bivalves and also established the Danish Bilharziasis Laboratory (DBL), Copenhagen, in 1964 (Mandahl‐Barth 1988; Table S4; Ziganira and Downs 2024). His work covered the regions of Central Africa (Mandahl‐Barth 1968; Mandahl‐Barth et al. 1972, 1974), South‐East Africa (Mandahl‐Barth 1968, 1972), Lake Tanganyika (Brown and Mandahl‐Barth 1987) and East Africa (Mandahl‐Barth 1954). Other prominent role players who have contributed from a broader malacological rather than purely parasitological or medical perspective include Thomas Kristensen and Henry Madsen at the Danish Bilharziasis Laboratory (DBL), Copenhagen, which has covered the regions of North East Africa (DBL 1983, 1986; Madsen et al. 1988; Makura and Kristensen 1991; Table S4; Ziganira and Downs 2024), North West Africa (Kristensen 1985), West Africa (Madsen et al. 1987), Central Africa (DBL 1982), Southern East Africa (DBL 1977) and East Africa (DBL 1987). Chris Wright of the Taxonomy Unit of the Natural History Museum, London [formerly the British Museum (Natural History)] research covered regions of Central Africa (Wright 1965) and Southern Africa (Wright 1963). Ferdinand Starmühlner of the Institut für Zoologie, Vienna, work's focused on the Indian Ocean Islands (Starmühlner 1969, 1976a, 1976b, 1977, 1983, 2006). Dirk van Damme (University of Ghent, Belgium) has concentrated his research on Cenozoic fossils in the regions of North East Africa (Van Damme 1984), North West Africa (Van Damme 1984) and West Africa (Van Damme 1984). In Southern Africa, Chris Appleton was very active with his team at the Institute for Zoological Research, North West University, Potchefstroom, South Africa, since the 1970s (Appleton 1975, 1977a, 1977b, 1984; Appleton and Stiles 1976; Appleton and Bruton 1979; Appleton et al. 1983; Appleton and Branch 1989) and is still contributing to African freshwater malacology (Appleton and Miranda 2015; Miranda et al. 2016). Christine Betterton of Bayero University in Kano, Nigeria, has extensively worked on *Bulinus* with regards to disease transmission in Nigeria, West Africa (Betterton et al. 1983; Betterton, Ndifon, and Oyeyi 1988; Betterton, Ndifon, and Tan 1988; Betterton 1984a, 1984b; Ndifon et al. 1988). More recently, Ellinor Michel (Natural History Museum, London) and her colleagues (Nyanza Project, Kelly West and Jon Todd) have contributed much in relation to the Rift Valley Lakes, in particular Lake Tanganyika (Michel 2000; Michel et al. 1992, 2003; West and Michel 2000). The fields of biodiversity and conservation, as well as taxonomy, have received substantial attention in the last few decades (Tables 2 and 3; Table S4; Ziganira and Downs 2024).

### 3.8 Modern Trends of the 21st Century

Alongside conventional taxonomy and systematic and biodiversity conservation studies, there has been a steady rise in the number of genetic and cytogenetic studies with phylogenetic or taxonomic applications for snail species in Africa. Since the 1970s, there has been a body of work using different modern methods on molecular description and identification studies of *Bulinus* and *Biomphalaria* snails with regard to disease transmission across Africa (Akinwale et al. 2011; Alharbi et al. 2022; Amarir et al. 2023; Burch et al. 1979; DeJong 2003; Doums et al. 1996; Goll 1981; Mimpfoundi and Greer 1990a, 1990b, 1990c; Mudavanhu et al. 2024; Mukaratirwa et al. 1996a, 1996b, 2023; Mukaratirwa, Siegismund, Kristensen, et al. 1996; Njiokou et al. 1993; Pringle et al. 1971; Rollinson and Southgate 1979; Standley et al. 2014).

#### 3.8.1 African Malacologists

In many countries in Africa today, there are staff trained in malacological research in universities and public health laboratories (Brown 1994; De Kock et al. 1974; De Kock and Wolmarans, 2005a,b, 2006, 2007a,b; 2008, 2009, 2017; Ibikounlé et al. 2009, 2013; Ibikounlé, Gbédjissi, et al. 2014; Ibikounlé, Ogouyèmi‐Hounto, et al. 2014; Miranda and Perissinotto 2014; Ndassa and Mimpfoundi 2006; Wembo Ndeo et al. 2017, 2018, 2020). We can add a growing number of younger malacologists equipped with molecular skills who have already significantly contributed to our understanding of the taxonomy and phylogenetics of African freshwater molluscs, particularly gastropods. For example, Oscar Wembo Ndeo's research has focused on gastropods of Lake Tanganyika and Central Africa (Wembo Ndeo et al. 2017, 2018, 2020). Samson Mukaratirwa has focused on the control of snail disease vectors using molecular approaches, including modern tools such as environmental DNA in Zimbabwe and South Africa (Chingwena et al. 2004; Malatji et al. 2019, 2021; Mbereko et al. 2023; Mukaratirwa et al. 1996a, 1996b, 2004, 2023; Mukaratirwa, Siegismund, Kristensen, et al. 1996). Aspire Mudavanhu is using molecular approaches to understand freshwater snail species and parasite diversity and interactions in Zimbabwe (Mudavanhu et al. 2024; Schols et al. 2020). Others include Siméon Tchakonté, Arouna Ndassa and Remy Mimpfoundi who have focussed on freshwater snails of the Central African region (Mimpfoundi et al. 1986; Mimpfoundi and Greer 1990a, 1990b, 1990c; Ndassa and Mimpfoundi 2006; Ndassa, Mimpfoundi, and Elizabeth 2007; Ndassa, Mimpfoundi, Gake, et al. 2007; Tchakonte et al. 2023). Fred Chibwana's research has focused on the East African region (Chibwana et al. 2020; Tumwebaze et al. 2022). Maxwell Barson's research areas include taxonomy and ecology of freshwater aquatic animal parasites (Muzarabani et al. 2024; Schols et al. 2020, 2021). Moudachirou Ibikounlé has been contributing to the study of snails as vectors of infectious diseases (Ibikounlé et al. 2009, 2013; Ibikounlé, Gbédjissi, et al. 2014; Ibikounlé, Ogouyèmi‐Hounto, et al. 2014). Nelson A.F. Miranda has extensively worked on invasive gastropods such as *Terebia granifera* in South Africa (Miranda et al. 2010, 2011a, 2011b; Miranda and Perissinotto 2014). Similarly, Kenné de Kock's contribution has been on invasive freshwater snails and disease vectors (De Kock et al. 1974, 2003, 2004; De Kock and Wolmarans, 2005a,b, 2006, 2007a,b; 2008, 2009, 2017). Moses J. Chimbari's research has been on biological control of the snail vector of schistosomiasis in Botswana and Zimbabwe (Chimbari 2012; Chimbari et al. 1997, 2020; Chingwena et al. 2004; Mbereko et al. 2023). Our list cited above is not exhaustive as there are many other African malacologists (professionals and students) based in Africa who are significantly contributing to the field in their respective countries and with collaborations on the continent and with overseas institutions (Table S4; Ziganira and Downs 2024).

#### 3.8.2 Collaborations With Overseas Malacologists

Some overseas laboratories have worked on African freshwater snails, and some African malacologists have been trained in some of these institutions. Kenneth A. Hayes and Robert H. Cowie have worked on the evolutionary biology of apple snails (Ampullariidae) associated with their Gondwana origin (Hayes, Cowie, Jørgensen, et al. 2009; Hayes, Cowie, and Thiengo 2009). Randall Dejong has contributed to molecular biology and disease vector studies (DeJong 2003; DeJong et al. 2001; Morgan et al. 2002, 2003). Matthias Glaubrecht has been working on gastropods of Lake Tanganyika, West Africa and Madagascar (Glaubrecht and Strong 2004, 2007; Glaubrecht 2008; Köhler and Glaubrecht 2010; Krings et al. 2021; Wilson et al. 2004). Thomas K. Kristensen and Henry Madsen have been very active across all regions in Africa (Kristensen 1985; Kristensen and Ogunnowo 1987; Kristensen and Brown 1999; Madsen 1983, 1992; Madsen and Stauffer 2011; Madsen et al. 1987, 1988, 2010). They have extensively collaborated with African malacologists on a number of projects and participated in the training of many African students (Chimbari et al. 1997; Chingwena et al. 2002, 2004; Lange et al. 2013; Malatji et al. 2019; Mukaratirwa, Siegismund, Kristensen, et al. 1996; Nalugwa et al. 2011; Plam et al. 2008; Sengupta et al. 2009; Table S4; Ziganira and Downs 2024). Ellinor Michel and her colleagues (Nyanza Project, Kelly West and Jon Todd) have contributed much about the Rift Valley Lakes, in particular, Lake Tanganyika (Michel 2000; Michel et al. 2003, 1992; West and Michel 2000; Table S4; Ziganira and Downs 2024). Christian Albrecht has been very active in Central Africa and East Africa, while Roland Schultheiß, Thomas Wilke and Bert Van Bocxlaer have covered palaeontology and climate change aspects of freshwater snails in Africa (Albrecht et al. 2004; Catharina et al. 2022; Chibwana et al. 2020; Hayes, Cowie, Jørgensen, et al. 2009; Sands et al. 2022; Schultheiß et al. 2009, 2011, 2014; Tabo et al. 2022, 2024; Tumwebaze et al. 2022; Van Bocxlaer 2005, 2020; Van Bocxlaer and Albrecht 2015; Van Bocxlaer and Van Damme 2009; Van Bocxlaer et al. 2008, 2011, 2012, 2015, 2020; Wembo Ndeo et al. 2017, 2020; Table S4; Ziganira and Downs 2024). Martin J. Genner and his team have worked on aspects of the evolutionary and invasive biology of gastropods of Lake Malawi (Genner and Michel 2003; Genner et al. 2007, 2008). Tine Huyse and Ruben Schols have been working in collaboration with African researchers based in Central, East and Southern Africa (Brees et al. 2021; Kapour, Wambui, Madinga, et al. 2024a; Kapour, Wambui, Schols, et al. 2024b; Maes et al. 2022; Mudavanhu et al. 2024; Muzarabani et al. 2024; Schols et al. 2020, 2021, 2023; van der Deure et al. 2024). It is also worth mentioning that this list is not exhaustive, and the degree of collaboration between these laboratories and universities in Africa and overseas has been a positive sign showing a mutually beneficial exchange of ideas.

#### 3.8.3 Major Shifts in Methodology

The study focuses on taxonomy, biodiversity and conservation, as well as disease vectors, and has laid the foundation for malacology research in Africa. These interests are still growing, except in the taxonomy field, which is concerning. The population of molluscan taxonomists worldwide is decreasing, and similar trends are observed in Africa (Bouchet et al. 2016). Presently, no academic programme is dedicated to training the next generation of taxonomists in Africa. With the old generation of taxonomists, either deceased or retired, capacity building still has a long way to go, as students publishing one or two papers from their dissertations is not enough to build sustainable capacity in this field. Things could have been different if taxonomical research had received institutional support from academic institutions and funding agencies for baseline alpha‐taxonomy (Bouchet et al. 2016). However, genetics has been helping to close this gap. Molecular studies of African freshwater snails have been emphasised in the past years. Genetics has been used to describe attributes of a group of taxa, solve systematic or phylogenetic questions and assess the genetic variability of a species that could potentially transmit human diseases (Bouchet et al. 2016). Unfortunately, this is not a definite solution because not all snail species have sequences in BOLD and/or GenBank (Bouchet et al. 2016). Presently, major shifts in methodology include the use of DNA barcoding on some medically important freshwater snail genera, which have revealed multiple invasion events (Lawton et al. 2018) or some sublineages in various locations in Africa (Standley and Stothard 2012; Webster et al. 2013). Other shifts in methodology include the use of environmental DNA (eDNA) (Alzaylaee, Collins, Rinaldi, et al. 2020; Alzaylaee, Collins, Shechonge, et al. 2020; Sato et al. 2018) and, most recently, machine learning approaches using artificial intelligence are being explored (Tabo et al. 2022, 2024; Tchakonte et al. 2023).

## 4 Conclusions

Our review of the literature on freshwater snails of Africa attempted to document the major progress that has shaped the malacological research on the continent. Starting in the 18th century in Senegal, overseas researchers initiated and developed African malacology. Later in the 20th century, local interest has been on the rise up to this day. Taxonomy, biodiversity and conservation were important to set the foundation, and over time, disease vectors, ecology and palaeontology were added. New aspects, such as climate change, were also investigated. The 21st century has seen the rise of new methodologies such as genetics (DNA barcoding) and environmental DNA, as well as machine learning approaches. With the global decline of taxonomists, it must be stressed that African malacology must evolve and adapt to this new technological era. New advances will depend mainly on new collections coming from little‐explored areas, or armed conflict regions such as the Congo Basin, the Ethiopian Highlands and West Africa. The latest taxonomic updates, the updated IUCN status of important African freshwater snail species and data availability and access would be important tools for future research. Additionally, development programmes, collaboration and institutional support from academic institutions and funding agencies would be a big boost to the increased study of freshwater molluscs.

## Author Contributions

**Matabaro Ziganira:** conceptualization (equal), data curation (lead), formal analysis (lead), investigation (lead), methodology (lead), writing – original draft (lead). **Colleen T. Downs:** conceptualization (equal), funding acquisition (equal), project administration (equal), resources (equal), supervision (equal), writing – review and editing (equal).

## Conflicts of Interest

The authors declare no conflicts of interest.

## Supporting information


Table S1

Table S2

Table S3

Table S4

## Data Availability

Data for this study are presented in the Supporting Information or Dryad (Ziganira, and Downs 2024: https://doi.org/10.5061/dryad.cjsxksndq).
