# Supplementary material for: Significant Progress in the Study of African Freshwater Snails Over the Past 260 Years
Source: Ecol Evol. 2025 Feb 20;15(2):e71031. doi: 10.1002/ece3.71031 (PMC11842873; doi:10.1002/ece3.71031)
Supplement: Supplementary file 1 — Table S1 [file ECE3-15-e71031-s002.docx]

| **Supplementary Information Table S1. Summary of some publications of the extensive literature of few outstanding contributions between the 1950s and the 1990s.** | | | |
| --- | --- | --- | --- |
| North East Africa (Egypt, Sudan, South Sudan and Ethiopia) | | | |
| **Author** | **Year** | **Field of study** | **Country/ Region** |
| D.S. Brown | 1965 | Taxonomy & Conservation | Ethiopia |
| E. Tchernov | 1971 | Taxonomy & Conservation | Egypt |
| H. Itagaki et al. | 1975 | Taxonomy, Ecology & Disease vector | Ethiopia |
| Danish Bilharziasis Laboratory (DBL) | 1983 | Taxonomy & Conservation | North East Africa |
| D.S. Brown | 1984 | Taxonomy, Ecology & Disease vector | South Sudan |
| Danish Bilharziasis Laboratory (DBL) | 1986 | Taxonomy & Conservation | North East Africa |
| H. Madsen et al. | 1988 | Ecology | Sudan |
|  |  |  |  |
| North West Africa (Algeria, Morocco, Tunisia, Libya) | | | |
| **Author** | **Year** | **Field of study** | **Country/ Region** |
| J. Dupouy et al. | 1980 | Ecology | North West Africa |
| D. Van Damme | 1984 | Biogeography and Palaeoecology | Northern Africa |
| T.K. Kristensen | 1985 | Taxonomy & Conservation | North West Africa |
| C. Meier-Brook et al. | 1987 | Ecology | North West Africa |
|  |  |  |  |
| West Africa (From Lake Chad to coasts of Mauritania and Senegal) | | | |
| **Author** | **Year** | **Field of study** | **Country/ Region** |
| E. Binder | 1957 | Taxonomy & Conservation | Côte d'Ivoire |
| C. Lévêque | 1967 | Biodiversity & Conservation | Lake Chad |
| E. Binder | 1968 | Taxonomy & Conservation | Côte d'Ivoire |
| D.S. Brown | 1974 | Biodiversity & Conservation | Lake Chad |
| B. Hubendick | 1977 | Biodiversity & Conservation | Sierra Leone |
| B. Sellin et al. | 1980 | Ecology & Disease vector | West Africa |
| C. Betterton | 1984 | Ecology & Disease vector | Nigeria |
| H. Madsen et al. | 1987 | Ecology & Disease vector | Mali |
| G.T. Ndifon & F.M.A. Ukoli | 1989 | Ecology | Nigeria |
| D.S. Brown & T.K. Kristensen | 1993 | Taxonomy & Conservation | West Africa |
|  |  |  |  |
| Central Africa (Cameroon, Central African Republic, Gabon, Congo, D.R. Congo) | | | |
| **Author** | **Year** | **Field of study** | **Country/ Region** |
| C.A. Wright | 1965 | Taxonomy & Conservation | Cameroon |
| G. Mandahl-Barth | 1968 | Taxonomy & Conservation | DR Congo |
| G. Mandahl-Barth et al. | 1972 | Taxonomy & Conservation | DR Congo |
| G. Mandahl-Barth et al. | 1974 | Ecology & Disease vector | DR Congo |
| Danish Bilharziasis Laboratory (DBL) | 1982 | Taxonomy & Conservation | Central Africa |
| B. Baluku et al. | 1989 | Biodiversity & Conservation | DR Congo |
|  |  |  |  |
| Southern Africa (Angola and area south of Zambezi River) | | | |
| **Author** | **Year** | **Field of study** | **Country/ Region** |
| C.A. Wright | 1963 | Taxonomy & Conservation | Angola |
| C.H.J. Schutte & G.H. Frank | 1964 | Ecology & Disease vector | South Africa |
| J.A. Van Eeden & C. Combrink | 1966 | Ecology & Disease vector | South Africa |
| D.S. Brown | 1967 | Taxonomy & Conservation | South Africa |
| G. Oberholzer & J.A. Van Eeden | 1967 | Biodiversity & Conservation | South Africa |
| J.F. Prinsloo & J.A. Van Eeden | 1973 | Ecology & Disease vector | Lesotho |
| K.N. De Kock et al. | 1974 | Biodiversity & Conservation | South Africa |
| S.J. Pretorius et al. | 1975 | Ecology | South Africa |
| C.C. Appleton | 1975 | Ecology & Disease vector | South Africa |
| C.C. Appleton | 1977 | Taxonomy & Conservation | South Africa |
| D.S. Brown | 1978 | Biogeography & Ecology | Southern Africa |
| C. Machena & N. Kautsky | 1988 | Biodiversity & Conservation | Zimbabwe |
| D.S. Brown & T.K. Kristensen | 1989 | Taxonomy & Conservation | Southern Africa |
| O. Makura & T.K. Kristensen | 1991 | Biodiversity & Conservation | Zimbabwe |
| D.S. Brown et al. | 1992 | Biodiversity & Conservation | Namibia & Botswana |
|  |  |  |  |
| South East Africa (Malawi, Northern Mozambique, Zambia) | | | |
| **Author** | **Year** | **Field of study** | **Country/ Region** |
| J.F. Azevedo et al. | 1961 | Taxonomy & Conservation | Mozambique |
| G. Mandahl-Barth | 1968 | Taxonomy & Conservation | Zambia |
| G. Mandahl-Barth | 1972 | Taxonomy & Conservation | Malawi |
| Danish Bilharziasis Laboratory (DBL) | 1977 | Taxonomy & Conservation | South East Africa |
| C. Machena & N. Kautsky | 1988 | Biodiversity & Conservation | Zambia |
|  |  |  |  |
| Lake Tanganyika |  |  |  |
| **Author** | **Year** | **Field of study** | **Country/ Region** |
| E. Leloup | 1953 | Ecology | Lake Tanganyika |
| D.S. Brown & G. Mandahl-Barth | 1987 | Taxonomy & Conservation | Lake Tanganyika |
|  |  |  |  |
| East Africa (Burundi, Rwanda, Tanzania, Uganda, Kenya and Somalia) | | | |
| **Author** | **Year** | **Field of study** | **Country/ Region** |
| G. Mandahl-Barth | 1954 | Taxonomy & Conservation | Uganda |
| D.S. Brown et al. | 1981 | Ecology & Disease vector | Kenya |
| Danish Bilharziasis Laboratory (DBL) | 1987 | Taxonomy & Conservation | East Africa |
|  |  |  |  |
| Indian Ocean Islands (Comoros, Seychelles, Mauritius and Madagascar and Réunion) | | | |
| **Author** | **Year** | **Field of study** | **Country/ Region** |
| F. Starmühlner | 1969 | Taxonomy & Conservation | Madagascar |
| A.A. Degrémont | 1973 | Ecology & Disease vector | Madagascar |
| F. Starmühlner | 1976a | Taxonomy & Conservation | Comoros |
| F. Starmühlner | 1976b | Taxonomy & Conservation | Mauritius |
| F. Starmühlner | 1977 | Taxonomy & Conservation | Réunion |
| N. Barré et al. | 1982 | Biodiversity & Conservation | Réunion |
| F. Starmühlner | 1983 | Taxonomy & Conservation | Indian Ocean Islands |
| T. Backeljau et al. | 1986 | Taxonomy & Conservation | Comoros |
| J. Julvez et al. | 1990 | Biodiversity & Conservation | Comoros |
