# Supplementary material for: Significant Progress in the Study of African Freshwater Snails Over the Past 260 Years
Source: Ecol Evol. 2025 Feb 20;15(2):e71031. doi: 10.1002/ece3.71031 (PMC11842873; doi:10.1002/ece3.71031)
Supplement: Supplementary file 2 — Table S2 [file ECE3-15-e71031-s004.docx]

| **Supplementary Information Table S2. Distribution of freshwater studies conducted across major African water basins between 1757 and 2024.** | | | | |
| --- | --- | --- | --- | --- |
| **Assessment was done on 625 studies, as 166 studies were conducted across multiple water bodies and were not counted for distribution in a specific basin.** | | | | |
| **Basin Name** | **Number of studies** | **%** |  |  |
| Nile | 136 | 21.7 |  |  |
| Congo | 110 | 17.5 |  |  |
| Niger | 78 | 12.4 |  |  |
| Lake Chad | 17 | 2.7 |  |  |
| Zambezi | 28 | 4.4 |  |  |
| Orange | 67 | 10.7 |  |  |
| Okavango | 16 | 2.6 |  |  |
| Limpopo | 15 | 2.4 |  |  |
| Volta | 36 | 5.7 |  |  |
| Senegal | 24 | 3.8 |  |  |
| Lake Tanganyika | 45 | 7.2 |  |  |
| Lake Victoria | 16 | 2.6 |  |  |
| Lake Malawi | 32 | 5.1 |  |  |
| Others | 166 | 26.5 |  |  |
|  |  |  |  |  |
