# Supplementary material for: Significant Progress in the Study of African Freshwater Snails Over the Past 260 Years
Source: Ecol Evol. 2025 Feb 20;15(2):e71031. doi: 10.1002/ece3.71031 (PMC11842873; doi:10.1002/ece3.71031)
Supplement: Supplementary file 3 — Table S3 [file ECE3-15-e71031-s003.docx]

| **Supplementary Information Table S3. Total number of freshwater snail study objectives recorded across African water basins between 1757 and 2024.** | | | | | | |
| --- | --- | --- | --- | --- | --- | --- |
| **Year interval** | **Taxonomy/Conservation** | **Geology/ Palaeontology** | **Disease vector** | **Climate change** | **Genetic/Genomic/DNA barcoding/eDNA** | **Machine learning** |
| 1757-1800 | 1 | 0 | 0 | 0 |  | 0 |
| 1801-1850 | 0 | 0 | 0 | 0 | 0 | 0 |
| 1851-1900 | 26 | 0 | 0 | 0 | 0 | 0 |
| 1901-1950 | 20 | 2 | 6 | 0 | 0 | 0 |
| 1951-2000 | 289 | 4 | 75 | 2 | 15 | 0 |
| 2001-2024 | 217 | 14 | 81 | 10 | 26 | 3 |
| Total | 553 | 20 | 162 | 12 | 41 | 3 |
| Percentage (%) | 70 | 2.5 | 20.5 | 1.5 | 5.2 | 0.4 |
|  |  |  |  |  |  |  |
