# Supplementary material for: Significant Progress in the Study of African Freshwater Snails Over the Past 260 Years
Source: Ecol Evol. 2025 Feb 20;15(2):e71031. doi: 10.1002/ece3.71031 (PMC11842873; doi:10.1002/ece3.71031)
Supplement: Supplementary file 4 — Table S4 [file ECE3-15-e71031-s001.docx]

| **Supplementary Information Table S3. Summary of freshwater snail publications in Africa from 1757-2024** | | |
| --- | --- | --- |
| **No.** | **Full Reference** |  |
| 1 | AbdAllah, A.T., (2006). Effects of dissolved lead and copper on the freshwater prosobranch Lanistes carinatus. *Malacologia,* 48(1-2), 27-34. | |
| 2 | Abdel-Halim, K.Y., Mona, M. H., Giesy, J. P., Shouker, F. A., & Osman, S. R., (2019). Cytotoxic effects of persistent organic pollutants on the freshwater snail (Lanistes carinatus) in Kafr El-Zayat, Egypt. *Environmental monitoring and assessment*, 191(11), 699. | |
| 3 | Abou-El-Naga, I. F., (2014). Meta-analysis indicates lack of local adaptation of Schistosoma mansoni to Biomphalaria alexandrina in Egypt. *Parasitology Research* 113, 1185–1194. | |
| 4 | Adanson, M., (1757). Histoire Naturelle du Senégal. Coquillages. Paris. | |
| 5 | Ademolu, K. O., Akintola, M. Y., Olalonye, A. O., & Adelabu, B. A., (2015). Traditional utilization and biochemical composition of six mollusc shells in Nigeria. *Revista De Biologia Tropical* 63(2), 459-464. | |
| 6 | Adewunmi, C. O., Becker, W., Kuehnast, O., Oluwole, F., & Dörfler, G., (1996). Accumulation of copper, lead and cadmium in freshwater snails in southwestern Nigeria. *Science of The Total Environment*, 193(1), 69-73. | |
| 7 | Adewunmi, C. O., Furu, P., & Fagbola, M., (1990). Endemicity and seasonality of transmission of human Schistosomiasis in ile-ife, south western Nigeria. *Tropical Medicine and Parasitology*, 41(4), 443-444. | |
| 8 | Adriko, M., Standley, C.J., Tinkitina, B., Mwesigwa, G., Kristensen, T. K., Stothard, J. R., & Kabatereine, N. B., (2013). Compatibility of Ugandan Schistosoma mansoni isolates with Biomphalaria snail species from Lake Albert and Lake Victoria. *Acta Tropica*, 128(2), 303-308. | |
| 9 | Agboho, P. A., Gbedjissi, G. L., Akogbeto, M. C., Murphy, W. L., & Vala, J. C., (2019). Life Cycle and Biology of the Afrotropical Snail-Killing Fly Sepedon (Sepedomyia) nasuta Verbeke (Diptera: Sciomyzidae). *Proceedings of the Entomological Society of Washington*, 121 (3), 345-365. | |
| 10 | Agbolade, O. M., Akinboye, D. O., & Adebambo, A. A., (2004). Human urinary schistosomiasis transmission foci and period in an endemic town of Ijebu North, southwest Nigeria. *Tropical Biomedicine*, 21, 15-22. | |
| 11 | Agola, E. L., Mwangi, I. N., Maina, G. M., Kinuthia, J. M., & Mutuku, M. W., (2021). Transmission sites for Schistosoma haematobium and Schistosoma bovis identified in localities within the Athi River basin of Kenya using a PCR-RFLP assay. *Heliyon*, 7(2). | |
| 12 | Ailly, A. d’, (1896). Contributions à la connaissance des mollusques terrestres et d’eau douce de Kaméroun. *Bihang till Kongliga Svenska Vetenskaps-Akademiens Handlingar,* 22(4), 21-137. | |
| 13 | Aka, N'Da A., Adoubryn, K., Rondelaud, D., & Dreyfuss, G., (2008). Human Paragonimiasis in Africa. *Annals of African Medicine*, 7(4), 153-162. | |
| 14 | Akindele, E. O., Ehlers, S. M., & Koop, J. H. E., (2019). First empirical study of freshwater microplastics in West Africa using gastropods from Nigeria as bioindicators. *Limnologica*, 78, 125708. | |
| 15 | Akinwale, O. P., Kane, R. A., Rollinson, D., Stothard, J. R., Ajayi, M. B., Akande, D. O., Ogungbemi, M. O., Duker, C., Gyang, P. V., & Adeleke, M. A., (2011). Molecular approaches to the identification of *Bulinus* species in south-west Nigeria and observations on natural snail infections with schistosomes. *Journal of Helminthology*, 85(3), 283-93. | |
| 16 | Akogun, O. B., & Akogun, M. K., (1996). Human behaviour, water usage and schistosomiasis transmission in a small settlement near Yola, Nigeria. *Annals of Tropical Medicine and Parasitology*, 90(3), 303-311. | |
| 17 | Albert, J. S., Destouni, G., Duke-Sylvester, S. M., Magurran, A. E., Oberdorff, T., Reis, R. E., Winemiller, K. O., & Ripple, W. J., (2021). Scientists’ warning to humanity on the freshwater biodiversity crisis. *Ambio*, 50, 85–94. | |
| 18 | Albrecht, C., Wilke, T., Kuhn, K. & Streit, B., (2004). Convergent evolution of shell shape in freshwater limpets: the African genus *Burnupia*. *Zoological Journal of the Linnean Society,* 140, 577-586. | |
| 19 | Alharbi, M. H., Iravoga, C., Kayuni, S. A., Cunningham, L., LaCourse, E. J., Makaula, P., & Stothard, J. R., (2022). First Molecular Identification of Bulinus africanus in Lake Malawi Implicated in Transmitting Schistosoma Parasites. T*ropical Medicine and Infectious Disease*, 7(8) | |
| 20 | Aliu, O. O., Akindele, E. O., & Adeniyi, I. F., (2020). Biological assessment of the headwater rivers of Opa Reservoir, Ile-Ife, Nigeria, using ecological methods. *Journal of Basic and Applied Zoology*, 81(11). | |
| 21 | Aljahdali, M. O., & Alhassan, A. B., (2020). Spatial Variation of Metallic Contamination and Its Ecological Risk in Sediment and Freshwater Mollusk: Melanoides tuberculata (Muller, 1774) (Gastropoda: Thiaridae). *Water*, 12(1), 206. | |
| 22 | Alkalay, A. S., Rosen, O., Sokolow, S. H., Faye, Y. P. W., Faye, D. S., Aflalo, E. D., Jouanard, N., Zilberg, D., Huttinger, E., & Sagi, A., (2014). The Prawn Macrobrachium vollenhovenii in the Senegal River Basin: Towards Sustainable Restocking of All-Male Populations for Biological Control of *Schistosomiasis*. *PLoS Neglected Tropical Diseases*, 8 (8), 1-13. | |
| 23 | Allan, F., Ame, S. M., Tian-Bi, Y. T., Hofkin, B. V., Webster, B. L., Diakité, N. R., N'Goran, E. K., Kabole, F., Khamis, I. S., Gouvras, A. N., Emery, A. M., Pennance, T., Rabone, M., Kinung'hi, S., Hamidou, A. A., Mkoji, G. M., McLaughlin, J. P., Kuris, A. M., Loker, E. S., Knopp, S., & Rollinson, D., (2020). Snail-Related Contributions from the Schistosomiasis Consortium for Operational Research and Evaluation Program Including Xenomonitoring, Focal Mollusciciding, Biological Control, and Modeling. The American journal of tropical medicine and hygiene, 103 (1), 66-79. | |
| 24 | Allan, F., Sousa-Figueiredo, J. C., Emery, A. M., Paulo, R., Mirante, C., Sebastião, A., Brito, M., & Rollinson, D., (2017). Mapping freshwater snails in north-western Angola: distribution, identity and molecular diversity of medically important taxa. *Parasites & Vectors*, 10, 1-10. | |
| 25 | Alzaylaee, H., Collins, R. A., Rinaldi, G., Shechonge, A., Ngatunga, B., Morgan, E. R., & Genner, M. J., (2020)a. Schistosoma species detection by environmental DNA assays in African freshwaters. PLoS Neglected Tropical Diseases, 14(3), e0008129. https://doi.org/10.1371/journal.pntd.0008129 | |
| 26 | Alzaylaee, H., Collins, R. A., Shechonge, A., Ngatunga, B., Morgan, E. R., & Genner, M. J., (2020)b. Environmental DNA-based xenomonitoring for determining Schistosoma presence in tropical freshwaters. Parasites & Vectors, 13, 63. https://doi.org/10.1186/s13071-020-3941-6 | |
| 27 | Alzaylaee, H., Collins, R.A., Shechonge, A., Ngatunga, B., Morgan, E.R., Genner, M.J., (2020)b. Environmental DNA-based xenomonitoring for determining Schistosoma presence in tropical freshwaters. Parasites & Vectors 13, 63. https://doi.org/10.1186/s13071-020-3941-6 | |
| 28 | Alzurfi, S. K., Algburi, J. B., Taher, M. M., Alhachami, L. H., & Zwain, K. A., (2019). Mollusca diversity in aquatic ecosystem. *ARPN Journal of Engineering and Applied Sciences*, 14, 7277–7283. | |
| 29 | Amarir, F., Balahbib, A., & Sebti, F., (2023). Geographical distribution and molecular survey of freshwater snail intermediate hosts of Schistosoma haematobium by DraI/Sh73 PCR in eliminated foci of Morocco. *Tropical Doctor*, 53(1), 128-133. | |
| 30 | Amin, M. A., Fenwick, A., Osgerby, J. M., Warley, A. P., & Wright, A. N., (1976). Large-scale snail control trial with trifenmorph in the Gezira irrigation scheme, Sudan. *Bulletin of the World Health Organization*, 54(5), 573–585. | |
| 31 | Ancey, C. F., (1906). Reflexions sur la fauna malacologique du Lac Tanganika et cataloque des mollusques de ce lac. *Bulletin biologique de la France et de la Belgique,* 5(9), 229–270. | |
| 32 | Andrus, P. S., Stothard, J. R., & Wade, C. M., (2023). Seasonal patterns of Schistosoma mansoni infection within Biomphalaria snails at the Ugandan shorelines of Lake Albert and Lake Victoria. *Plos Negelected Tropical Diseases*, 17(8). | |
| 33 | Angora, E. K., Allienne, J. F., Rey, O., Menan, H., Touré, A. O., Coulibaly, J. T., Raso, G., Yavo, W., N'Goran, E. K., Utzinger, J., Balmer, O., & Boissier, J., (2020). High prevalence of *Schistosoma haematobium × Schistosoma bovis* hybrids in schoolchildren in Côte d'Ivoire. *Parasitology*, 147(3), 287-294. | |
| 34 | Appleton, C. C., (1975). The influence of stream geology on the distribution of bilharzia host snails, *Biomphalaria pfeifferi* and *Bulinus (Physopsis)* sp. *Annals of Tropical Medicine and Parasitology,* 69, 241-255. | |
| 35 | Appleton, C. C., (1977)a. The influence of temperature on the life-cycle and distribution of *Biomphalaria pfeifferi* (Krauss, 1948) in South-Eastern Africa. *International Journal for Parasitology*, 7(5), 335-345. | |
| 36 | Appleton, C. C., (1977)b. The freshwater Mollusca of Tongaland, with a note on molluscan distribution in Lake Sibayi. *Annals of the Natal Museum*, 23, 129-144. | |
| 37 | Appleton, C. C., (1984). Schistosome dermatitis - an unrecognized problem in South Africa? S*outh African Medical,* 65(12), 467-469. | |
| 38 | Appleton, C. C., & Stiles, G., (1976). Geology and geomorphology in relation to distribution of snail intermediate hosts of bilharzia in South Africa*. Annals of Tropical Medicine and Parasitology,* 70(2), 189-198. | |
| 39 | Appleton, C. C., & Bruton, M. N., (1979). Epidemiology of Schistosomiasis in the vicinity of lake Sibaya, with a note on other areas of Tongaland (Natal, South Africa). *Annals of Tropical Medicine and Parasitology, 73(6), 547-561.* | |
| 40 | Appleton, C. C., & Branch, G. M., (1989). Upstream migration by the invasive snail, *Physa-acuta,* in Cape-Town, South-Africa. *South African Journal of Science,* 85(3), 189-190. | |
| 41 | Appleton, C. C., & Miranda, N. A. F., (2015). Two Asian Freshwater Snails Newly Introduced into South Africa and an Analysis of Alien Species Reported to Date. *African Invertebrates,* 56(1), 1-17. | |
| 42 | Appleton, C. C., Donnelly, F. A., & Eriksson, I. M., (1983). The life-cycle and seasonal abundance of *Echinoparyphium-montgomeriana* n-sp (Trematoda, Echinostomatidae) in Natal, South Africa. *South African Journal of Zoology,* 18(4), 320-325. | |
| 43 | Aquilas, N. A., Mukong, A. K., Kimengsi, J. N., & Ngangnchi, F. H., (2022). Economic activities and deforestation in the Congo basin: An environmental kuznets curve framework analysis. *Environmental Challenges,* 8, 100553. | |
| 44 | Arafa, W. M., Hassan, A. I., Snousi, S. A. M., El-Dakhly, Kh. M., Holman, P. J., Craig, T. M., & Aboelhadid, S. M., (2018). Fasciola hepatica infections in cattle and the freshwater snail Galba truncatula from Dakhla Oasis, Egypt. *Journal of Helminthology*, 92(1), 56-63. | |
| 45 | Arostegui, M. C., Wood, C. L., Jones, I. J., Chamberlin, A. J., Jouanard, N., Faye, D. S., Kuris, A. M., Riveau, G., De Leo, G. A., & Sokolow, S. H., (2019). Potential Biological Control of Schistosomiasis by Fishes in the Lower Senegal River Basin. *The American journal of tropical medicine and hygiene*, 100(1), 117-126. | |
| 46 | Assaré, R. K., N'Tamon, R. N., Bellai, L. G., Koffi, J. A., Mathieu, T. B. I., Ouattara, M., Hürlimann, E., Coulibaly, J. T., Diabaté, S., N'Goran, E. K., & Utzinger, J., (2020). Characteristics of persistent hotspots of *Schistosoma mansoni* in western Cote d'Ivoire. *Parasites & Vectors*, 13(1), 337. | |
| 47 | Audibert, M., Josseran, R., & Adjidji, A., (1990). Irrigation, Schistosomiasis, and malaria in the logone valley, Cameroon. *American Journal of Tropical Medicine and Hygiene*, 42(6), 550-560. | |
| 48 | Ayad, N., (1956). Bilharziasis survey in British Somaliland, Eritrea, Ethiopia, Somalia, the Sudan, and Yemen. *The Bulletin of the World Health Organization,* 14(1), 1–117. | |
| 49 | Ayinuola, Y. A., & Adewale, I. O., (2019). Proteomic identification of an alpha class glutathione S-transferase in freshwater snails (Bulinus globosus). *Animal Biology*, 69(3), 377-390. | |
| 50 | Azevedo, J. F., de Medeiros, L . M., & Da Costa Faro, M., (1961). Freshwater mollusks of the Portuguese overseas Provinces. 3, Mollusks of Mozambique. *Estudos Ensaios Documentos Junta Investgacao Ultramar,* 88, 1–394. | |
| 51 | Bacci, G., (1951). Elementi per una malacofauna dell’Abissinia e della Somalia. *Annali del Museo Civico di Storia Naturale di Genova,* 65, 1-144. | |
| 52 | Backeljau, T., Janssens, L., & Joqué, R., (1986) (1985). Report on the freshwater molluscs of the Comoro Islands, collected by the Zoological Mission 1983 of the ‘Koninklijk Museum voor Midden-Afrika, Tervuren’. *Revue de Zoologie Africaine*, 99, 321-330. | |
| 53 | Bagalwa, M., & Baluku, B., (1997). Distribution des mollusques dulcicoles hotes intermediairies des schistosomes humains a Katana, sud-Kivu. Medecine tropicale. *Revue du Corps de Sante Colonial*, 57(4), 369-72. | |
| 54 | Bakhoum, S., Ndione, R. A., & Jason, R. R., (2019). Influence of physicochemical parameters on the spatial distribution of snail species that are intermediate hosts of human schistosomes in the Senegal River Delta. *Medecine et Sante Tropicales,* 29(1), 61-67. | |
| 55 | Baluku, B., Bagalwa, M., & Bisimwa, B., (2000). Parasitologic survey of schistosomiasis due to Schistosoma mansoni in Katana, Democratic Republic of Congo. Medecine Tropicale. *Revue du Corps de Sante Colonial,* 60(2), 163-6. | |
| 56 | Baluku, B., Josens, G., & Loreau, M., 1989. Etude preliminaire de la densite et de la repartition des mollusques dans deux cours d’eau du Zaire oriental. *Journal of African Zoology,* 101, 291-302. | |
| 57 | Bandoni, S. M., Mulvey, M., & Loker, E. S., (1995). Intraspecific and interspecific patterns of allozyme variation among species of Biomphalaria preston, 1910 (Gastropoda: Planorbidae). *Biochemical Systematics and Ecology,* 23 (6), 593-616. | |
| 58 | Barbosa, F. S., Barbosa, I., & Carneiro, E., (1961)a. Notes on morphology and taxonomy of Planorbid snail Biomphalaria pfeifferi (Krauss) from South-East Africa. *Annals of Tropical Medicine and Parasitology,* 55(2), 242-8. | |
| 59 | Barbosa, F. S., Barbosa, I., & Rego, A.M., (1959). Laboratory infection of the snail Planorbis metidjensis (Forbes) from French Morocco with a Brazilian strain of Schistosoma mansoni. *Annals of Tropical Medicine & Parasitology,* 53(3), 314-315. | |
| 60 | Barbosa, F.S., Carneiro, E., & Barbosa, I., (1961)b. On the morphology of Planorbarius metidjensis of Morocco and Portugal and its sensitivity to infection by Schistosoma mansoni. *Annales de Parasitologie Humaine et Comparée,* 36(1/2), 69-75. | |
| 61 | Barré, N., Isautier, H., Frandsen, F., & Mandahl-Barth, G., (1982). Inventaire des mollusques d’eau douce de la Réunion. *Revue d’Elevage et de Médecine vétérinaire des Pays tropicaux,* 35, 35-41. | |
| 62 | Becker, J. M., Ganatra, A. A., Kandie, F., Mühlbauer, L., Ahlheim, J., Brack, W., Torto, B., Agola, E. L.; McOdimba, F., Hollert, H., Fillinger, U., & Liess, M., (2020). Pesticide pollution in freshwater paves the way for *Schistosomiasis* transmission. *Scientific Reports*, 10(1), 1-13. | |
| 63 | Becquet, R., Saout, J., & Pascal, J. M., (1970). La bilharziose intestinale a Schistosoma intercalatum en Republique du Tchad . (A propos de deux observations). *Bulletin de la Societe de Pathologie Exotique,* 63(3), 343-350. | |
| 64 | Belot, J., Geerts, S., & Diouf, M., (1993)a. Observations on the population-dynamics of snail hosts for Schistosomes in the delta of the Senegal river basin. *Journal of Molluscan Studies,* 59:7-13. | |
| 65 | Belot, J., Geerts, S., Sarr, S., & Polderman, A. M., (1993)b. Field trials to control schistosome intermediate hosts by the plant molluscicide Ambrosia maritima L. in the Senegal River Basin. *Acta Tropica*, 52(4), 275-282. | |
| 66 | Bequaert, J. C., & Clench, W. J., (1941). Additions to the rheophilous mollusc fauna of the Congo estuary. *Bulletin of the Museum of Comparative Zoology, Harvard*, 88, 3-13. | |
| 67 | Bequaert, J. C., & de Lucena, D. T., (1951). Introduction into Brazil of 2 species of African snail, *Schistosomiasis* carriers: Bulinus tropicus Krauss and Biomphalaria alexandrina Pfeiffer Krauss. *Revista Brasileira de Medicina*, 8(3), 167-70. | |
| 68 | [Bergquist, R., Zhou, X. N., Rollinson, D., Reinhard-Rupp, J., & Klohe, K., (2017). Elimination of Schistosomiasis: the tools required. *Infectious Disease of Poverty*, 6 (1), 1–9. https://doi.org/10.1186/s40249-017-0370-7](https://doi.org/10.1186/s40249-017-0370-7) | |
| 69 | Berrie, A. D., (1968). Prolonged inhibition of growth in a natural population of freshwater snail Biomphalaria sudanica tanganyicensis (Smith) in Uganda. *Annals of Tropical Medicine and Parasitology*, 62(1), 45-51. | |
| 70 | Betterton, C., (1984)a. Ecological studies on the snail hosts of *schistosomiasis* in the South Chad Irrigation Project Area, Borno State, northern Nigeria. *Journal of Arid Environments,* 7(1), 43-57. | |
| 71 | Betterton, C., (1984)b. Spatiotemporal distributional patterns of *Bulinus-rohlfsi* (clessin), *Bulinus-forskali* (ehrenberg) and *Bulinus-senegalensis* Muller in newly-irrigated areas in Northern Nigeria. *Journal of Molluscan Studies,* 50, 137-152. | |
| 72 | Betterton, C., Fryer, S. E., & Wright, C. A., (1983). *Bulinus-senegalensis* (Mollusca, Planorbidae) in northern Nigeria. *Annals of Tropical Medicine and Parasitology,* 77(2), 143-149. | |
| 73 | Betterton, C., Ndifon, G. T., & Oyeyi, T., (1988)a. *Schistosomiasis* in Kano state, Nigeria .I. Human infections near dam sites and the distribution and habitat preferences of potential snail intermediate hosts. *Annals of Tropical Medicine and Parasitology,* 82(6), 561-570. | |
| 74 | Betterton, C., Ndifon, G. T., & Tan, R. M., (1988)b. *Schistosomiasis* in Kano State, Nigeria. II. Field studies on aestivation in *Bulinus rohlfsi* (Clessin) and *B. globosus* (Morelet) and their susceptibility to local strains of *Schistosoma haematobium* (Bilharz). *Annals of Tropical Medicine and Parasitology,* 82(6), 571–579. | |
| 75 | Binder, E., (1957). Mollusques aquatiques de Côte d’Ivoire. 1. Gasteropodes. *Bulletin de l’Institut Fondamental d’Afrique Noire*, 19, 97-125. | |
| 76 | Binder, E., (1968). Répartition des mollusques dans la Lagune Ebrié (Côte d’Ivoire). *Cahiers de l’Office de la Recherche Scientifique et Technique Outre-Mer, série Hydrobiologie,* 11, 3-34. | |
| 77 | Birgi, E., & Graber, M., (1969). Basommatophore fresh water pulmonate mollusks vectors of livestock parasitic diseases in Chad breeding possibilities in the laboratory. *Revue d'Elevage et de Medecine Veterinaire des Pays Tropicaux*, 22(3), 393-408. | |
| 78 | Bisseru, B., (1953). Some stages in the development of larval echinostomes recovered from molluscs acting as carriers of schistosomes in Central Africa. *Transactions of the Royal Society of Tropical Medicine and Hygiene*, 47(4), 262-263. | |
| 79 | Blackwell, B.A.B., Skinner, A.R., Smith, J.R., Hill, C.L., Churcher, C.S., Kieniewicz, J.M., Adelsberger, K.A., Blickstein, J.I.B., Florentin, J.A., Deely, A.E., & Spillar, K.V., (2017). ESR analyses for herbivore teeth and molluscs from Kharga, Dakhleh, and Bir Tarfawi Oases: Constraining water availability and hominin paleolithic activity in the Western Desert, Egypt. *Journal of African Earth Sciences*, 136, 216-238. | |
| 80 | Bogan, A. E., (2008). Global diversity of freshwater mussels (Mollusca, Bivalvia) in freshwater. *Hydrobiologia*, 595, 139–147. | |
| 81 | Bony, Y. K., Kouassi, N. C.. Diomandé, D., Gourene, G., Verdoit-Jarraya, M., & Pointier, P., (2008). Ecological conditions for spread of the invasive snail Physa marmorata (Pulmonata: Physidae) in the Ivory Coast. *African Zoology*, 43(1), 53-60. | |
| 82 | Bornman, M. S. B., & Bouwman, H., (2012). Environmental Pollutants and Diseases of Sexual Development in Humans and Wildlife in South Africa: Harbingers of Impact on Overall Health? *Reproduction in Domestic Animals,* 47(4), 327-32. | |
| 83 | Bouchet, P., Bary, S., Héros, V., & Marani, G., (2016). How many species of molluscs are there in the world’s oceans, and who is going to describe them? Héros, V., Strong, E., Bouchet, P., *Tropical Deep-Sea Benthos,* 208 (29), Muséum national d'Histoire naturelle, pp.9-24 Mémoires du Muséum national d’Histoire naturelle, 978-2-85653-774-9. ⟨hal-02165705⟩. | |
| 84 | Boulaassafer, K., Ghamizi, M., & Delicado, D., (2018). The genus Mercuria Boeters, 1971 in Morocco: first molecular phylogeny of the genus and description of two new species (Caenogastropoda, Truncatelloidea, Hydrobiidae). *ZooKeys,* 782, 95-128. | |
| 85 | Boulaassafer, K., Ghamizi, M., Machordom, A., & Delicado, D., (2020). Phylogenetic relationships within Pseudamnicola Paulucci, 1878 (Caenogastropoda: Truncatelloidea) indicate two independent dispersal events from different continents to the Balearic Islands. *Systematics & Biodiversity*, 18(4), 396-416. | |
| 86 | Boulaassafer, K., Ghamizi, M., Machordom, A., Albrecht, C., & Delicado, D., (2021). Hidden species diversity of Corrosella Boeters, 1970 (Caenogastropoda: Truncatelloidea) in the Moroccan Atlas reveals the ancient biogeographic link between North Africa and Iberia. *Organisms Diversity & Evolution 21*, 393–420. | |
| 87 | Bourguignat, J. R., (1889). *Mollusques de l’Afrique équatoriale de Moguedouchou à Bagamoyo et de Bagamoyo au Tanganika.* 1-227. Paris, Imprimerie D. Dumoulin et Cie. | |
| 88 | Bourguignat, M. J. R., (1885). *Notice prodromique sur les mollusques terrestres et fluviatiles. Paris:* Savy. | |
| 89 | Bourguignat, M. J. R., (1886). *Nouveautés Malacologiques*: Unionidæ et Iridinidæ du Lac Tanganika. Paris: Savy. | |
| 90 | Bourguignat, M. J. R., (1890). Histoire Malacologique du Lac Tanganika (Afrique Equatoriale). *Annales des sciences naturelles. Zoologie et biologie animale,* 10, 1–267. | |
| 91 | Boxshall, G.A., & Strong, E.E., (2006).An extraordinary shift in life habit within a genus of cyclopid copepods in Lake Tanganyika. *Zoological Journal of the Linnean Society*, 146(2), 275-285. | |
| 92 | Brackenbury, T. D., & Appleton, C. C., (1991). Effect of controlled temperatures on Gametogenesis in the Gastropods *Physa acuta* (Physidae) and *Bulinus-tropicus* (Planorbidae). *Journal of Molluscan Studies*, 57, 461-469. | |
| 93 | Brackenbury, T. D., & Appleton, C. C., (1997). Acute toxicity evaluation of the plant molluscicide, *Apodytes dimidiata* (Icacinaceae), to *Eisenia fetida* (Oligochaeta) and Oreochromis mossambicus (Cichlidae) in South Africa. *Acta Tropica,* 63(1), 1-14. | |
| 94 | Brees, J., Huyse, T., Tumusiime, J., Kagoro-Rugunda, G., Namirembe, D., Mugabi, F., Nyakato, V., Anyolitho, M. K., Tolo, C. U., & Jacobs, L., 2021. The Potential of Citizen-Driven Monitoring of Freshwater Snails in Schistosomiasis Research. Citizen Science: *Theory and Practice*, 6(1), 18, pp. 1–13. https://doi.org/10.5334/cstp.388 | |
| 95 | Brown, D. S., (1965). Freshwater gastropod Mollusca from Ethiopia. *Bulletin of the British Museum (Natural History), Zoology*, 12, 37-94. | |
| 96 | Brown, D. S., (1967). A review of the freshwater Mollusca of Natal and their distribution. *Annals of the Natal Museum*, 18, 477-494. | |
| 97 | Brown, D. S., (1974). A survey of the Mollusca of Lake Chad, Central Africa. Appendix A (Planorbidae and Ancylidae). *Revue de Zoologie Africaine*, 88, 331-343. | |
| 98 | Brown, D. S., (1976). Tetraploid freshwater snail (Planorbidae-bulinus) in highlands of kenya. *Journal of Natural History,* 10(3), 257-267 | |
| 99 | Brown, D. S., (1978). Freshwater molluscs. In *Biogeography and Ecology of Southern Africa,* 2, 1155-1180. Werger, M. J. A. (Ed.). The Hague: W. Junk. | |
| 100 | Brown, D. S., (1980). *Freshwater snails of Africa and their medical importance*. Taylor and Francis, London. | |
| 101 | Brown, D. S., (1983). A fresh-water snail new for Africa - Amerianna-carinata (Planorbidae) found in Nigeria. J*ournal of Molluscan Studies,* 49, 77-79. | |
| 102 | Brown, D. S., (1988). Sierraia - rheophilous west-African river snails (Prosobranchia, Bithyniidae). *Zoological Journal of the Linnean Society*, 93(4), 313-355. | |
| 103 | Brown, D. S., (1991). Fresh-water snails of sao-tome, with special reference to Bulinus-forskalii (ehrenberg), host of Schistosoma-intercalatum. *Hydrobiologia*, 209(2), 141-153. | |
| 104 | Brown, D. S., (1994). Freshwater snails of Africa and their medical importance. Revised 2nd edition, Taylor and Francis, London. | |
| 105 | Brown, D. S., (2001). Taxonomy, biogeography and phylogeny of the non-lacustrine African freshwater snails belonging to the genera Ceratophallus and Afrogyrus (Mollusca: Planorbidae). *Journal of Zoology*, 255, 55-82. | |
| 106 | Brown, D. S., & Curtis, B. A., (1992). Taxonomy of the fresh-water snail *Afrogyrus-anderssoni* (Ancey, 1890, Planorbis) from Namibia (South-West-Africa). *Journal of Molluscan Studies*, 58, 443-446. | |
| 107 | Brown, D. S., & Gerlach, J., (1991). On Paludomus and Cleopatra (Thiaridae) in Africa and the Seychelles Islands. *Journal of Molluscan Studies*, 57:471-479. | |
| 108 | Brown, D. S. & Kristensen, T. K., (1989). *A Field Guide to African Freshwater Snails*. 8. Southern African Species. Charlottenlund: Danish Bilharziasis Laboratory. | |
| 109 | Brown, D. S., & Kristensen, T. K., (1993). *A Field Guide to African Freshwater Snails. 1. West African Species.* Revised 2^nd^ edition. Charlottenlund: Danish Bilharziasis Laboratory. | |
| 110 | Brown, D. S., & Lemma, A., (1970). The molluscan fauna of the awash river Ethiopia in relation to the transmission of Schistosomiasis. *Annals of Tropical Medicine and Parasitology,* 64(4), 533-538. | |
| 111 | Brown, D. S. & Mandahl-Barth, G., (1987). Living molluscs of Lake Tanganyika: a revised and annotated list. *Journal of Concology*, 32, 305-327. | |
| 112 | Brown, D. S., & Rollinson, D., (1996). Aquatic snails of the Bulinus africanus group in Zambia identified according to morphometry and enzymes. *Hydrobiologia,* 324(2), 163-177. | |
| 113 | Brown, D. S., & Shaw, K. M., (1989). Fresh-water snails of the bulinus-truncatus-tropicus complex in kenya - tetraploid species. *Journal of molluscan studies*, 55, 509-532. | |
| 114 | Brown, D. S., & Verdcourt, B., (1998). A new genus and species of freshwater pulmonate (Planorbidae) from Kenya. *Journal of Conchology*, 36, 1-5. | |
| 115 | Brown, D. S., & Wright, C. A. (1978). New species of bulinus (mollusca-gastropoda) from temporary freshwater pools in kenya. *Journal of Natural History,* 12(2), 217-229. | |
| 116 | Brown, D. S., Curtis, B. A., & Appleton, C. C., (1992). Fresh-water snails of East Caprivi and the Lower Okavango River Basin in Namibia and Botswana. *Hydrobiologia,* 246(1), 9-40. | |
| 117 | Brown, D. S., Curtis, B. A., & Rollinson, D., (1996). The freshwater snail Bulinus tropicus (Planorbidae) in Namibia, characterised according to chromosome number, enzymes and morphology. *Hydrobiologia*, 317(2), 127-139. | |
| 118 | Brown, D. S., Curtis, B. A., Bethune, S., & Appleton, C. C., (1992). Freshwater snails of East Caprivi and the lower Okavango River Basin in Namibia and Botswana. *Hydrobiologia*, 246, 9-40. | |
| 119 | Brown, D. S., Fison, T., & Wright, C. A., (1984). Aquatic snails of the Jonglei region, Southern Sudan, and transmission of trematode parasites. *Hydrobiologia*, 110, 247-271. | |
| 120 | Brown, D. S., Matovu, D. B., & Rollinson, D., (1982). Bulinus coulboisi of Lake Tanganyika: assessment of its taxonomic position and role as intermediate host for S. haematobium , J*ournal of Natural History*, 16:5, 673-687, DOI: 10.1080/00222938200770511 | |
| 121 | Brown, D. S., Shaw, K. M., & Rollinson, D., (1986). Bulinus-guernei (Mollusca, Gastropoda) of west-Africa - taxonomic status and role as host for Schistosomes. *Zoological journal of the linnean society*, 88(1), 59-90. | |
| 122 | Brown, D. S., Shaw, K. M., & Rollinson, D., (1991). Fresh-water snails of the Bulinus truncatus tropicus complex (Basommatophora, Planorbidae) in Kenya - diploid populations. *Journal of molluscan studies,* 57, 143-166. | |
| 123 | Brown, D. S., Fiston, T., Southgate, V. R., & Wright, C. A., (1984). Aquatic snails of the Jonglei region, southern Sudan, and transmission of trematode parasites. *Hydrobiologia*, 110, 247-271. | |
| 124 | Brown, D. S.., Jelnes, J. E., Kinoti, G. K., & Ouma, J., (1981). Distribution in Kenya of intermediate hosts for *Schistosoma*. *Tropical and Geographical Medicine*, 33, 95-103. | |
| 125 | Bruijning, C. F., (1969). Bilharziasis in irrigation schemes in Ethiopia. *Tropical and geographical medicine*, 21(3),280. | |
| 126 | Burch, J. B., Bruce, J. I., Rudolph, P. H., Mallett, J. C., & Banhawy, M. A. (1979). Chromosome-numbers of 4 Populations of Bulinine snails from Lake Nasser, Egypt. *Tropenmedizin und Parasitologie*, 30(2), 174-178. | |
| 127 | Busch, J., Soreghan, M., de Beurs, K., McGlue, M., Kimirei, I., Cohen, A., & Ryan, E., (2018). Linking watershed disturbance with nearshore sedimentation and the shell beds of Lake Tanganyika (Mahale Mountains, Tanzania). Environmental Earth Sciences 77(13). DOI10.1007/s12665-018-7644-7. | |
| 128 | Campagne, G., Poda, J. N., & Chippaux, J. P., (1998). Schistosomiasis risk in the region of the dam of Bagre, Burkina Faso. Medecine Tropicale. *Revue du Corps de Sante Colonial*, 58(4), 415-6. | |
| 129 | Campbell, S. J., Stothard, J. R., O'Halloran, F., Sankey, D., Durant, T., Ombede, D. E., Chuinteu, G. D., Webster, B. L., Cunningham, L., LaCourse, E. J., & Tchuem-Tchuente, L. A., (2017). Urogenital schistosomiasis and soiltransmitted helminthiasis (STH) in Cameroon: An epidemiological update at Barombi Mbo and Barombi Kotto crater lakes assessing prospects for intensified control interventions. *Infectious Disease of Poverty* 6, 49. | |
| 130 | Cantrell, M. A., (1981). Bilharzia Snails and Water Level Fluctuations in a Tropical Swamp. *Oikos,* 6(2), 226-232. | |
| 131 | Capron, A., & Brygoo, E. R., (1959). Intestinal schistosomosis due to Schistosoma mansoni on Madagascar. Study of the focus at Ambositra. I. Malacologic and parasitologic investigation. *Bulletin de la Société de Pathologie Exotique*, 52(4), 503-515. | |
| 132 | Capron, A., S. Deblock, S., Biguet, J., A. Clay, A., Adenis, L., & Vernes, A., (1965). Contribution à l'étude expérimentale de la bilharziose à Schistosoma haematobium. *Bulletin of the World Health Organization,* 32(6), 755–778. | |
| 133 | [Carteret, X., (2012). Michel Adanson in Senegal (1749-1754): A Great Naturalist and Anthropological Journey of the Enlightenment. *Revue d’Histoire des Sciences*, 65(1), 5-25. https://doi.org/10.3917/rhs.651.0005](https://doi.org/10.3917/rhs.651.0005) | |
| 134 | Casaca, V. R., & Carvalho, A. M. de., (1955). ProspecYao das endemias reinantes na area de Vila Salazar (Dalatando-Angola). *Anais do Instituto de Medicina Tropica Lisboa,* 12, 575-591. | |
| 135 | Casagranda, C., & Boudouresque, C. F., (2002). A sieving method for rapid determination of size-frequency distribution of small gastropods. Example of the mud snail Hydrobia ventrosa (Gastropoda: Prosobranchia). *Hydrobiologia,* 485, 143–152. | |
| 136 | Catalano, S., Léger, E., Fall, C. B., Borlase, A., Diop, S. D., Berger, D., Webster, B. L., Faye, B., Diouf, N. D., Rollinson, D., Sène, M., Bâ, K., & Webster, J. P., (2020). Multihost Transmission of Schistosoma mansoni in Senegal, 2015-2018. *Emerging Infectious Diseases*, 26(6), 1234-1242. | |
| 137 | Catharina, C., Geertz, T., Rassam, H., Hailegebriel, T., Albrecht, C., (2022). Freshwater diversity at a biogeographic edge zone: the high-mountain pea-clams of Ethiopia. *Systematics and Biodiversity Systematics and Biodiversity*, 20(1),1-15. https://doi:10.1080/14772000.2021.2005706 | |
| 138 | Cawston, F. G., (1946). Schistosomiasis in Southern Africa in its relation to the pollution of river-pools. *SA Medicinal Journal*, 20(9), 240-241. | |
| 139 | Cawston, F. G., (1947). Schistosomiasis in southern Africa and its relation to rainfall, artificial methods of control and the natural enemies of the Molluscan hosts. *South African Journal of Science*, 43(7), 204-209. | |
| 140 | Centers for Disease Control (CDC), (1982). Cercarial dermatitis among bathers in California; katayama syndrome among travelers to Ethiopia. MMWR. *Morbidity and mortality weekly report,*31(32), 435-8. | |
| 141 | Cetron, M. S., Chitsulo, L., Sullivan, J. J., Pilcher, J., Wilson, M., Noh, J., Tsang, V. C., Hightower, A. W., & Addiss, D. G., (1996). Schistosomiasis in Lake Malawi. *Lancet*, 348(9037), 1274-1278. | |
| 142 | Chaouti, A., Azirar, A., & Bayed, A., (2019). Macrofaunal spatial distribution and community structure in a lagoon without a river discharge (the Oualidia lagoon, NW Morocco). *Marine Ecology*, 40(4). | |
| 143 | Chartier, C., Cabaret, J., & Kristensen, T. K., (1993). Environmental and geographical-distribution in north-east Zaire of intermediate hosts of bovine Fascioliasis (Lymnaea-natalensis) and *Schistosomiasis (Bulinus-africanus*). J*ournal of Molluscan Studies,* 59, 117-119. | |
| 144 | Chartier, C., Ngota, A., & Cabaret, J., (1990). Dynamics of Lymnaea-natalensis populations in the Bunia area (Ituri, Haut-Zaire). *Annales de Parasitologie Humaine et Comparee,* 65(4), 177-182. | |
| 145 | Chimbari, M. J., (2012). Enhancing Schistosomiasis Control Strategy for Zimbabwe: Building on Past Experiences. *Journal of Parasitology Research,* 353768. https://doi:10.1155/2012/35376 | |
| 146 | Chimbari, M. J., Madsen, H., & Ndamba, J., (1997). Laboratory experiments *Sargochromis codringtoni*, a candidate for biological control of the schistosomiasis on snail predation by snails that transmit. *Annals of Tropical Medicine & Parasitology*, 91(1), 95-102. | |
| 147 | Chimbari, M. J., Kalinda, C., & Siziba, N., (2020). Changing patterns of Schistosoma host snail population densities in Maun, Botswana. A*frican Journal of Aquatic Science*, 45(4), 493-499. | |
| 148 | Chibwana, F. D., Tumwebaze, T., Mahulu, A., Sands, A. F., &Albrecht, C., (2020). Assessing the diversity and distribution of potential intermediate hosts snails for urogenital schistosomiasis: Bulinus spp. (Gastropoda: Planorbidae) of Lake Victoria. Parasites Vectors, 13, 418. https://doi.org/10.1186/s13071-020-04281-1. | |
| 149 | Chingwena, G., Mukaratirwa, S., Kristensen, T. K., & Chimbari, M., (2002). Larval trematode infections in freshwater snails from the highveld and lowveld areas of Zimbabwe. *Journal of Helminthology*, 76(4), 283-93. | |
| 150 | Chingwena, G., Mukaratirwa, S., Chimbari, M., Kristensen, T. K., & Madsen, H., (2004). Population dynamics and ecology of freshwater gastropods in the highveld and lowveld regions of Zimbabwe, with emphasis on schistosome and amphistome intermediate hosts. *African Zoology*, 39(1), 55-62. | |
| 151 | Chlyeh, G., Dodet, M., Delay, B., Khallaayoune, K., & Jarne, P., (2006). Spatio-Temporal Distribution of Freshwater Snail Species in Relation to Migration and Environmental Factors in an Irrigated Area from Morocco. *Hydrobiologia*, 553(1), 129-142. | |
| 152 | Chlyeh, G., Henry, P. Y., & Jarne, P., (2003). Spatial and temporal variation of life-history traits documented using capture-mark-recapture methods in the vector snail Bulinus truncatus. *Parasitology*, 127(3), 243-51. | |
| 153 | Chlyeh, G., Henry, P.Y., Sourrouille, P., Delay, B., Khallaayoune, K., & Jarne, P., (2002). Population genetics and dynamics at short spatial scale in Bulinus truncatus, the intermediate host of Schistosoma haematobium, in Morocco. *Parasitology,* 125(4), 349-57. | |
| 154 | Choudhry, A.W., (1975). Results of 5 years of snail control at a hero pilot scheme, Kenya. *East African Medical Journal,* 52(10), 573-577. | |
| 155 | Chougar, L., Mas‐Coma, S., Artigas, P., Harhoura, K., Aissi, M., Agramunt, V.H., & Bargues, M.D., (2020). Genetically 'pure' Fasciola gigantica discovered in Algeria: DNA multimarker characterization, trans‐Saharan introduction from a Sahel origin and spreading risk into north‐western Maghreb countries. *Transboundary & Emerging Diseases*, 67 (5), 2190-2205. | |
| 156 | Chu, K. Y., (1978). Trials of ecological and chemical measures for control of Schistosoma-haematobium transmission in a Volta lake village. *Bulletin of the World Health Organisation*, 56(2), 313-322. | |
| 157 | Chu, K. Y., Vanderburg, J. A., & Klumpp, R. K., (1981). Transmission dynamics of miracidia of S*chistosoma haematobium* in the Volta Lake. *Bulletin of the World Health Organisation,* 59(4), 555–560. | |
| 158 | Chu, K.Y., & Vanderburg, J.A., (1976). Techniques for estimating densities of *Bulinus truncatus* rohlfsi and its horizontal distribution in Volta Lake, Ghana. *Bulletin of the World Health Organization,* 54(4), 411–416. | |
| 159 | Churcher, C. S., Kleindienst, M. R., & Schwarcz, H. P., (1999). Faunal remains from a Middle Pleistocene lacustrine marl in Dakhleh Oasis, Egypt: palaeoenvironmental reconstructions. *Palaeogeography, Palaeoclimatology, Palaeoecology,* 154(4), 301-312. | |
| 160 | Cisse, F., Diallo, S., & Dieng, Y., (1983). Bilan actuel de la bilharziose urinaire chez les populations riveraines du lac de Guiers (nord du Senegal). *Dakar Medical,* 28(2), 343-50. | |
| 161 | Clennon, J.A., (2006). *Eco -epidemiology of Schistosoma haematobium:* Spatial and temporal heterogeneity of infection and snail dispersal in Msambweni, Kenya. University of Illinois at Urbana-Champaign. | |
| 162 | Cohen, A. S., Talbot, M. R., Awramik, S. M., Dettman, D. L., & Abell, P., (1997). Lake level and paleoenvironmental history of Lake Tanganyika, Africa, as inferred from late Holocene and modern stromatolites. *Geological Society of America Bulletin*, 109(4), 444-460. | |
| 163 | Colette, J., Sellin, B., & Simonkovich, F., (1982). Etude epidemiologique de la substitution de Schistosoma haematobium par Schistosoma mansoni dans une zone d'endemie bilharzienne d'Afrique de l'ouest (Haute-Volta). *Medecine Tropicale*, 42(3), 289-296. | |
| 164 | Connolly, M., (1912). A revised reference list of South African non-marine Mollusca; with descriptions of new species in the South African Museum. *Annals of the South African Museum,* 11, 59–306. | |
| 165 | Connolly, M., (1925). The non-marine Mollusca of Portuguese East Africa. *Transactions of the Royal Society of South Africa,* 12, 105-220. | |
| 166 | Connolly, M., (1939). A monographic survey of the South African non-marine Mollusca. *Annals of the South African Museum*, 33, 1-660. | |
| 167 | Cooke, G. S., Lalvani, A., Gleeson, F. V., & Conlon, C. P., (1999). Acute pulmonary schistosomiasis in travelers returning from Lake Malawi, sub-Saharan Africa. *Clinical Infectious Diseases*, 29(4), 836-839. | |
| 168 | Coumbaras, A., (1966). Hepatic distomatosis in Algeria. A*nnales de Parasitologie Humaine et Comparée,* 41(1), 71-77. | |
| 169 | Courtois, C. M., & Gebert, F., (1979). Recent observations on Schistosomiasis in Mauritius. T*ropical and Geographical Medicine*, 31(3), 381-387. | |
| 170 | Covich, A. P., Palmer, M. A., & Crowl, T. A., (1999). The role in of species invertebrate freshwater ecosystems—Zoobenthic species influence energy flows and nutrient cycling. *Bioscience*, 49, 119–127. | |
| 171 | Cowper, A. G., (1947). Observations on the life-cycle of Schistosoma-mansoni in the laboratory, with a discussion on the snail vectors of S-mansoni and S-haematobium. *Annals of Tropical Medicine and Parasitology*, 41(2), 173-175. | |
| 172 | Cowper, S. G., (1954). On African *Schistosomiasis*. *Transactions of the Royal Society of Tropical Medicine and Hygiene*, 48(2), 185-186. | |
| 173 | Cowper, S. G., (1959). Notes on the snail vector of *Schistosoma haematobium* In the Ibiidan area of Nigeria, with some observations on the pathology of the liver and spleen in mice experimentally infected with *Schistosoma haematobium* and *Schistosoma mansoni*. *West African Medical Journal*, NS 8(6), 191-196. | |
| 174 | Cridland, C. C., (1970). Susceptibility of the snail Biomphalaria alexandrina alexandrina from the UAR and the Sudan to infection with a strain of Schistosoma mansoni from Tanzania. *Bulletin of the World Health Organization*, 43(6), 809–815. | |
| 175 | Cridland, C. C., (2018). Further experimental infection of several species of East African freshwater snails with *Schistosoma mansoni* and *S. haematobium.* *Journal of Tropical Medicine and Hygiene*, 60(1), 18-23. | |
| 176 | Cruz-Rivera, E., & Malaquias, M. A. E., (2016). Ecosystem Alterations and Species Range Shifts: An Atlantic-Mediterranean Cephalaspidean Gastropod in an Inland Egyptian Lake. *PlosOne,* 11(6), e0156760. | |
| 177 | Cumberlidge, N., Rollinson, D., Vercruysse, J., Louis-Albert, T. T., Webster, B., & Clark, P. F., (2018). Paragonimus and paragonimiasis in West and Central Africa: unresolved questions. *Parasitology*, 145(13), 1748-1757. | |
| 178 | Dabo, A., Diop, S., & Doumbo, O., (1994). Snail intermediate hosts distribution in the human Schistosomiasis in the office-du-Niger (Mali) .2. role of different habitats in the transmission. *Bulletin de la societe de pathologie exotique,* 87(3), 164-169. | |
| 179 | [Dai, A., Qian, T., Trenberth, K. E., & Milliman, J. D., (2009). Changes in continental freshwater discharge from 1948 to 2004. *Journal of Climate*, 22(10), 2773–2792. https://doi.org/10.1175/2008J CLI25 92.1](https://doi.org/10.1175/2008J%20CLI25%2092.1) | |
| 180 | Dallimer, M., & Melo, M., (2010). Rapid decline of the endemic giant land snail Archachatina bicarinata on the island of Príncipe, Gulf of Guinea. *Oryx,* 44(2), 213-218. | |
| 181 | Dalu, T., & Wasserman, R. J., (Editors) (2022). *Tropical freshwater wetlands:* From ecology to conservation management (Cambridge: Elsevier). | |
| 182 | Dana, P., & Appleton, C. C., (2007). Observations on the population dynamics of the invasive freshwater snail Aplexa marmorata (Pulmonata: Physidae) in Durban, South Africa. *South African Journal of Science*, 103(11/12), 493-496. | |
| 183 | Danaher, C., Newbold, T., Cardille, J., & Chapman, A. S. A., (2022). Prioritizing conservation in sub-Saharan African lakes based on freshwater biodiversity and algal bloom metrics. *Conservation Biology*, e13914. https://doi.org/10.1111/cobi.13914 | |
| 184 | Danish Bilharziasis Laboratory, (1977). *A Field Guide to African Freshwater Snails. 4. South East African Species*. Charlottenlund: Danish Bilharziasis Laboratory. | |
| 185 | Danish Bilharziasis Laboratory, (1982). *Guide de Terrain des Gasteropodes d’Eau douce Africans. 5. Afrique centrale.* Charlottenlund: Danish Bilharziasis Laboratory. | |
| 186 | Danish Bilharziasis Laboratory, (1983). *A Field Guide to Freshwater Snails in Countries of the WHO Eastern Mediterranean Region*. Copenhagen: Danish Bilharziasis Laboratory. | |
| 187 | Danish Bilharziasis Laboratory, (1986). *A Field Guide to African Freshwater Snails. 3. North East African Species*. 2^nd^ edition. Charlottenlund: Danish Bilharziasis Laboratory. | |
| 188 | Danish Bilharziasis Laboratory, (1987). *A Field Guide to African Freshwater Snails. 2. East African Species.* 2^nd^ edition. Charlottenlund: Danish Bilharziasis Laboratory. | |
| 189 | Darwall, W., Smith, K., Allen, D., Holland, R., Harrison, I., & Brooks, E. (Eds). (2011). *The diversity of life in African freshwaters*: Underwater, under threat: An analysis of the status and distribution of freshwater species throughout mainland Africa. Gland, Switzerland: IUCN. | |
| 190 | Davadee, J.A., & Metge, R., (1965). Regression of a focus of schistosomosis in the Sahara. *Bulletin de la Societe de Pathologie Exotique*, 58(1), 81-88. | |
| 191 | Davis, S. D., Heywood, V. H., & Hamilton, A. C., (1994). *Centres of Plant Diversity*. A Guide and Strategy for Their Conservation. Volume 1: Europe, Africa, South West Asia and the Middle East. WWF/IUCN, Oxford. | |
| 192 | De Azevedo, J. F., Colaco, A. T., & Faro, M. M., (1954)a. Human bilharziasis in the South of Save (Mozambique). *Anais do Instituto de Medicina Tropical,*11(1), 5-120. | |
| 193 | De Azevedo, J. F., Da Costa, M. M., & Gomes, F. A., (1954)b. Susceptibility of Planorbis metidjensis to Schistosoma haematobium of Portuguese Guinea and to Schistosoma mansoni of Mozambique. *Anais do Instituto de Medicina Tropical,*11(2), 251-60. | |
| 194 | De Bont, A. F., & De Bont Hers, M. J., (1952). Mollusc Control and Fish-Farming in Central Africa. *Nature,* 170, 323–324. | |
| 195 | De Carvalho, A.C., Janz, G.J., & Mexia, J.T., (1966). Subsidies for studying and identifying the intermediate hosts of human schistosomes in Angola. II. Biomphalaria. (With data on the distribution of mansoni bilharziasis in Angola. Anais do Instituto de Medicina Tropical 23(1), 59-98. | |
| 196 | De Kock, K. N., Pretorius, S. J., & Van Eeden, J. A., (1974). Voorlopige kommentaar aangaande die vorkomens van die varswaterslakke in die Oranjerivier. 1. Die opvanggebied, 2. Die Hendrik Verwoerd Dam. In The *Orange River*, 187-212. E.M. Van Zinderen Bakker (Ed.). Bloemfontein, University of The Orange Free State. | |
| 197 | De Kock, K. N., & Wolmarans, C. T., (2005)a. Distribution and habitats of *Bulinus depressus* and possible role as intermediate host of economically important helminth parasites in South Africa. *Water SA,* 31(4), 491-496. | |
| 198 | De Kock, K. N., & Wolmarans, C. T., (2005)b. Distribution and habitats of the *Bulinus africanus* species group, snail intermediate hosts of *Schistosoma haematobium* and *Schistosoma mattheei* in South Africa. *Water SA,* 31(1), 117-125. | |
| 199 | De Kock, K. N., & Wolmarans, C. T., (2005)c. Distribution, habitats and role as intermediate host of the freshwater snail, *Bulinus forskalii*, in South Africa. *Onderstepoort Journal of Veterinary Research,* 72(2), 165-174. | |
| 200 | De Kock, K. N., & Wolmarans, C. T., (2007)a. Distribution and habitats of *Corbicula fluminalis africana* (Mollusca: Bivalvia) in South Africa. *Water SA,* 33(5), 709-715. | |
| 201 | De Kock, K. N., & Wolmarans, C. T., (2007)b. Distribution and habitats of the alien invader freshwater snail *Physa acuta* in South Africa. *Water SA,* 33(5), 717-722. | |
| 202 | De Kock, K. N., & Wolmarans, C. T., (2008). Invasive alien freshwater snail species in the Kruger National Park, South Africa. *Koedoe,* 50(1), 126. | |
| 203 | De Kock, K. N., & Wolmarans, C. T., (2009). Distribution and habitats of *Melanoides tuberculata* (Muller, 1774) and *M. victoriae* (Dohrn, 1865) (Mollusca: Prosobranchia: Thiaridae) in South Africa. *Water SA,* 35(5), 713-720. | |
| 204 | De Kock, K. N., & Wolmarans, C. T., (2017). Distribution and habitats of *Burnupia trapezoidea* (Boettger, 1910) (Gastropoda: Ancylidae) in South Africa. *Water SA,* 43(2), 258-263. | |
| 205 | De Kock, K. N., Wolmarans, C. T., & Bornman, M., (2003). Distribution and habitats of the snail *Lymnaea truncatula*, intermediate host of the liver fluke *Fasciola hepatica*, in South Africa. *Journal of the South African Veterinary Association,* 74(4), 117-122. | |
| 206 | De Kock, K. N., Wolmarans, C. T., & Bornman, M., (2004). Distribution and habitats of *Biomphalaria pfeifferi*, snail intermediate host of *Schistosoma mansoni*, in South Africa. *Water SA,* 30(1), 29-36 | |
| 207 | De Kock, K.N., Joubert, P.H., & Pretorius, S.J., (1989). Geographical-distribution and habitat preferences of the invader fresh-water snail species Lymnaea-columella (Mollusca, Gastropoda) in South-Africa. *Onderstepoort Journal of Veterinary Research*, 56(4), 271-275. | |
| 208 | De Meillon, B., Frank, G. H., & Allanson, B. R., (1958). Some aspects of snail ecology in South Africa. A preliminary report. *Bulletin of the World health Organisation*, 18, 771-783. | |
| 209 | De Morais, T., (1956). Study of mollusc vectors of bilharziasis in Zambesia (Mozambique). *Anais do Instituto de Medicina Tropical*, 13(4), 883-99. | |
| 210 | Declercq, D. D., (1987). The malacological situation in Kinshasa and description of an autochthonous *Schistosomiasis* intercalatum focus. *Annales de la Societe Belge de Medecine Tropicale*, 67(4), 345-352. | |
| 211 | Declercq, D., Henry, M. C., & Hubert, P., (1985). Survey of a Schistosomiasis-mansoni focus in Mayombe, Zaire. *Annales de la Societe Belge de Medecine Tropicale*, 65(2), 153-162. | |
| 212 | Degner, E., (1934). Westafrikanische Landschnecken. I. Streptaxiden, Helicarioniden, | |
| 213 | Degrémont, A. A., (1973). *Mangoky Project Campaign against Schistosomiasis in the Lower Mangoky (Madagascar).* Basle, Swiss Tropical Institute. | |
| 214 | Degremont, A. A., Pedrazzi, M., & Real, A., (1970). Note preliminaire sur l'hemolyse des hematies de lapin par l'hepato-pancreas de *Bulunus liratus* et son application dans la taxonomie des *Bulinus* de Madagascar. *Acta tropica*, 27, 266-267. | |
| 215 | DeJong, R. J., (2003). Phylogeography and genetic diversity of the human parasite *Schistosoma mansoni* and its snail host *Biomphalaria* Phylogeography and genetic diversity of the human parasite *Schistosoma mansoni* and its snail host *Biomphalaria*. The University of New Mexico. | |
| 216 | DeJong, R. J., Morgan, J. A. T., Paraense, W. L., Pointier, J-P., Amarista, M., Ayeh-Kumi, P. F. K., Babiker, A., Barbosa, C. S., Brémond, P., Canese, A. P., Pereira de Souza, C., Dominguez, C., File, S., Gutierrez, A., Incani, R. N., Kawano, T., Kazibwe, F., Kpiokpi, J., Lwambo, N. J. S., Mimpoundi, R., Njiokou, F., Poda, J. N., Sene, M., Velásquez, L. E., Yong, M., Adema, C. M., Hofkin, B. V., Mkoji, G. M. & Loker, E. S., (2001). Evolutionary Relationships and Biogeography of Biomphalaria (Gastropoda: Planorbidae) with Implications Regarding Its Role as Host of the Human Bloodfluke, *Schistosoma mansoni. Molecular Biology and Evolution,* 18(12), 2225-2239. | |
| 217 | Dennis, E., Vorkpor, P., & Degremont, A., (1983). Studies on the epidemiology of schistosomiasis in Liberia - the prevalence and intensity of Schistosomal infections in bong county and the bionomics of the snail intermediate hosts. *Acta tropica,* 40(3), 205-229. | |
| 218 | Deribew, K., Erko, B., Mereta, S. T., Yewhalaw, D., & Mekonnen, Z., (2022). Assessing Potential Intermediate Host Snails of Urogenital Schistosomiasis, Human Water Contact Behavior and Water Physico-chemical Characteristics in Alwero Dam Reservoir, Ethiopia. *Environmental Health Insights*, 16(1). | |
| 219 | Deschiens, R., & Le Corroller, Y., (1966). La repartition des gites de Bulinus contortus, vecteurs de la bilharziose urinaire au Maroc. *Bulletin de la Societe de Pathologie Exotique*, 58, 455-461. | |
| 220 | Deschiens, R., & Poirier, A., (1967). Epidemiologic and clinical aspects of Schistosomiasis due to Schistosoma-intercalatum in Gabon human Bulinus pyrogophysa-forsakalii nitro thiami dazole anti parasit bilharstan anti parasit. *Bulletin de la Societe de Pathologie Exotique*, 60(3), 228-240. | |
| 221 | Deschiens, R., Dechance, M., & Vermeil, C., (1955). Predatory action of fresh-water crabs of the genus Potamon on the mollusc vectors of schistosomiasis. *Bulletin de la Societe de Pathologie Exotique,* 48(2), 203-207. | |
| 222 | Diakité, N. R., N'Zi, K. G., Ouattara, M., Coulibaly, J. T., Saric, J., Yao, P. K., Hattendorf, J., Utzinger, J., & N'Goran, E. K., (2018). Association of riverine prawns and intermediate host snails and correlation with human schistosomiasis in two river systems in south-eastern Côte d'Ivoire. *Parasitology*, 145(13), 1792-1800. | |
| 223 | Diakite, N. R., Winkler, M. S., Coulibaly, J. T., Guindo-Coulibaly, N., Utzinger, J., & N'Goran, E. K., (2017). Dynamics of freshwater snails and Schistosoma infection prevalence in schoolchildren during the construction and operation of a multipurpose dam in central Cote d'Ivoire. *Infectious Disease of Poverty*, 6,93. | |
| 224 | Digenea, C. T. L. O., (1972). Compatibility and host parasite relationships between species of the genus Bulinus basommatophora Planorbidae and an egyptian strain of Schistosoma-haematobium trematoda. *Malacologia*, 11(2), 225-280. | |
| 225 | Din Sharaf, E. l., & Nagar, H. E. I., (1955). Control of snails by copper sulphate in the canals of the Gezira irrigated area of the Sudan. *Journal of Tropical Medicine and Hygiene*, 58(11), 260-263. | |
| 226 | Dinnik, J. A., & Dinnik, N. N., (1963). Effect of the seasonal variations of temperature on the development of *Fasciola gigantica* in the snail host in the Kenya Highlands. *Bulletin of Epizootic Diseases of Africa,* 11(2), 197-207. | |
| 227 | Dinnik, J. A., (1961). Paramphistomum phillerouxi sp. nov. (Trematoda: Paramphistomatidae) and its development in *Bulinus forskalii*. *Journal of Helminthology*, 35, 69-90. | |
| 228 | Dirisu, A. R., & Surtasi, E. I., (2021). Bioindicators of lotic and lentic ecosystems in Agbede wetlands (Southern Nigeria), using macroinvertebrate tools. *Scientific African,* 14, e01000. | |
| 229 | Dobson, M., (2004). Replacement of native freshwater snails by the exotic Physa acuta (Gastropoda: Physidae) in southern Mozambique; a possible control mechanism for schistosomiasis. *Annals of Tropical Medicine & Parasitology*, 98(5), 543-548. | |
| 230 | Dogba, K.M., & Jelnes, J.E., (1985). Preliminary observations on geographical variation in enzymes of African intermediate hosts of schistosomes: the genera *Bulinus and Biomphalaria* (Gastropoda: Planorbidae) from Togo. *Hereditas*, 103(2), 231-233. | |
| 231 | Dohrn, H., (1864). List of shells collected by Capt. Speke during his second journey through Central Africa. *Proceedings of the Zoological Society of London*, 1864, 116-118. | |
| 232 | Dohrn, H., (1865). List of the land and freshwater shells of the Zambezi and Lake Nyasa, eastern tropical Africa, collected by John Kirk. *Proceedings of the Zoological Society of London*, 1865, 231-234. | |
| 233 | Donald, B., & Mc Mullen, J. F., (1962). Report on a preliminary survey by the WHO *Bilharziasis* Advisory Team in Upper Volta. *The Bulletin of the World Health Organization*, 27(1), 5–24. | |
| 234 | Donnelly, F. A., & Appleton, C. C., (1985). Observations on the field transmission dynamics of Schistosoma-mansoni and Schistosoma-mattheei in Southern Natal, South-Africa. *Parasitology*, 91, 281-290. | |
| 235 | Donohue, I., & Irvine, K., (2004). Size-specific effects of increased sediment loads on gastropod communities in Lake Tanganyika, Africa. *Hydrobiologia*, 522(1-2), 337-342. | |
| 236 | Doumbo, O., Dabo, A., & Quilici, M., (1992). Epidemiology of human urban schistosomiasis in Bamako in Mali (the case of the "populous" quarter of Bankoni). Medecine tropicale. *Revue du Corps de sante colonial*, 52(4), 427-34. | |
| 237 | Doums, C., & Jarne, P., (1996). The evolution of phally polymorphism in *Bulinus truncatus* (Gastropoda, Planorbidae): The cost of male function analysed through life-history traits and sex allocation. *Oecologia*, 106(4), 464-469. | |
| 238 | Doums, C., Bremond, P., & Jarne, P., (1996). The genetical and environmental determination of phally polymorphism in the freshwater snail *Bulinus truncatus*. *Genetics,* 142 (1), 217-225. | |
| 239 | Doums, C., Viard, F., David, P., & Jarne, P., (1997). Phally status and size in Niger populations of Bulinus truncatus (Gastropoda: Planorbidae). *Journal of Molluscan Studies,* 63(1), 111-115. | |
| 240 | Dudgeon, D., Arthington, A. H., Gessner, M. O., Kawabata, Z. I., Knowler, D. J., Lévêque, C., & Sullivan, C. A., (2006). Freshwater biodiversity: Importance, threats, status and conservation challenges. *Biological Reviews*, 81(2), 163–182. | |
| 241 | Duke, B. D., & Moore, P. J., (1976). Use of a molluscicide, in conjunction with chemotherapy, to control Schistosoma haematobium at Barombi Lake foci in Cameroon .1. attack on snail hosts, using n-tritylmorpholine, and effect on transmission from snail to man. *Tropenmedizin und Parasitologie,* 27(3), 297-313. | |
| 242 | Duncan, J., & Lemma, A., (1976). Investigations into the control of schistosomiasis at the HVA Wonji-Shoa sugar estates in Ethiopia. 1. Initiation of the project. *Ethiopian medical journal*, 14(1), 3-15. | |
| 243 | Dunker, G., (1853). Index Molluscorum quae in Itinere ad Guineam Inferiorum Collegit Georgius Tams. Cassel. | |
| 244 | Dupouy, J., Abdelhak, F., & Yazid, F., (1980)a. Competition interspecifique entre Melanopsis praemorsa L. (Prosobranchia: Thiaridae) et certains basommatophores en Oranie et au Sahara nord-occidentale. *Journal of Molluscan Studies*, 46, 1-12. | |
| 245 | Dupouy, J., Abdelhak, F., & Yazid, F., (1980)b. Interspecific competition between *Melanopsis-praemorsa* l (Prosobranchiao, Thiaridae) and certain Basommatophora in Oran and Northwestern Sahara - Prospect of application to fight against *Schistosomiasis. Journal of Molluscan Studies,* 46, 1-12. | |
| 246 | EI-Gindy, M. S., (1957). Distribution and ecology of the snail vectors of Schistosomiasis in Egypt. *Journal of the Egyptian Medical Association*, 40, 192-204. | |
| 247 | El Gamal, Abd El-Rahman A., Ismail, & Nahed M. M., (2005). Food composition and feeding habits of some fresh water fishes in various water systems at Abbassa, Egypt, with special reference to snails transmitting diseases. *Journal of the Egyptian Society of Parasitology,* 35(2), 637-52. | |
| 248 | El Kholy, H., Siongok, T. K. A., Koech, D., Sturrock, R. F., Houser, H., King, C. H., & Mahmoud, A. A., (1989). Effects of borehole wells on water utilization in schistosoma-haematobium endemic communities in coast-province, Kenya. *American Journal of tropical Medicine and Hygiene*, 41(2), 212-219. | |
| 249 | El-Assal, F. M., Shoukry, N. M., & Mansour, N. S., (1997). Infection of laboratory bred Biomphalaria alexandrina from Giza and Alexandria Governorates with Schistosoma mansoni from Giza in relation to snail size and number of penetrated miracidia. *Journal of the Egyptian Society of Parasitology*, 27(3), 739-754. | |
| 250 | Eldblom, C., & Kristensen, T. K., (2003). A revision of the genus Melanoides (Gastropoda:Thiaridae) in Lake Malawi. *African Zoology,* 38(2), 357-369. | |
| 251 | El-Guindy, H. I., (1969). Copper sulfate as a molluscicide against Bulinus-truncatus eggs in Iraq. *Journal of the Egyptian Medical Association,* 52(4), 245-257. | |
| 252 | El-Khayat, H. M. M., Mahmoud, K. M. A., & Abu Taleb, H. M., (2011). Habitat characteristics for different freshwater snail species as determined biologically through macroinvertebrate information. *Journal of the Egyptian Society of Parasitology*, 41(3), 651-664. | |
| 253 | El-Khayat, H. M. M., Mossalem, H. S., El-Hommossany, K., Sayed, S. S. M., Mohammed,W. A., Zayed, K. M., Saied, M., & Habib, M. R., (2022). Assessment of schistosomiasis transmission in the River Nile at Greater Cairo using malacological surveys and cercariometry. *Journal of Parasitic Diseases*, 46, 1090–1102. | |
| 254 | El-Shazly, A. M., Handoussa, A. E., & Hegazi, M. M., (1990). Fresh water malacologic fauna in Dakahlia governorate Egypt. *Journal of the Egyptian Society of Parasitology*, 20(2), 647-652. | |
| 255 | El-Zeiny, M. E., Ghoneim, A. M., Samak, O. A. A., & Khidr, A. A., (2021). Abundance and annual distribution of freshwater snails and some trematode cercariae at Damietta Governorate, Egypt. *Helminthologia*, 58(3), 233-247. | |
| 256 | Emery, A. M., Loxton, N. J., Stothard, R., Jones, C. S., Spinks, J., Llewellyn-Hughes, J., Noble, L. R., & Rollinson, D., (2003). Microsatellites in the freshwater snail Bulinus globosus (Gastropoda: Planorbidae) from Zanzibar. *Molecular Ecology Notes*, 3(1), 108-110. | |
| 257 | Epa, Y. R., Stigall, A. L., Roberts, E. M., O'brien, H. D., & Stevens, N. J., (2018). Morphological diversification of Ampullariid gastropods (Nsungwe formation, late oligocene, Rukwa rift basin, Tanzania) is coincident with onset of east African rifting. *Papers in Palaeontology*, 4(3), 327-348. | |
| 258 | Erko, B., Balcha, F., & Kifle, D., (2006). The ecology of *Biomphalaria sudanica* in Lake Ziway, Ethiopia. *African Journal of Ecology*, 44(3), 347-352. | |
| 259 | Erko, B., Tedla, S., & Petros, B., (1991). Transmission of intestinal *Schistosomiasis* in Bahir dar, Northwest Ethiopia. *Ethiopian Medical Journal*, 29(4), 199-211. | |
| 260 | Evers, B.N., Madsen, H., & Stauffer, J.R., (2011). Crush-resistance of soft-sediment gastropods of Lake Malawi: implications for prey selection by Molluscivorous fishes. *Journal of Freshwater Ecology*, 26(1), 85-90. | |
| 261 | Evers, B.N., Madsen, H., McKaye, K.M., & Stauffer, J.R., (2006). The schistosome intermediate host, Bulinus nyassanus, is a 'preferred' food for the cichlid fish, Trematocranus placodon, at Cape Maclear, Lake Malawi. *Annals of Tropical Medicine and Parasitology,* 100(1), 75-85. | |
| 262 | Fain, A., (1951)a. *Lymnea (Radix) natalensis* undussumae of Martens, natural transmitter of *Fasciola gigantica* Cobbold in the Belgian Congo; experimental reproduction of the evolutive cycle of fluke. *Annales de la Societe Belge de Medecine Tropicale* (1920), 31(5), 531-539. | |
| 263 | Fain, A., (1951)b. Notes ecologiques et parasitologiques sur *Lymnaea (Galba) truncatula* Muller au Congo Belge. *Annales de la Societe Belge de Medecine Tropicale,* 31, 149-152. | |
| 264 | Fain, A., (1952). Biomphalaria alexandrina tanganiyicensis (E.A. Smith 1881) transmitter of Schistosoma mansoni at Lac Albert. *Annales de la Societe belge de medecine tropicale*, (1920) 32(3):217-20. | |
| 265 | Fain, A., Thienpont, D., & Deramee, O., (1953). Observa tions sur Schistosoma rodhaini Brumpt au Ruanda-Urundi et description de la cercaire de ce schistosome. *Annales de la Societe Belge de Medecine Tropicale*, 33(5), 423-444. | |
| 266 | Farooq, M., (1973). Historical development. In Epidemiology and Control of *Schistosomiasis* (*Bilharziasis*), 1-14. Ansari, N. (Ed.). Basle, Karger. | |
| 267 | Fashuyi, S.A., & Williams, M.O., (1977). Role of Chaetogaster-limnaei in dynamics of trematode transmission in natural-populations of freshwater snails. *Zeitschrift fur Parasitenkunde-Parasitology Research,* 54(1), 55-60. | |
| 268 | Fischer-Piette, E., (1942). Les Mollusques d’Adanson. *Journal de Conchyliologie*. Paris, 85, 103-374. | |
| 269 | Flemings, C. T., & Lemma, M. B. A., (1973). *Schistosomiasis* in Harar, Alemaya and the Damota Valley, Ethiopia. *Ethiopian medical journal* 11(4), 271-8. | |
| 270 | Flores, V., Viozzi, G., & Brant, S. V., (2021). A new schistosome (Digenea: Schistosomatidae) from the nasal tissue of South America black-necked swans, Cygnus melancoryphus (Anatidae) and the endemic pulmonate snail Chilina gibbosa. *Zootaxa*, 4948(3), 404-418. | |
| 271 | Foster, H., Leitch, J. G., & Bedstone, I., (1951). An account of a small outbreak of intestinal schistosomiasis in Eritrea. *Journal of the Royal Army Veterinary Corps*, 97(6), 455-460. | |
| 272 | Fouad, Y., Mohamed, E.M., & Menriet, Z.R., (1993). Helisoma duryi: Its present range of distribution and implications with schistosomiasis snails in Egypt. *Journal of the Egyptian Society of Parasitology*, 23(1), 195-211. | |
| 273 | Frandsen, F.F., (1979). Further-studies on the compatibility between s intercalatum from Cameroun and Zaire and species of bulinus. *Zeitschrift fur Parasitenkunde-Parasitology Research*, 58(2), 161-167. | |
| 274 | Fuss, A., Mazigo, H. D., & Mueller, A., (2020). Malacological survey to identify transmission sites for intestinal schistosomiasis on Ijinga Island, Mwanza, north-western Tanzania. *Acta Tropica*, 203, 105289. | |
| 275 | Gamet, A., Brottes, H., & Essomba, E., (1964). Parasitic and microbial etiologies of dysenteriform syndromes observed in central Cameroon, Yaounde region. *Bulletin de la Societe de Pathologie Exotique et de ses Filiales,* 57(2), 233-240. | |
| 276 | Garba, A., Kinde-Gazard, D., & Massougbodji, A., (2000). Preliminary evaluation of morbidity due to S. haematobium and S. mansoni in the area of the future Adjarala Dam in Benin]. *Sante (Montrouge, France),* 10(5), 323-8. | |
| 277 | [Gardelle, J., Hiernaux, P., Kergoat, L., & Grippa, M. (2010). Less rain, more water in ponds: a remote sensing study of the dynamics of surface waters from 1950 to present in pastoral Sahel (Gourma region, Mali). *Hydrology and Earth System Sciences*, 14, 309–324. https://doi.org/10.5194/hess- 14-309-2010](https://doi.org/10.5194/hess-%2014-309-2010) | |
| 278 | Gaye, P.M., Doucouré, S., Sow, D., Sokhna, C., & Ranque, S., (2023). Identification of Bulinus forskalii as a potential intermediate host of Schistosoma hæmatobium in Senegal. *PLoS Neglected Tropical Diseases,* 16(5), 1-12. 12. | |
| 279 | Gbedjissi, G.L., & Vala, J.C., (2014). Life-cycle of the Afrotropical snail-killing fly Sepedon (Parasepedon) ruficeps Becker, 1923. *African Invertebrates*, 55 (1), 1-17. | |
| 280 | Genner, M. J., & Michel, E., (2003). Fine-scale habitat associations of soft-sediment gastropods at Cape Maclear, Lake Malawi. *Journal of Molluscan Studies*, 69, 325-328. | |
| 281 | Genner, M. J., Todd, J. A., Michel, E., Erpenbeck, D., Jimoh, A., Joyce, D. A., Piechocki, A., & Pointier, J. P., (2007). Amassing diversity in an ancient lake: evolution of a morphologically diverse parthenogenetic gastropod assemblage in Lake Malawi. *Molecular Ecology*, 16(3), 517-530. | |
| 282 | Genner, M. J., Michel, E., & Todd, J. A., (2008). Resistance of an invasive gastropod to an indigenous trematode parasite in Lake Malawi. *Biological Invasions*, 10(1), 41-49. | |
| 283 | Germain, L., (1908). Mollusques du Lac Tanganyika et de ses environs. Extrait des resultats secientifiques des voyages en Afrique d’Edouard Foa. *Bulletin du Muséum National d'histoire Naturelle*, 14, 1–612. | |
| 284 | Germain, L., (1920). Mollusques terrestres et fluviatiles. Première Partie. Voyage de M. Guy Babault dans l’Afrique Orientale Anglaise. *Résultats scientifiques*, 1-259. Paris, Blondel. | |
| 285 | Germain, L., (1923). Mollusques terrestres et fluviatiles. [Deuxième Partie]. Voyage de M. Guy Babault dans l’Afrique Orientale Anglaise. *Résultats scientifiques*, 1-149. Paris, Blondel. | |
| 286 | Germain, L., (1925). La composition et l’origine de la faune malacologique des Iles du Golfe de Guinée. *Comptes Rendus du Congrès de Sociétés Savantes de Paris et des Départments. Section des Sciences,* 487-503. | |
| 287 | Gillet, J., & Wolfs, J., (1954). Les bilharzioses humaines. au Congo Belge et au Ruanda-Urundi. *The Bulletin of the World Health Organization,* 10(3), 315–419. | |
| 288 | Gillet, J., (1950). Contribution to the study of urinary bilharziosis in the Belgian Congo. *Annales de la Societe Belge de Medecine Tropicale* (1920), 30(2), 195-204. | |
| 289 | Gillet, J., (1960). Note on a focus of murine Schistosoma rodhaini Brumpt bilharziasis in Kivu. A*nnales de la Societe Belge de Medecine Tropicale,* 40, 639-41. | |
| 290 | Gillet, J., Bruaux, P., & Lukall, G., (1961). Field trial with the new molluskicide "Bayer 73". *Annales de la Societe Belge de Medecine Tropicale,* 41(1), 35-43. | |
| 291 | Gillet, J., Bruaux, P., & Wolfs, J., (1960). Results of deep malacological prospecting in Lake Kivu and research on the survival of Biomphalaria in deep water. *Annales de la Societe belge de medecine tropicale*, (1920) 40, 643-9. | |
| 292 | Glaubrecht, M., (2008). Adaptive radiation of thalassoid gastropods in Lake Tanganyika, East Africa: morphology and systematization of a paludomid species flock in an ancient lake. *Zoosytematics and Evolution*, 84(1), 71-122. | |
| 293 | Glaubrecht, M. & Strong, E. E. 2007. Ancestry to an endemic radiation in Lake Tanganyika? Evolution of the viviparous gastropod Potadomoides Leloup, 1953 in the Congo River system (Cerithioidea, Paludomidae). *Biological Journal of the Linnean Society*, 92, 367-401. | |
| 294 | Glaubrecht, M. & Strong, E. E., (2004). Spermatophores of thalassoid gastropods (Paludomidae) in Lake Tanganyika, East Africa, with a survey of their occurrence in Cerithioidea: functional and phylogenetic implications. *Invertebrate Biology,* 123(3), 218-236. | |
| 295 | Goll, P. H., (1981). Mixed populations of Bulinus senegalensis (Muller) and Bulinus forskali (Ehrenburg) (Mollusca: Planorbidae) in The Gambia. *Transactions of the Royal Society of Tropical Medicine and Hygiene*, 75(4), 576-578. | |
| 296 | Goll, P. H., & Scott, J. M., (1978). The Interrelationship of Lymnaea Truncatula and Ovine Fascioliasis in the Ethiopian Central Highlands. *British Veterinary Journal*, 134 (6), 551-555. | |
| 297 | Goodman, J. D., & Panesar, T. S., (1976). Parasymphylodora manteri n. sp., an Adult Trematode from a Fresh-Water Pulmonate Snail, Biomphalaria choanomphala choanomphala, from Lake Victoria, Uganda.*Transactions of the American Microscopical Society,* 95(2), 204-209. | |
| 298 | Gouvras, A. N., Allan, F., Kinung’hi, S., Rabone, M., Emery, A., Angelo, T., Pennance, T., Webster, B., Nagai, H., & Rollinson, D., (2017). Longitudinal survey on the distribution of *Biomphalaria sudanica* and B. *choanomophala* in Mwanza region, on the shores of Lake Victoria, Tanzania: implications for schistosomiasis transmission and control. Parasites Vectors 10, 316. https://doi.org/10.1186/s13071-017-2252-z | |
| 299 | Gow, J. L., Noble, L. R., Rollinson, D., & Jones, C. S., (2001). Polymorphic microsatellites in the African freshwater snail, Bulinus forskalii (Gastropoda, Pulmonata). *Molecular Ecology Resources*, 1(4), 237-240. | |
| 300 | Gow, J. L., Noble, L. R., Rollinson, D., & Jones, C. S., (2005)a. A high incidence of clustered microsatellite mutations revealed by parent-offspring analysis in the African freshwater snail, *Bulinus forskalii* (Gastropoda, Pulmonata). *Genetica,*  124, 77–83. | |
| 301 | Gow, J. L., Noble, L. R., Rollinson, D., Tchuente, L. A. T., & Jones, C. S., (2005)b. High Levels Of Selfing Are Revealed By A Parent-Offspring Analysis Of The Medically Important Freshwater Snail, *Bulinus Forskalii* (Gastropoda: Pulmonata). *Journal of Molluscan Studies*, 71(2), 175-180. | |
| 302 | Gow, J. L., Noble, L. R., Rollinson, D., Tchuenté, L. A. T., & Jones, C. S., (2007). Contrasting temporal dynamics and spatial patterns of population genetic structure correlate with differences in demography and habitat between two closely-related African freshwater snails. *Biological Journal of the Linnean Society*, 90(4), 747-760. | |
| 303 | Graber, M. M., (1974). Mollusca of subgenus *Bulinus ss* (Basommatophora, Planorbidae), intermediate hosts of various parasitic trematodes in domestic ruminants of Ethiopia. *Comptes rendus hebdomadaires des seances de l academie des sciences serie D* 278(16), 2037-2039. | |
| 304 | Greer, G. J., Mimpfoundi, R., & Ratard, R. C., (1990). Human Schistosomiasis in Cameroon .2. distribution of the snail hosts. *American Journal of Tropical Medicine and Hygiene*, 42(6), 573-580. | |
| 305 | Gretillat, S., & Gaston, G., (1975). Remarks on the ecological characteristics of the snail trematode vectors in the dallol region niger. *Annales de Parasitologie Humaine et Comparee*, 50(5), 595-601. | |
| 306 | Griffiths, C. L., Robinson, T. B., Lange, L., & Mead, A., (2010). Marine biodiversity in South Africa: An evaluation of current states of knowledge. *PLoS ONE*, 5(8), e12008. https://doi.org/10.1371/journal.pone.0012008 | |
| 307 | Gryseels, B., & Nkulikyinka, l., (1988). The distribution of Schistosoma-mansoni in the Rusizi plain (Burundi). *Annals of Tropical Medicine and Parasitology*, 82(6), 581-590. | |
| 308 | Gryseels, B., (1985). The distribution of Biomphalaria and the transmission of Schistosoma in the Ruzizi-valley, Burundi - preliminary-study. *Annales de la Societe Belge de Medecine Tropicale,* 65(1), 49-58. | |
| 309 | Gryseels, B., (1991).The epidemiology of *Schistosomiasis* in Burundi and its consequences for control. *Transactions of the Royal Society of Tropical Medicine and Hygiene,* 85(5), 626-633. | |
| 310 | Gundersen, S.G., (1982). *Schistosoma-haematobium* in Welega, western Ethiopia - a report on 3 cases. *Ethiopian medical journal*, 20(2), 81-83. | |
| 311 | Gurarie, D., Lo, N.C., Ndeffo-Mbah, M.L., Durham, D.P., & King, C.H., (2018). The human-snail transmission environment shapes long term schistosomiasis control outcomes: Implications for improving the accuracy of predictive modeling. *PLoS Neglected Tropical Diseases,* 12(5), 1-19. | |
| 312 | Hailegebriel, T., Nibret, E., & Munshea, A., (2022). Distribution and seasonal abundance of Biomphalaria snails and their infection status with Schistosoma mansoni in and around Lake Tana, northwest Ethiopia. *Scientific Reports,* 12(1). | |
| 313 | Hamed, N., Hammami, H., Khaled, S., Rondelaud, D., & Ayadi, A., (2009). Natural infection of *Fasciola hepatica* (Trematoda: Fasciolidae) in *Bulinus truncatus* (Gastropoda: Planorbidae) in northern Tunisia. *Journal of Helminthology* 83(3), 271-273. | |
| 314 | Hammond, J. A., (1965). Observations on fascioliasis in Tanganyika. Bulletin of epizootic diseases of Africa. *Bulletin des epizooties en Afrique*, 13, 55-65. | |
| 315 | Hammou, L., Eline, B., & Madsen, H., (2005). Trematode infection among freshwater gastropods in Tessaout Amont irrigation system, Morocco. *African Zoology*, 40 (1), 77-82. | |
| 316 | Hammouda, S. A., (2023). Palaeoenvironments, Palaeoecology, and Palaeoclimate of the “Paleogene Hamadian deposits” west of Bechar (northwestern Sahara, Algeria). *Palaeobiodiversity and Palaeoenvironments,* 103, 413–431. | |
| 317 | Haroun, N. H., Roushdy, M. Z., & Mostafa, B. B., (1996). Effect of X-ray on the snails of schistosomiasis in Egypt. *Journal of the Egyptian Society of Parasitology*, 26(2), 383-392. | |
| 318 | Hassan, A. H. A. I., Ahmed, A. E. A. A. R. M., Lee, Y. H., & Elhag, M. S., (2021). Population Dynamics of Intermediate-Host Snails in the White Nile River, Sudan: A Year-Round Observational Descriptive Study. *The Korean journal of parasitology*, 59(2), 121-129. | |
| 319 | Hassnaa, A. S., Hoda, A. K., & Khaled, M. Z., (2022). Assessment of B*acillus aerius* and *Bacillus toyonensis* extracts as Pestcontrol agents against *Biomphalaria alexandrina* snails. *Aquaculture Research,* 53(3) | |
| 320 | Hauffe, T., Schultheiß, R., Van Bocxlaer, B., Prömmel, K., & Albrecht, C. (2016). Environmental heterogeneity predicts species richness of freshwater mollusks in sub-Saharan Africa. International Journal of Earth Sciences, 105(6), 1795–1810. doi: 10.1007/s00531-014-1109-3 | |
| 321 | Hayes, K. A., Cowie, R. H. & Thiengo, S. C. (2009)a. A global phylogeny of apple snails: Gondwanan origin, generic relationships, and the influence of outgroup choice (Caenogastropoda: Ampullariidae). *Biological Journal of the Linnean Society*, 98, 61-76. | |
| 322 | Hayes, K. A., Cowie, R. H., Jørgensen, A., Schultheiß, R. Albrecht, C. & Thiengo, S. C. (2009)b. Molluscan models in evolutionary biology: Apple snails (Gastropoda: Ampullariidae) as a system for addressing fundamental questions. *American Malacological Bulletin*, 27, 47-58. | |
| 323 | Hechinger, R. F., Lafferty, K. D., & Kuris, A. M., (2008). Trematodes indicate animal biodiversity in the Chilean intertidal and Lake Tanganyika. *Journal of Parasitology,* 94(4), 966-968. | |
| 324 | [Hernegger, M., Stecher, G., Schwatke, C., & Olang, L., (2021). Hydroclimatic analysis of rising water levels in the Great Rift Valley lakes of Kenya. *Journal of Hydrology Regional Studies,* (36), 100857. https://doi.org/10.1016/j. ejrh.2021.100857](https://doi.org/10.1016/j.%20ejrh.2021.100857) | |
| 325 | Hira, P. R., & Muller, R., (1966). Studies on Ecology of snails transmitting urinary Schistosomiasis in Western Nigeria. *Annals of Tropical Medicine and Parasitology,* 60(2), 198-211. | |
| 326 | Hofkin, B. V., Mkoji, G. M., & Loker, E. S., (1991). Control of Schistosome-transmitting snails in Kenya by the North-American crayfish Procambarus-clarkii. *American journal of tropical medicine and hygiene,* 45(3), 339-344. | |
| 327 | [Hotez, P. J., Molyneux, D. H., Fenwick, A., Kumaresan, J., Sachs, S. E., Sachs, J. D., & Savioli, L., (2007). Control of Neglected Tropical Diseases. New England Journal of Medicine, 357(10), 1018–1027. https://doi.org/10.1056/NEJMra064142](https://doi.org/10.1056/NEJMra064142) | |
| 328 | Hubendick, B., (1952). On the evolution of the so-called thalassoid molluscs of Lake Tanganyika. *Arkiv för Zoologi*, 3, 319–23. | |
| 329 | Hubendick, B., (1977). Freshwater gastropods of Sierra Leone*. Acta Regiae Societatis Scientarum et Litterarum Gothoburgensis, Zoologia,* 11, 1-30. | |
| 330 | Hudleston, W. H., (1904). On the origin of the marine (halolimnic) fauna of Lake Tanganyika. *Geological Magazine,* 1, 337–82. | |
| 331 | Ibikounlé, M., Gbédjissi, L. G., Ogouyèmi-Hounto, A., Batcho, W., Kindé-Gazard, D., & Massougbodji, A., (2014). Schistosomiasis and soil-transmitted helminthiasis among schoolchildren of Nikki and Pèrèrè, two northeastern towns of Benin. *Bulletin de la Société de Pathologie Exotique,* 107, 171–176. | |
| 332 | Ibikounlé, M., Mouahid, G., Mintsa Nguema, R., Sakiti, N., Massougbodji, A., & Moné, H., (2013). Snail intermediate host/Schistosoma haematobium relationships from three transmission sites in Benin (West Africa). *Parasitology Research*, 112, 227–233. | |
| 333 | Ibikounlé, M., Mouahid, G., Sakiti, N. G., Massougbodji, A., & Moné, H., (2009). Freshwater snail diversity in Benin (West Africa) with a focus on human schistosomiasis. *Acta Tropica*, 111(1), 29-34. | |
| 334 | Ibikounlé, M., Ogouyèmi-Hounto, A., Sissinto Savi de Tové, Y., Dansou, A., Courtin, D., Kindé-Gazard, D., Mouahid, G., Moné, H., & Massougbodji, A., (2014). Epidemiology of urinary schistosomiasis among school children in Péhunco area, Northern Benin. Malacological survey. *Bulletin de la Société de Pathologie Exotique*, 107, 177–184. | |
| 335 | Ibrahim, A. M., & Ghoname, S. I., (2018). Experimental Parasitology Molluscicidal impacts of Anagallis arvensis aqueous extract on biological, hormonal, histological and molecular aspects of *Biomphalaria alexandrina* snails. *Experimental Parasitology,* 192, 36-41. | |
| 336 | Ibrahim, M. M., (2007). Prevalence and intensity of Angiostrongylus cantonensis in freshwater snails in relation to some ecological and biological factors. *Parasite*, 14, 61–70 | |
| 337 | Itagaki, H., Suzuki, N., Ito, Y., Hara, T., & Wonde, T., (1975). Study on the Ethiopian freshwater molluscs, especially on identification, distribution and ecology of vector snails of human S*chistosomiasis.* *Japanese Journal of Tropical Medicine and Hygiene*, 3, 107-134. | |
| 338 | Janz, G. J., & Morais de Carvalho, A., (1956). Supplements on the study of bilharzioses in Angola. *Anais do Instituto de Higiene e Medicina Tropical,* 13(4), 597-613. | |
| 339 | Jelnes, J. E., (1983). Bulinus browni Jelnes, 1979 (Gastropoda: Planorbidae), a member of the forskalii group, as intermediate host for Schistosoma bovis in western Kenya. *Transactions of the Royal Society of Tropical Medicine and Hygiene,* 77(4), 566-566. | |
| 340 | Jickeli, C. F., (1874). Fauna de Land-und Süsswasser Mollusken Nord-Ost-Afrikas. *Nova Acta Academiae Caesareae Leopoldino-Carolinae*, 37, 1-352. | |
| 341 | Jobin, W. R., Negronaponte, H., & Michelson, E. H., (1976). Schistosomiasis in gorgol valley of Mauritania. *American journal of tropical medicine and hygiene,* 25(4), 587-594. | |
| 342 | Johnson, C. C., Njau, J. K., Van Damme, D., Schick, K., & Toth, N., (2016). Paleoecologic significance of malacofauna, Olduvai gorge, Tanzania. *Palaios,* 31(6), 319-326. | |
| 343 | Jones, C. S., Noble, L. R., Ouma, J., Kariuki, H. C., Mimpfoundi, R., Brown, D. S., & Rollinson, D., (1999). Molecular identification of schistosome intermediate hosts: case studies of Bulinus forskalii group species (Gastropoda: Planorbidae) from Central and East Africa. *Biological Journal of the Linnean Society,* 68(1–2), 215-240. | |
| 344 | Jones, R., Hill, J., & Hill, M., (2017). The abundance of an invasive freshwater snail *Tarebia granifera* (Lamarck, 1822) in the Nseleni River, South Africa. *African Journal of Aquatic Science*, 42(1):75-81. | |
| 345 | Joof, E., Sanneh, B., Sambou, S. M., & Wade, C.M., (2021). Species diversity and distribution of schistosome intermediate snail hosts in The Gambia. PLoS Neglected Tropical Diseases 15(10), 1-18. | |
| 346 | Jordan, P., (1996). Schistosomiasis in Tanzania long term results of TWSb and lucanthone hydrochloride combined in suppressive therapy in *Schistosoma haematobium* infection. *Transactions of The Royal Society of Tropical Medicine and Hygiene*, 60(1), 83–88. https://doi-org.ukzn.idm.oclc.org/10.1016/0035-9203(66)90188-X | |
| 347 | Jorgensen, A., Jorgensen, L. V. G., Kristensen, T. K., Madsen, H., & Stothard, J. R., (2007). Molecular phylogenetic investigations of Bulinus (Gastropoda: Planorbidae) in Lake Malawi with comments on the topological incongruence between DNA loci. Z*oologica Scripta,* 36(6), 577-585. | |
| 348 | Jorgensen, A., Kristensen, T. K., & Stothard, J. R., (2007). Phylogeny and biogeography of African Biomphalaria (Gastropoda: Planorbidae), with emphasis on endemic species of the great East African lakes. *Zoological Journal of the Linnean Society*, 151(2), 337-349. | |
| 349 | Joubert, P. H., & Pretorius, S. J., (1991). Laboratory evaluation of b-2 as a molluscicide in the control of the snail intermediate hosts of Schistosomiasis in South-Africa. *Annals of Tropical Medicine and Parasitology,* 85(4), 447-453. | |
| 350 | Julvez, A., Ali Halidi, M. A., & Brown, D. S., (1990). Inventaire des mollusques d’eau douce à Mayotte, archipel des Comores. *Revue d’Elevage et de Médecine vétérinaire des Pays tropicaux,* 43, 173-176. | |
| 351 | Kaatano, G. M., Min, D. Y., Siza, J. E., Yong, T. S., Chai, J. Y., Ko, Y., Chang, S. Y., Changalucha, J. M., Eom, K. S., & Rim, H. J., (2015). *Schistosoma mansoni*-Related Hepatosplenic Morbidity in Adult Population on Kome Island, Sengerema District, Tanzania. *Korean Journal of Parasitology*, 53(5), 545-551. | |
| 352 | Kabatereine, N. B., OdongoAginya, E. I., & Lakwo, T. L., (1996). *Schistosoma mansoni* along Lake Albert, Kibale district, western Uganda. *East African Medical Journal,* 73(8), 502-504. | |
| 353 | Kafumbata, D., Jamu, D., & Chiotha, S., (2014). Riparian ecosystem resilience and livelihood strategies under test: Lessons from Lake Chilwa in Malawi and other lakes in Africa. *Philosophical Transactions of the Royal Society B: Biological Sciences*, 369(1639), 1–8. | |
| 354 | Kagabo, J., Kalinda, C., Nshimiyimana, P., Mbonigaba, J.B., Ruberanziza, E., Nyandwi, E., & Rujeni, N., (2023). Malacological Survey and Spatial Distribution of Intermediate Host Snails in Schistosomiasis Endemic Districts of Rwanda. *Tropical Medicine and Infectious Disease*, 8(6)295. | |
| 355 | Kaiglová, A., Changoma, M.J.S., Špajdelová, J., Jakubcová, D., & Bírová, K., (2020). Urinary *Schistosomosis* in patients of rural medical health centers in Kwale county, Kenya. *Helminthologia,* 57(1), 19 - 27. | |
| 356 | Kandie, F. J., Krauss, M., Massei, R., Ganatra, A., Fillinger, U., Becker, J., Liess, M., Torto, B., & Brack, W., (2020). Multi-compartment chemical characterization and risk assessment of chemicals of emerging concern in freshwater systems of western Kenya. *Environmental Science Europe,* 32(1). | |
| 357 | Kapour, G. K. K., Wambui, C.W., Madinga, J., Huyse, T., Mitashi, P., (2024)a. Mapping of intermediate host snails for schistosomiasis in the Democratic Republic of Congo: a systematic review. *Folia Parasitologica*, 71: 010. https://doi:10.14411/fp.2024.010 | |
| 358 | Kapour, G. K. K., Wambui, C. W., Schols, R., Ntwan, J. M., Mulopo, P. M., & Huyse, T., (2024)b. First record of alien species of Amerianna strand, 1928 (Gastropoa: Hygrophila: Planorbidae) in the Kimpese region, Democratic Republic of Congo. *Folia Malacologica*, https://doi.org/10.12657/folmal.032.010 | |
| 359 | Karanja, D. M. S., Colley, D. G., Nahlen, B. L., Ouma, J. H., & Secor, W. E., (1997). Studies on *Schistosomiasis* in western Kenya .1. Evidence for immune-facilitated excretion of schistosome eggs from patients with *Schistosoma mansoni* and human immunodeficiency virus coinfections. *American Journal of Tropical Medicine and Hygiene,* 56(5), 515-521. | |
| 360 | Kariuki, H. C., Ivy, J. A., Muchiri, E. M., Sutherland L. J., & King, C. H., (2017). Divergent Effects of *Schistosoma haematobium* Exposure on Intermediate-Host Snail Species *Bulinus nasutus* and *Bulinus globosus* from Coastal Kenya. *The American journal of tropical medicine and hygiene,* 96 (4), 850-855. | |
| 361 | Kazibwe, F., Makanga, B., Rubaire-Akiiki, C., Ouma, J., Kariuki, C., Kabatereine, N. B., Booth, M., Vennervald, B. J., Sturrock, R. F., & Stothard, J. R., (2006). Ecology of *Biomphalaria* (Gastropoda: Planorbidae) in Lake Albert, Western Uganda: snail distributions, infection with schistosomes and temporal associations with environmental dynamics. *Hydrobiologia,* 568, 433-444. | |
| 362 | Kebede, T., Negash, Y., & Erko, B., (2018). *Schistosoma mansoni* infection in human and nonhuman primates in selected areas of Oromia Regional State, Ethiopia. *Journal of Vector Borne Diseases,* 55(2), 116-121. | |
| 363 | Kechemir, N., (1980). Description and life-cycle of Echinoparyphium-combesi sp-n in Bulinus-truncatus, vector of Schistosoma-haematobium in Algeria. *Annales de Parasitologie Humaine et Comparee,* 55(1), 57-68. | |
| 364 | Kechemir, N., (1987). Mollusques lies au probleme des schistosomoses en Algerie. *Archives de l'Institut Pasteur d'Algerie,* 55, 215-237. | |
| 365 | Kechemir, N., (1988). Trematodes of Algerian *Bulinus-truncatus* mollusca Planorbidae. *Archives de l'Institut Pasteur d'Algerie,* 56, 161-186. | |
| 366 | Kefi, A. S., Madsen, H., Likongwe, J. S., Jere, W. & Stauffer, J. R., (2012). Prey selection under laboratory conditions by pond-bred Trematocranus placodon (Regan, 1922), a molluscivorous cichlid from Lake Malawi. *Journal of Freshwater Ecology,* 27(4), 517-526. | |
| 367 | Kela, S. L., Istifanus, W. A., & Okwuosa, V. N., (1990). Note on the ecology and distribution of fresh water snails in the Bauchi and Plateau States, Nigeria. *Revue d'Elevage et de Medecine Veterinaire des Pays Tropicaux,* 43(2), 177-178. | |
| 368 | Kemp, M., De Kock, K. N., Zaayman, J. L., & Wolmarans, C. T., (2016). A comparison of mollusc diversity between the relatively pristine Marico River and the impacted Crocodile River, two major tributaries of the Limpopo River, South Africa. *Water SA,* 42(2), 253-260. | |
| 369 | Khallaayoune, K., Laamrani, H., & Madsen, H., (1998). Distribution of Bulinus truncatus, the intermediate host of Schistosoma haematobium, in an irrigation system in Morocco. *Journal of Freshwater Ecology,* 13(1), 129-133. | |
| 370 | Khalloufi, N., Bejaoui, M., & Delicado, D., (2017). A new genus and species of uncertain phylogenetic position within the family Hydrobiidae (Caenogastropoda, Truncatelloidea) discovered in Tunisian springs. *European Journal of Taxonomy*, 328, 1-15. | |
| 371 | [Kilburn, R. N., (1999). A brief history of marine malacology in South Africa. T*ransactions of the Royal Society of South Africa,* 54(1), 31–41. https://doi.org/10.1080/00359199909520402](https://doi.org/10.1080/00359199909520402) | |
| 372 | Kimura, E., Uga, S., Migwi, D. K., Mutua, W. R., Kiliku, F. M., & Muhoho, N. D., (1994). Hourly change in cercarial densities of Schistosoma haematobium and S. bovis at different depths in the water and distances from the shore of a dam in Kwale District, Kenya. *Tropical Medicine and Parasitology,* 45(2), 112-114. | |
| 373 | [King, C. H., Yoon, N., Wang, X., Lo, N. C., Alsallaq, R., Ndeffo-Mbah, M., Li, E., & Gurarie, D., (2020). Application of *Schistosomiasis* Consortium for Operational Research and Evaluation Study Findings to Refine Predictive Modeling of Schistosoma mansoni and Schistosoma haematobium Control in Sub-Saharan Africa. *The American Journal of Tropical Medicine and Hygiene,* 103(1 Suppl. l), 97–104. https://doi.org/10.4269/ajtmh.19-0852](https://doi.org/10.4269/ajtmh.19-0852) | |
| 374 | King, P. H., & Van As, J. G., (2000). Morphology and life history of *Petasiger variospinosus* (Trematoda: Echinostomatidae) in the Free State, *South Africa. Journal of Parasitology,* 86(2), 312-318. | |
| 375 | King, P. H., & van As, J. G., (2001). Cercariae shed by *Bulinus tropicus* (Krauss, 1848) in the Free State, South Africa. *African Zoology,* 36(1), 95-105. | |
| 376 | King, P. H., & vanAs, J. G., (1996). A description of the life stages of *Echinoparyphium elegans* (Trematoda: Echinostomatidae). *South African Journal of Zoology*, 31(3), 145-153. | |
| 377 | Kinoti, G., (1964). Observations on the transmission of *Schistosoma haematobium* and *Schistosoma bovis* in the Lake Region of Tanganyika. *Bulletin of the World Health Organisation,* 31(6), 815–823. | |
| 378 | Kloos, H., & Lemma, A., (1974). *Bilharziasis* in the Awash Valley: II. Molluscan fauna in irrigation farms and agricultural development. *Ethiopian medical journal,* 12(4), 157-73. | |
| 379 | Kloos, H., Lemma, A., & Desole, G., (1978). *Schistosoma-mansoni* distribution in ethiopia - study medical geography. *Annals of tropical medicine and parasitology,* 72(5), 461-470. | |
| 380 | Kloos, H., Lo, C.T., Birrie, H., Ayele, T., Tedla, S., & Tsegay, F., (1988). *Schistosomiasis* in Ethiopia. *Social Science & Medicine,* 26(8), 803-827. | |
| 381 | Klopper, R. R., Smith, G. F., & Chikuni, A. C., (2002). The Global Taxonomy Initiative in Africa. *Taxon,* 51, 159-165. | |
| 382 | Klumpp, R. K., & Chu, K. Y., (1980). Importance of the aquatic weed Ceratophyllum to transmission of *Schistosoma haematobium* in the Volta Lake, Ghana. *Bulletin of the World Health Organization,* 58(5), 791–798. | |
| 383 | Kobelt, W., (1909). Die Molluskenausbeute der Erlangerschen Reise in Nordost-Afrika. Ein Beitrag zur Molluskengeographie von Afrika. A*bhandlungen der Senckenbergischen Naturforschenden Gesellschaft,* 32, 1-97. | |
| 384 | Koblmüller, S., Duftner, N., Sefc, K. M., Aibara, M., Stipacek, M., Blanc, M., Egger, B., & Sturmbauer, C., (2007). Reticulate phylogeny of gastropod-shell-breeding cichlids from Lake Tanganyika – the result of repeated introgressive hybridization. *BMC Evolutionary Biology*, 7(7). https://doi.org/10.1186/1471-2148-7-7 | |
| 385 | Koeck, J. L., Modica, C., & Cavallo, J. D., (1999). Discovery of a focus of intestinal bilharziasis in te Republic of Djibouti. Medecine tropicale. *Revue du Corps de Sante Colonial,*59(1), 35-8. | |
| 386 | Köhler, F., & Glaubrecht, M., (2010). Uncovering an overlooked radiation: molecular phylogeny and biogeography of Madagascar's endemic river snails (Caenogastropoda: Pachychilidae: Madagasikara gen. nov.). *Biological Journal of the Linnean Society,* 99(4), 867-894. | |
| 387 | Köhler, F., Seddon, M., Bogan, A. E., Tu, D. T., Sri-Aroon, P., & Allen, D., (2012). The status and distribution of freshwater molluscs in the Indo-Burma region. In: Allen, D., Smith, K.G., Darwall, W.R.T. (Eds). *The Status and Distribution of Freshwater Biodiversity in the Indo-Burma region*. Chapter 4. Publisher: IUCN Gland Switzerland. | |
| 388 | Kokaliaris, C., Garba, A., Matuska, M., Bronzan, R. N., Colley, D. G., Dorkenoo, A. M., Ekpo, U. F., Fleming, F. M., French, M. D., Kabore, A., Mbonigaba, J. B., Midzi, N., Mwinzi, P. N. M., N'Goran, E. K. N., Polo, M. R., Sacko, M., Tchuem Tchuenté, L. A., Tukahebwa, E. M., Uvon, P. A., Yang, G., Wiesner, L., Zhang, Y., Utzinger, J., & Vounatsou, P., (2022). Effect of preventive chemotherapy with praziquantel on schistosomiasis among school-aged children in sub-Saharan Africa: a spatiotemporal modelling study. *Lancet Infectious Diseases,* 22(1), 136-149. | |
| 389 | Konan, C. K., Tian-Bi, Y. N. T., Diakite, N. R., Ouattara, M., Coulibaly, J. T., Diabate, S., Kone, A., Kalou, A. K., Assare, R. K., Ehouman, M. A., Glitho, S. C., & N'Goran, E. K., (2022). Spatial variation of life-history traits in Bulinus truncatus, the intermediate host of schistosomes, in the context of field application of niclosamide in Cote d'Ivoire. *BMC Zoology,* 7(1),7. | |
| 390 | Koudenoukpo, Z. C., Odountan, O. H., Van Bocxlaer, B., Sablon, R., Chikou, A., & Backeljau, T., (2020). Checklist of the fresh and brackish water snails (Mollusca, Gastropoda) of Bénin and adjacent West African ecoregions. *ZooKeys*, 942, 21-64. | |
| 391 | Krauss, F., (1848). *Die Südafrikanischen Mollusken*. Stuttgart: Ebner & Seubert. | |
| 392 | Krings, W., Neiber, M. T., Kovalev, A., Gorb, S. N., & Glaubrecht, M., (2021). Trophic specialisation reflected by radular tooth material properties in an "ancient" Lake Tanganyikan gastropod species flock. *BMC Ecology and Evolution*, 21(1), 35. | |
| 393 | Kristensen, T. K., (1985). *Guide Pratique des Gasteropodes d’Eau Douce Africains. 7. Especes presents en Afrique du North-Ouest.* Charlottenlund: : Danish Bilharziasis Laboratory. | |
| 394 | Kristensen, T. K., & Ogunnowo, O., (1987). Indoplanorbis-exustus (Deshayes, 1834), a fresh-water snail new for Africa, found in Nigeria (Pulmonata, Planorbidae). *Journal of Molluscan Studies,* 53, 245-246. | |
| 395 | Kristensen, T. K., & Brown, D. S., (1999). Control of intermediate host snails for parasitic diseases - A threat to biodiversity in African freshwaters? *Malacologia,* 41(2), 379-391. | |
| 396 | Laamrani, H., Khallaayoune, K., Boelee, E., Laghroubi,M.M., Madsen, H., & Gryseels, B., (2000). Evaluation of environmental methods to control snails in an irrigation system in Central Morocco. *Tropical Medicine & International Health,* 5(8), 545-52. | |
| 397 | Laamrani, H., Khallayoune, K., & Pointier, J.P., (1997). Factors affecting the distribution and abundance of two prosobranch snails in a thermal spring. *Journal of Freshwater Ecology,* 12(1), 75-79. | |
| 398 | Laamrani, H., Madsen, H., & Boelee, E., (2009). Micro-distribution of freshwater snails before and after water flow events in hydraulic structures in Tessaout Amont irrigation system, Morocco. *African Journal of Aquatic Science,* 34(1), 27-33. | |
| 399 | Lachish, T., Tandlich, M., Grossman, T., & Schwartz, E., (2013). High Rate of Schistosomiasis in Travelers After a Brief Exposure to the High-Altitude Nyinambuga Crater Lake, Uganda. *Clinical Infectious Diseases,* 57(10), 1461-1464. | |
| 400 | LaGrange, E., & Fain, A., (1952). New predator of fresh water pulmobranchiate molluscs as transmitters of bilharziasis. *Annales de la Societe Belge de Medecine Tropicale,* (1920) 32(1), 53-5. | |
| 401 | Laidemitt, M. R., Brant, S. V., Mutuku, M. W., Gerald M. Mkoji, G. M., & Loker, E. S., (2019). The diverse echinostomes from East Africa: With a focus on species that use *Biomphalaria* and *Bulinus* as intermediate hosts. *Acta Tropica,* 193, 38-49. | |
| 402 | Lalouel, J., (1954). Intestinal *Schistosomiasis* due to *S. intercalatum* at Libreville. *Bulletin de la Société de Pathologie Exotique,* 47(4), 531-534. | |
| 403 | Lange, C. N., Kristensen, T. K., & Madsen, H., (2013). Gastropod diversity, distribution and abundance in habitats with and without anthropogenic disturbances in Lake Victoria, Kenya. *African Journal of Aquatic Science,* 38(3), 295-304. | |
| 404 | Lapierre, J., Tourteschaefer, C., & Faurant, C., (1992). Complements on the epidemiologic-study of the focus of Schistosomiasis-mansoni of Kara (Northern Togo). *Bulletin de la Societe de Pathologie Exotique,* 85(3), 232-237. | |
| 405 | [Laraque, A., N’kaya, G. D. M., Orange, D., Tshimanga, R., Tshitenge, J. M., Mahé, G., Nguimalet, C. R., Trigg, M. A., Yepez, S., & Gulemvuga, G., (2020). Recent budget of hydroclimatology and hydrosedimentology of the Congo River in central Africa. Water, 12, 2613. https://doi.org/10.3390/w1209 2613](https://doi.org/10.3390/w1209%202613) | |
| 406 | Lariviere, M., Buttner, A., & Picot, H., (1969). Epidemiologic data on the Schistosoma heamatobium complex in South Saharan Africa. *Bulletin de la Societe de pathologie exotique et de ses filiales,* 62(2), 376-84. | |
| 407 | Lawton, S. P., Allan, F., Hayes, P. M., & Smit, N. J., (2018). DNA barcoding of the medically important freshwater snail *Physa acuta* reveals multiple invasion events into Africa. *Acta Tropica*, 188, 86-92. | |
| 408 | Leiper, R. T., (1915). Report on the results of the Bilharzia Mission in Egypt, 1915. 1. Transmission. 5. Adults and Ova. *Journal of the Royal Army Medical Corps,* 25, 1-55. | |
| 409 | Leiper, R. T., (1918). Report on the results of the Bilharzia Mission in Egypt, 1915. 1. Transmission. 5. Adults and Ova. *Journal of the Royal Army Medical Corps,* 30, 235-260. | |
| 410 | Leloup, E., (1953). Gastéropodes. *Exploration Hydrobiologique du Lac Tanganyika*, 3, 1-273. | |
| 411 | Leng, M. J., Lamb, A. L., Lamb, H. F., & Telford, R. J., (1999). Palaeoclimatic implications of isotopic data from modern and early Holocene shells of the freshwater snail Melanoides tuberculata, from lakes in the Ethiopian Rift Valley. *Journal of Paleolimnology,* 21(1), 97-106. | |
| 412 | Lévêque, C., (1967). Mollusques aquatiques de la zone est du Lac Tchad. *Bulletin de l’Institut Fondamental d’Afrique Noire, série A,* 4, 1494-1533. | |
| 413 | Levitz, S., Standley, C. J., Adriko, M., Kabatereine, N. B., & Stothard, J. R., (2013). Environmental epidemiology of intestinal schistosomiasis and genetic diversity of Schistosoma mansoni infections in snails at Bugoigo village, Lake Albert. *Acta Tropica*, 128(2), 284-291. | |
| 414 | Lietar, J., (1956). Biologie et ecologie des mollusques vecteurs de bilharziose a Jadotville. *Annales de la Societe Belge de Medecine Tropicale,* 36(6), 919-1036. | |
| 415 | Lindsey, P. A., Anderson, S. H., Dickman, A., Gandiwa, P., Harper, S., Morakinyo, A. B., Nyambe, N., O’Brien-Onyeka, M., Packer, C., Parker, A. H., Robson, A. S., Ruhweza, A., Sogbohossou, E. A., Steiner, K. W., & Tumenta, P. N., (2022). Shepherding Sub-Saharan Africa’s Wildlife through Peak Anthropogenic Pressure toward a Green Anthropocene. *Annual Review of Environment and Resources*, 47, 91-121. | |
| 416 | Logan, J. W. M., (1983). Schistosomiasis in swaziland - a comparative-study of 3 irrigated estates. *Journal of helminthology,* 57(3), 247-253. | |
| 417 | Loker, E. S., Moyo, H. G., & Gardner, S. L., (1981). Trematode–gastropod associations in nine non-lacustrine habitats in the Mwanza region of Tanzania. *Parasitology,* 83, 381-399. | |
| 418 | Loreau, M., & Baluku, B., (1987). Population-dynamics of the fresh-water snail *Biomphalaria-pfeifferi* in eastern Zaire. *Journal of Molluscan Studies,* 53, 249-265. | |
| 419 | Lukens, N. R., Kraemer, B. M., Constant, V., Hamann, E. J., Michel, E., Socci, A. M., Vadeboncoeur, Y., & McIntyre, P. B., (2017). Animals and their epibiota as net autotrophs: size scaling of epibiotic metabolism on snail shells. *Freshwater Science,* 36(2), 307-315. | |
| 420 | Lundeba, M., Likongwe, J. S., Maden, H., & Stauffer, J. R., (2006). Preliminary study on the culture and breeding of *Bulinus nyassanus* (Mollusca: Pulmonata) under laboratory conditions. *African Zoology,* 41(1), 143-144. | |
| 421 | Lundeba, M., Likongwe, J. S., Madsen, H., & Stauffer, J. J. R., (2007). Potential of *Metriaclima lanisticola* (Teleostei: Cichlidae) for biological control of schistosome intermediate host snails. *African Zoology,* 42(1), 45-49. | |
| 422 | Lundeba, M., Likongwe, J. S., Madsen, H., & Stauffer, J. R., (2011). Oral shelling of *Bulinus* spp. (Mollusca: Planorbidae) by the Lake Malawi cichlid, Metriaclima lanisticola (Pisces: Cichlidae). *Journal of Freshwater Ecology*, 26(4), 593-597. | |
| 423 | Mabrouk, Y., Glöer, P., & Taybi, A. F., (2023). The first record of the North American freshwater limpet Ferrissia californica (Mollusca, Gastropoda) in Morocco. *Nature Conservation Research,* 8(1). | |
| 424 | Mabrouki, Y., Gloer, P., & Taybi, A. F., (2022). Gyraulus marocana sp. nov., a new freshwater snail species (Mollusca, Gastropoda, Planorbidae) from Morocco. *Nature Conservation Research,* 7(1), 1-5. | |
| 425 | Machena, C., & Kautsky, N., (1988). A quantitative diving survey of benthic vegetation and fauna in Lake Kariba, a tropical man-made lake. *Freshwater Biology*, 19, 1-4. | |
| 426 | Madsen, H., (1983). Distribution of *Helisoma-duryi*, an introduced competitor of intermediate hosts of *Schistosomiasis*, in an irrigation scheme in Northern Tanzania. *Acta Tropica,* 40(3), 297-306. | |
| 427 | Madsen, H., (1992). Ecological-studies on the intermediate host snails and the relevance to *Schistosomiasis* control. *Memorias do Instituto Oswaldo Cruz,* 87, 249-253. | |
| 428 | Madsen, H., & Stauffer, J. R., (2011). Density of Trematocranus placodon (Pisces: Cichlidae): A Predictor of Density of the Schistosome Intermediate Host, *Bulinus nyassanus* (Gastropoda: Planorbidae), in Lake Malawi. *Ecohealth,* 8(2), 177-189. | |
| 429 | Madsen, H., Coulibaly, G., & Furu, P., (1987). Distribution of fresh-water snails in the river Niger basin in Mali with special reference to the intermediate hosts of schistosomes. *Hydrobiologia,* 146(1), 77-88. | |
| 430 | Madsen, H., Daffalla, A. A., Karoum, K. O., & Frandsen, F., (1988). Distribution of freshwater snails in irrigation schemes in the Sudan. *Journal of Applied Ecology*, 25, 853-866. | |
| 431 | Madsen, H., Kamanga, K. C. J., Stauffer, J. R., & Likongwe, J., (2010). Biology of the Molluscivorous Fish Trematocranus placodon (Pisces: Cichlidae) from Lake Malawi. *Journal of Freshwater Ecology,* 25(3), 449-455. | |
| 432 | Maes, T., De Corte, Z., Vangestel, C., Virgilio, M., Smitz, N., Djuikwo-Teukeng, F.F., Papadaki, M.L., & Huyse, T., (2022). Large-scale and small-scale population genetic structure of the medically important gastropod species Bulinus truncatus (Gastropoda, Heterobranchia). Parasites & Vectors 15(1), p1-13. | |
| 433 | Mahmoud, K. M. A., & Abu Taleb, H. M. A., (2013). Fresh water snails as bioindicator for some heavy metals in the aquatic environment. *African Journal of Ecology,* 51(2), 193-198. | |
| 434 | Mahulu, A., Stelbrink, B., Van Bocxlaer, B., Riedel, Frank, F., & Albrecht, C., (2021). Going with the flow? Diversification of gastropods reflects drainage evolution in Africa. *Journal of Biogeography,* 48(7), 1579-1593. | |
| 435 | Makherana, F., Cuthbert, R. N., Dondofema, F., Wasserman, R. F., Chauke, G. M., Munyai, L. F., & Dalu, T., (2022). Distribution, drivers and population structure of the invasive alien snail *Tarebia granifera* in the Luvuvhu system, South Africa. *River Research and Applications,* 38(8), 1362-1373. | |
| 436 | Makura, O., & Kristensen, T. K., (1991). National freshwater snail survey of Zimbabwe. In *Proceedings of the Tenth International Malacological Congress*, 227-232. Meier-Brook, C. (Ed.). Tübingen: Institute for Tropical Medicine and Unitas Malacologica. | |
| 437 | Malatji, M.P., Lamb, J., & Mukaratirwa, S., (2019). Molecular characterization of liver fluke intermediate host lymnaeids (Gastropoda: Pulmonata) snails from selected regions of Okavango Delta of Botswana, KwaZulu-Natal and Mpumalanga provinces of South Africa. *Veterinary Parasitology - Regional Studies and Reports,* 17, 2405-9390. | |
| 438 | Malatji, M.P., Myende, N., & Mukaratirwa, S., (2021). Are Freshwater Snails, Melanoides sp. and Invasive *Tarebia granifera* (Gastropoda: Thiaridae) Suitable Intermediate Hosts for *Calicophoron microbothrium* (Trematoda: Paramphistomoidea)? An Experimental Study. *Frontiers in Veterinary Science,* 8, 705954. | |
| 439 | Malek, E. A., & Chaine, J. P., (1981). Fresh-water snails of the Senegal river basin, west-Africa. *Nautilus,* 95(4), 193-198. | |
| 440 | Maluil, S., & Stevens, R. A., (2016). Clinical Report: *Schistosomiasis* Exposure in US Service Personnel During Whitewater Rafting on the Nile River in Jinja, Uganda. *Military Medicine,* 181(11), 1495-1498. | |
| 441 | Mandahl-Barth, G., (1954). The freshwater mollusks of Uganda and adjacent territories. *Annales du Musée Royal du Congo Belge, Tervuren, Série 8˚, Sciences Zoologiques*, 32:1-206. | |
| 442 | Mandahl-Barth, G., (1968). Freshwater molluscs. *Exploration Hydrobiologique du Bassin du Lac Bangweolo et du Luapula,* 12, 1-97. | |
| 443 | Mandahl-Barth, G., (1972). The freshwater Mollusca of Lake Malawi. *Revue de Zoologie et de Botanique Africaine,* 86, 257-289. | |
| 444 | Mandahl-Barth, G., (1988). Studies on African freshwater bivalves. Charlottenlund: Danish Bilharziasis Laboratory. Pp. 1-161. | |
| 445 | Mandahl-Barth, G., Malaisse, F., & Ripert, C., (1972). Etudes malacologiques dans la region du lac de retenue de la Lufira (Katanga). Distribution et ecologie des mollusques aquatiques. Role epidemiologique des vecteurs des bilharzioses intestinale et urinaire. *Bulletin de la Societe de Pathologie Exotique et de ses affiliales,* 65(1), 146-65. | |
| 446 | Mandahl-Barth, G., Ripert, C., & Raccurt, C., 1974. Nature du sous-sol, repartition des mollusques dulcaquicoles et foyers de bilharzioses intestinale et urinaire au Bas-Zaire. *Revue de Zoologie Africaine,* 88, 553-584. | |
| 447 | Manning, S. D., Woolhouse, M. E. J., & Ndamba, J., (1995). Geographic compatibility of the freshwater snail *Bulinus globosus* and schistosomes from the Zimbabwe highveld. *International Journal for Parasitology,* 25(1), 37-42. | |
| 448 | Manyangadze, T., Chimbari, M. J., Gebreslasie, M., Ceccato, P., & Mukaratirwa, S., (2016). Modelling the spatial and seasonal distribution of suitable habitats of schistosomiasis intermediate host snails using Maxent in Ndumo area, KwaZulu-Natal Province, South Africa. *Parasites & Vectors,* 9, 1-10. | |
| 449 | Manz, K. M., Kroidl, I., Clowes, P., Gerhardt, M., Nyembe, W., Maganga, L., Assisya, W., Ntinginya, N.E., Berger, U., Hoelscher, M., & Saathoff, E., (2020). Schistosoma haematobiuminfection and environmental factors in Southwestern Tanzania: A cross-sectional, population-based study. *Plos Neglected Tropical Diseases,* 14(8), e0008508. | |
| 450 | Marill, F. G., (1956). Irrigation system management methods and prophylaxis of vesical *Schistosomiasis*. *Bulletin de la Societe de Pathologie Exotique,* 49(2), 373-378. | |
| 451 | Marill, F. G., (1957). The growth of the Algerian focus of urinary *Schistosomiasis* at Inkermann-Saint-Aime. *Bulletin de la Societe de Pathologie Exotique,* 50(5), 794-804. | |
| 452 | Marill, F. G., (1958). Sur l'appréciation comparative de la richesse des gîtes en mollusques fluviatiles, notamment en Bulinus truncatus Audouin. *The Bulletin of the World Health Organization,* 18(5-6), 1057–1064. | |
| 453 | Marill, F. G., (1958). Sur les variations de la composition chimique de l'eau et les variations d'abondance de *Bulinus truncatus* Audouin. *The Bulletin of the World Health Organization,* 18(5-6): 1064–1070. Correction in: *The Bulletin of the World Health Organization,* 19(2), 400. | |
| 454 | Marill, F.G., (1953). Risks of diffusion of urinary bilharziasis in Algeria. *Minerva urologica,* 5 (4), 143-5. | |
| 455 | Marill, F.G., (1961). Enseigneruents d'une premiere enquete sur l'epidemiologie de la bilharziose a *Schistosoma haematobium* en Mauritanie. *Medecine Tropicale Marseilles,* 21, 373-386. | |
| 456 | Martens, E. von, (1860). Verzeichniss der von Prof. Peters in Mossambique gesammelten Landund *Süsswasser-Mollusken. Malakozoologische Blätter*, 6, 211-221. | |
| 457 | Martens, E. von, (1879). Ubersicht der von 1843 bis 1847 in Mossambique gesammelten Mollusca. *Monatsberichte der Königlichen Preussischen Akademie der Wissenschaft zu Berlin,* 44,727-749. | |
| 458 | Martens, E. von, (1897). *BeschalteWeichthiere*. Deutsch-Ost-Afrika 4: 1-308. Berlin, Dietrich Reimer (Ernst Vohsen). | |
| 459 | Marti, H., (1986). Field observations on the population-dynamics of Bulinus-globosus, the intermediate host of *Schistosoma-haematobium* in the Ifakara area, Tanzania. *Journal of Parasitology,* 72(1), 119-124. | |
| 460 | [Masese, F. O., Arimoro, F. O., Dalu, T., & Gettel, G. M., (2023). Editorial: Freshwater science in Africa. *Frontiers in Environmental Science*. https://doi.org/10.3389/fenvs.2023.1233932](https://doi.org/10.3389/fenvs.2023.1233932) | |
| 461 | Massot, M., & Senoucihorr, K., (1983). Distribution of Lymnaea-truncatula in the Northwest of Algeria and study of its receptivity to Fasciola-hepatica. *Annales de Parasitologie Humaine et Comparee,* 58 (1), 19-25. | |
| 462 | Mathole, M.C., & King, P.H., (2023). Molluscicidal and cercaricidal effects of Persicaria senegalensis on Radix natalensis snails and their echinostome-shed cercariae in South Africa. *African Journal of Aquatic Science*, 48(1), 71-76. | |
| 463 | Matos, L. M. M. de., Pile, E. A., & Ahid, S. M. M., (2011). Geopolitic distribuition of Hepatic fasciolosis on Santiago Island, Cape Verde. *Arquivos do Instituto Biológico,* 78(3), 435-437. | |
| 464 | Mbereko, A., Chimbari, M. J., Furu, P., Mukaratirwa, S., (2023). Health institutional dynamics in the management of malaria and bilharzia in Zimbabwe in the advent of climate change: A case study of Gwanda district. *Cogent Social Sciences*, 9, 2215632. https://doi.org/10.1080/23311886.2023.2215632 | |
| 465 | McClain, M. E., (2013). Balancing water resources development and environmental sustainability in Africa: A review of recent research findings and applications. *Ambio*, 42(5), 549–565. | |
| 466 | McClelland, W. F. J., & Jordan, P., (1962). Schistosomiasis at Bukoba, Tanganyika, on Lake Victoria. *Annals of Tropical Medicine & Parasitology,* 56(4), 396-400. | |
| 467 | McCreesh, N., Nikulin, G., & Booth, M., (2015). Predicting the effects of climate change on *Schistosoma mansoni* transmission in eastern Africa. *Parasites & Vectors,* 8(1), 283-301. | |
| 468 | McCullough, F. S., (1956). Transmission of Schistosoma haematobium by Bulinus sp. in the KE district of the Gold Coast. *Transactions of the Royal Society of Tropical Medicine and Hygiene,* 50(5), 449-457. | |
| 469 | McCullough, F. S., (1957). The seasonal density of populations of *Bulinus* (Physopsis) globosus and *B. forskalii* in natural habitats in Ghana. *Annals of Tropical Medicine and Parasitology* 51, 235-248. | |
| 470 | Mccullough, F. S., (1962). Observations on B*ulinus (Bulinus) truncatus rohlfsi* (clessin) in Ghana .1. distribution of snails and their role in transmission of Schistosoma haematobium. *Annals of tropical medicine and parasitology* 56(1), 53-60. | |
| 471 | McCullough, F. S., (1964). Observations on bilharziasis and the potential snail hosts in the Republic of the Congo (Brazzaville). *The Bulletin of the World Health Organization,* 30(3), 375–388. | |
| 472 | Mccullough, F. S., (1965). A note on intestinal Schistosomiasis and snail hosts in Ghana. *Annals of tropical medicine and parasitology,* 59(3), 312. | |
| 473 | McIntyre, P. B., Michel, E., France, K., Rivers, A., Hakizimana, P., & Cohen, A. S., (2005). Individual- and assemblage-level effects of anthropogenic sedimentation on snails in Lake Tanganyika. *Conservation Biology,* 19(1), 171-181. | |
| 474 | Mckaye, K. R., Stauffer, J. R., & Louda, S. M., (1986). Fish predation as a factor in the distribution of Lake Malawi Gastropods. *Experimental Biology,* 45(4), 279-289. | |
| 475 | Mcmahon, J. P., Highton, R. B., & Marshall, T. F. D. C., (1977). Studies on biological-control of intermediate hosts of *Schistosomiasis* in western Kenya. *Environmental conservation,* 4(4), 285-289. | |
| 476 | Meier-Brook, C., Haas, D., Winter, G., & Zeller, T., (1987). Hydrochemical factors limiting the distribution of *Bulinus truncates* (Pulmonata: Planorbidae). *American Malacological Bulletin*, 5, 85-90. | |
| 477 | Meleko, A., Li, S., Turgeman, D.B., Bruck, M., Kesete, N.Z., Zaadnoordijk, W., Rollinson, D., Sabar, G., Bentwich, Z., & Golan, R., (2022). Schistosomiasis Control in Ethiopia: The Role of Snail Mapping in Endemic Communities. Tropical Medicine and Infectious Disease 7(10), 272. | |
| 478 | Merad, N., Andreu, V., Chaib, S., de Cavalho Augusto, R., Duval, D., Bertrand, C., Boumghar, Y., Pichette, A., & Djabou, N., (2021). Essential Oils from Two Apiaceae Species as Potential Agents in Organic Crops Protection. Antibiotics 10(6), 636. | |
| 479 | Michel, A. E., (2000). Phylogeny of a gastropod species flock: exploring speciation in Lake Tanganyika in a molecular framework. *Advances in Ecological Research*, 31, 275-302. | |
| 480 | Michel, A. E., (2004). Vinundu, a new genus of Gastropod (Cerithioidea, ‘Thiaridae’) with two species from Lake Tanganyika, East Africa, and its molecular phylogenetic relationships. *Journal of Molluscan Studies,* 70(1):1-19. | |
| 481 | Michel, A. E., Cohen, A. S., West, K., Johnston, M. R. & Kat, P. W., (1992). Large African lakes as natural laboratories for evolution: examples from the endemic gastropod fauna of Lake Tanganyika. *Mitteilungen Internationale Vereinigung für Theoretische und Angewandte Limnologie*, 23, 85-99. | |
| 482 | Michel, A. E., McIntyre, P.B., & Chan, J., (2007). A snail's space sets a snail's pace: Movement rates of Lavigeria gastropods in lake Tanganyika, East Africa. *Journal of Molluscan Studies,* 73, 195-198. | |
| 483 | Michel, A. E., West, K., Todd, J. A., Brown, D. S. & Clabaugh, J., (2003). The Gastropods of Lake Tanganyika: Diagnostic Key and Taxonomic Classification with Notes on the Fauna. 132 pgs, 102 figures. SIL special publications (Societas Internationalis Limnologiae - International Assoc. of Theoretical and Applied Limnology). | |
| 484 | Miller, J.P., Delicado, D., García-Guerrero, F., Khalloufi, N., & Ramos, M.A., (2023). Morphology and taxonomic assessment of eight genetic clades of Mercuria Boeters, 1971 (Caenogastropoda, Hydrobiidae), with the description of five new species. European Journal of Taxonomy, 866(1), 1–63. | |
| 485 | Mimpfoundi, R., & Greer, G. J., (1990)a. Allozyme comparisons and ploidy levels among species of the *Bulinus-truncatus-tropicus* complex (Gastropoda, Planorbidae) in Cameroon. *Journal of Molluscan Studies* 56, 63-68. | |
| 486 | Mimpfoundi, R., & Greer, G. J., (1990)b. Allozyme variation among populations of *Biomphalaria-camerunensis* (boettger, 1941) (Gastropoda, Planorbidae) in Cameroon. *Journal of Molluscan Studies,* 56, 373-381. | |
| 487 | Mimpfoundi, R., & Greer, G. J., (1990)c. Allozyme variation among populations of *Bulinus-forskalii* (Ehrenberg, 1831) (Gastropoda, Planorbidae) in Cameroon. *Journal of Molluscan Studies,* 56, 363-371. | |
| 488 | Mimpfoundi, R., Dupouy, J., & Nassi, H., (1986). Genetic differentiation between populations of Biomphalaria-pfeifferi (Krauss, 1848) (Moll Planorbidae) originating from middle southern Cameroon and Senegal. Comptes Rendus des Seances de la Societe de Biologie et de ses Filiales 180(3), 290-295. | |
| 489 | Miranda, N. A. F., & Perissinotto, R., (2014)a. Effects of an alien invasive gastropod on native benthic assemblages in coastal lakes of the iSimangaliso Wetland Park, South Africa. *African Invertebrates*, 55(2), 209-228. | |
| 490 | Miranda, N. A. F., & Perissinotto, R., (2014)b. Benthic assemblages of wetlands invaded by *Tarebia granifera* (Lamarck, 1822) (Caenogastropoda: Thiaridae) in the iSimangaliso Wetland Park, South Africa. *Molluscan Research,* 34(1), 40-48. | |
| 491 | Miranda, N. A. F., Measey, G. J., & Appleton, C. C., (2016). Shell crushing resistance of alien and native thiarid gastropods to predatory crabs in South Africa. *Aquatic Invasions,* 11(3), 303-311. | |
| 492 | Miranda, N. A. F., Perissinotto, R., & Appleton, C. C., (2010). Salinity and temperature tolerance of the invasive freshwater gastropod *Tarebia granifera*. *South African Journal of Science,* 106(3/4), 1-7. | |
| 493 | Miranda, N. A. F., Perissinotto, R., & Appleton, C. C., (2011)a. Feeding dynamics of the invasive gastropod *Tarebia granifera* in coastal and estuarine lakes of northern KwaZulu-Natal, South Africa. *Estuarine Coastal and Shelf Science* 91(3), 442-449. | |
| 494 | Miranda, N. A. F., Perissinotto, R., & Appleton, C. C., (2011)b. Population Structure of an Invasive Parthenogenetic Gastropod in Coastal Lakes and Estuaries of Northern KwaZulu-Natal, South Africa. *PlosOne*, 6(8), e24337. | |
| 495 | Mkize, L. S., Mukaratirwa, S., & Zishiri, O. T., (2016). Population genetic structure of the freshwater snail, Bulinus globosus, (Gastropoda: Planorbidae) from selected habitats of KwaZulu-Natal, South Africa. *Acta Tropica,* 161, 91-99. | |
| 496 | Mnkandla, S. M., Siwela, A. H., & Basopo, N., (2019). Effects of chronic exposures of selected heavy metals on the glutathione S-transferase activity of freshwater snails *Lymnaea natalensis* in Zimbabwe. *African Journal of Aquatic Science,*44(3), 233-236. | |
| 497 | Moema, E. B. E., King, P. H., & Baker, C., (2008). Cercariae developing in Lymnaea natalensis Krauss, 1848 collected in the vicinity of Pretoria, Gauteng Province, South Africa. *Onderstepoort Journal of Veterinary Research,* 75(3), 215-23. | |
| 498 | Moema, E. B. E., King, P. H., & Baker, C., (2012). Descriptions of Strigea cercariae from the Gauteng and North West Provinces, South Africa. *Onderstepoort Journal of Veterinary Research,* 79(1), 1-8. | |
| 499 | Moema, E. B., King, P. H., & Rakgole, J. N., (2019). Phylogenetic studies of larval digenean trematodes from freshwater snails and fish species in the proximity of Tshwane metropolitan, South Africa. *Onderstepoort Journal of Veterinary Research,* 86(1), 1-7. | |
| 500 | Molaba, G. G., Molefe-Nyembe, N. I., Taioe, O. M., Mofokeng, L. S., Thekisoe, O. M. M., & Mtshali, K., (2023). Molecular detection of Fasciola, Schistosoma and Paramphistomum species from freshwater snails occurring in Gauteng and Free State provinces, South Africa. *Veterinary Parasitology,* 320, 109978. | |
| 501 | Mone, H., Ibikounle, M., & Mouahid, G., (2010). Human *Schistosomiasis* in the Economic Community of West African States: Epidemiology and Control. *Advances in Parasitology*, 7133-91. | |
| 502 | Moore, J. E. S., (1897). The fresh-water fauna of Lake Tanganyika. *Nature*, 56, 198–200. | |
| 503 | Moore, J. E. S., (1898)a. The molluscs of the Great African Lakes. I. Distribution. *Quarterly Journal of Microscopical Science,* 41, 159–80. | |
| 504 | Moore, J. E. S., (1898)b. The molluscs of the Great African Lakes. II. The anatomy of the Typhobias, with a description of the new genus (Bathanalia). *Quarterly Journal of Microscopical Science,* 41, 181–204. | |
| 505 | Moore, J. E. S., (1898)c. On the zoological evidence for the connection of Lake Tanganyika with the sea. *Proceedings of the Royal Society*, 62, 451–8. | |
| 506 | Moore, J. E. S., (1899)a. The molluscs of the Great African Lakes. III. *Tanganyikia rufofilosa*, and the genus *Spekia*. *Quarterly Journal of Microscopical Science*, 42, 155–85. | |
| 507 | Moore, J. E. S., (1899)b. The molluscs of the Great African Lakes. IV. Nassopsis and Bythoceras. *Quarterly Journal of Microscopical Science,* 42, 187–201. | |
| 508 | Moore, J. E. S., (1903). *The Tanganyika problem*. London: Burst and Blackett. | |
| 509 | Morais, J. A., (1975). Schistosomiasis mansoni in Angola: notes on its recent spreading. *Anais do Instituto de Higiene e Medicina Tropical,* 3(1-4), 405-23. | |
| 510 | Morelet, A., (1868). *Mollusques terrestres et fluviatiles.* Voyage du Dr Friedrich Welwitsch. Paris, Balliére. | |
| 511 | Morgan, J. A. T., DeJong, R. J., Jung, Y., Khallaayoune, K., Kock, S., Mkoji, G. M. & Loker, E. S. (2002). A phylogeny of planorbid snails, with implications for the evolution of Schistosoma parasites. *Molecular Phylogenetics and Evolution*, 25, 477-488. | |
| 512 | Morgan, J. A. T., DeJong, R. J., Kazibwe, F., Mkoji, G. M., & Loker, E. S., (2003)a. A newly-identified lineage of *Schistosoma.* *International Journal for Parasitology,* 33(9), 977-985. | |
| 513 | Morgan, J. A. T., DeJong, R. J., Lwambo, N. J. S., Mungai, B. N., Mkoji, G. M., & Loker, E. S., (2003). First Report of a Natural Hybrid between *Schistosoma mansoni* and *S. rodhaini.* *The Journal of Parasitology*, 89(2), 416-418. | |
| 514 | Morgan, J. A. T., DeJong, R. J., Lwambo, N. J. S., Mungai, B. N., Mkoji, G. M., & Loker, E. S., (2003)b. First Report of a Natural Hybrid between Schistosoma mansoni and S. rodhaini. *The Journal of Parasitology,* 89(2), 416-418. | |
| 515 | Morgan, J.A.T., Dejong, R.J., Adeoye, G.O., Ansa, E.D.O., Barbosa, C.S., Brémond, P., Cesari, I.M., Charbonnel, N., Corrêa, I.R., Coulibaly, G., D’andrea, P.S., de souza, C.P., Doenhoff, M.J., File, S., Idris, M.A., Incani, R.N., Jarne, P., Karanja, D.M.S., kazibwe, F., Kpikpi, J., Lwambo, N.J.S., Mabaye, A., Magalhães, L.A., Makundi, A., Moné, H., Mouahid, G., Muchemi, G.M., Mungai, B.N., Séne, M., Southgate, V., Tchuem Tchuenté, L.A., Théron, A., Yousif, F., Zanotti-Magalhães, E.M., Mkoji, G.M., & Loker, E.S., (2005). Origin and diversification of the human parasite Schistosoma mansoni. Molecular Ecology 14(12), 3889-3902. | |
| 516 | Moser, W., Batil, A. A., Ott, R., Abderamane, M., Clements, R., Wampfler, R., Poppert, S., Steinmann, P., Allan, F., & Greter, H., (2022). High prevalence of urinary schistosomiasis in a desert population: results from an exploratory study around the Ounianga lakes in Chad. *Infectious Diseases of Poverty,* 11(1). | |
| 517 | Mostafa, O. M. S., Mossa, A. T. H., & El Einin, H. M. A., (2014). Heavy metal concentrations in the freshwater snail *Biomphalaria alexandrina* uninfected or infected with cercariae of *Schistosoma mansoni* and/or *Echinostoma liei* in Egypt: the potential use of this snail as a bioindicator of pollution. *Journal of Helminthology Cambridge,* 88(4), 411-6. | |
| 518 | Mouchet, F., Rey, J.L., & Cunin, P., (1987). Discovery of *Indoplanorbis-exustus* (Planorbidae, Bulininae) in Yamoussoukro, Ivory-Coast. *Bulletin de la Societe de Pathologie Exotique,* 80(5), 811-812. | |
| 519 | Moyo, N. A. G., (2002). Aspects of the feeding ecology of Sargochromis codringtonii in Lake Kariba, Zimbabwe. *African Journal of Ecology,* 40(3), 241-247. | |
| 520 | Moyroud, J., Breuil, J., & Coulanges, P., (1982). The mollusks intermediate hosts of human bilharziasis in madagascar present state of knowledge. *Archives de l'Institut Pasteur de Madagascar,*50(1), 39-66. | |
| 521 | Mozley, A., (1939). The freshwater Molluscs of the Tanganyika territory and Zanzibar Protectorate, and their relation to human schistosomiasis. *Transactions of the Royal Society of Edinburgh*, 59, 687-744. | |
| 522 | Mudavanhu, A., Schols, R., Goossens, E., Nhiwatiwa, T., Manyangadze, T., Brendonck, L., Huyse, T., (2024). One Health monitoring reveals invasive freshwater snail species, new records, and undescribed parasite diversity in Zimbabwe. *Parasites & Vectors*, 17, 234. https://doi.org/10.1186/s13071-024-06307-4 | |
| 523 | Muhoho, N. D., Katsumata, T., Kimura, E., Migwi, D. K., Mutua, W. R., Kiliku, F. M., Habe, S., & Aoki, Y., (1997). Cercarial density in the river of an endemic area of schistosomiasis haematobia in Kenya. *American Journal of Tropical Medicine and Hygiene,* 57(2), 162-167. | |
| 524 | Mukaratirwa, S., Siegismund, H. R., & Chandiwana, S. K., (1996)a. Mother-offspring data in a study of the mating system in a natural population of Bulinus globosus (Gastropoda: Planorbidae) in Zimbabwe. *Genetical Research,* 68(2), 95-100. | |
| 525 | Mukaratirwa, S., Siegismund, H. R., & Chandiwana, S. K., (1996)b. Population genetics and genetic variability of *Bulinus globosus* (Gastropoda: Planorbidae) from the two main river systems in Zimbabwe. *Journal of Heredity,* 87(4), 288-294. | |
| 526 | Mukaratirwa, S., Siegismund, H. R., Kristensen, T. K., & Chandiwana, S. K., (1996)c. Genetic structure and parasite compatibility of *Bulinus globosus* (Gastropoda: Planorbidae) from two areas of different endemicity of Schistosoma haematobium in Zimbabwe. *International Journal for Parasitology,* 26(3), 269-280. | |
| 527 | Mukaratirwa, S., Munjere, I. F., Takawira, M., & Chingwena, G., (2004). Susceptibility of 7 freshwater gastropod species in Zimbabwe to infection with Gastrodiscus aegyptiacus (Cobbold, 1876) Looss, 1896. *Journal of the South African Veterinary Association,* 75(4), 186-188. | |
| 528 | Mukaratirwa, S., Laidemitt, M. R., Hewitt, R., Sengupta, M. E., Marchi, S., Polius, C., Belmar, S., Scholte, R. G. C., Perez, F., Stensgaard, A-S., Vennervald, B.J., Willingham, A. L., Loker, E. S., (2023). Update on the Geographic Distribution of the Intermediate Host Snails of Schistosoma mansoni on St. Lucia: A Step Toward Confirming the Interruption of Transmission of Human Schistosomiasis. *The American journal of tropical medicine and hygiene*, 109(4), 811-819. https://doi:10.4269/ajtmh.23-0235 | |
| 529 | Mutsaka-Makuvaza, M. J., Zhou, X. N., Tshuma, C., Abe, E., Manasa, J., Manyangadze, T., Allan, F., Chinómbe, N., Webster, B., & Midzi, N., (2020). Molecular diversity of *Bulinus* species in Madziwa area, Shamva district in Zimbabwe: implications for urogenital schistosomiasis transmission. *Parasites & Vectors* 13(1), 1-13. 13. | |
| 530 | Mutuku, M. W., Lu, L., Otiato, F. O., Mwangi, I. N., Kinuthia, J. M, Maina, G. M., Laidemitt, M. R., Lelo, E. A., Ochanda, H., Loker, E. S., & Mkoji, G. M., (2017). A Comparison of Kenyan *Biomphalaria pfeifferi* and B. Sudanica as Vectors for *Schistosoma mansoni*, Including a Discussion of the Need to Better Understand the Effects of Snail Breeding Systems on Transmission. *The Journal of parasitology,* 103 (6), 669-676. | |
| 531 | Muzarabani, K. C., Carolus, H., Schols, R., Hammoud, C., Barson, M., Huyse, T., (2024). An update on snail and trematode communities in the Sanyati Basin of Lake Kariba: New snail and trematode species but no human schistosomes. *Parasitology International,* 99, 102830. https://doi:10.1016/j.parint.2023.102830 | |
| 532 | Nagaty, H. F., (1966). A Preliminary note on some parasites of food-producing animals and their snail intermediate hosts from Sierra Leone. *Veterinary Record,* 78(12), 421-2. | |
| 533 | Nagel, K. O., (1991). On some fresh-water mollusks (Gastropoda and Bivalvia) from Sierra-Leone. *Journal of Conchology* 34, 31-36. | |
| 534 | Nalugwa, A., Jørgensen, A., Nyakaana, S., & Kristensen, T., (2011). Genetic variation within and between populations of hermaphroditic *Bulinus truncatus* tetraploid freshwater snails of the Albertine Rift, East Africa. *Hydrobiologia* 673(1), 53-61. | |
| 535 | Ndamba, J., Chandiwana, S. K., & Makaza, N., (1989). The use of Phytolacca dodecandra berries in the control of trematode-transmitting snails in Zimbabwe. *Acta Tropica,* 46(5–6), 303-309. | |
| 536 | Ndassa, A., & Mimpfoundi, R., (2006). Molecular phylogeny of diploid *Bulinus* sp (Gastropoda: Planorbidae) complex in Cameroon crater lakes*. African Journal of Biotechnology,* 5(2), 113-119. | |
| 537 | Ndassa, A., Mimpfoundi, R., & Elizabeth, M., (2007)a. Molecular phylogeny of diploid *Bulinus* sp (Gastropoda: Planorbidae) populations in Cameroon crater lakes*. Infection Genetics and Evolution,* 7(1), 103-109. | |
| 538 | Ndassa, A., Mimpfoundi, R., Gake, B., Paul Martin, M.V., & Poste, B., (2007)b. Risk factors for human Schistosomiasis in the Upper Benue valley, in northern Cameroon. *Annals of Tropical Medicine & Parasitology,* 101(6), 469-477. | |
| 539 | Ndifon, G. T., & Ukoli, F. M. A., (1989). Ecology of freshwater snails in southern-western Nigeria. 1. Distribution and habitat preference. *Hydrobiologia*, 171, 231-253. | |
| 540 | Ndifon, G. T., & Ukoli, F. M. A., (1989). Ecology of fresh-water snails in southwestern Nigeria .1. distribution and habitat preferences. *Hydrobiologia,* 171(3), 231-253. | |
| 541 | Ndifon, G. T., Betterton, C., & Rollinson, D., (1988). *Schistosonza curassoni* Brumpt, 1931 and *S. bovis* (Sonsino, 1876) in cattle in northern Nigeria. *Journal of Helminthology,* 62(1), 33–34. | |
| 542 | Ndione, R. A., Diop, D., & Jouanard, N., (2018). Role of environmental parameters on the density of intermediate host snails of human *Schistosoma* during the year in the commune of Richard-Toll, Senegal. *Medecine et Sante Tropicales,* 28(2), 158-164. | |
| 543 | Neiber, M. T., Kahl, S. M., & Glaubrecht, M., (2019). Adding the West-African riverine component: Revision of the Recent freshwater snails belonging to Pseudocleopatra Thiele, 1928 (Caenogastropoda, Cerithioidea, Paludomidae). *Zootaxa,* 4674(3), 301-328. | |
| 544 | Nguema, R. M., Langand, J., Galinier, R., Idris, M. A., Shaban, M. A., Yafae, S. A. I., Moné, H., & Mouahid, G., (2013). Genetic diversity, fixation and differentiation of the freshwater snail *Biomphalaria pfeifferi* (Gastropoda, Planorbidae) in arid lands. *Genetica,* 141, 171–184. | |
| 545 | Njiokou, F., Bellec, C., & Jarne, P., (1993). Do self-fertilization and genetic drift promote a very-low genetic-variability in the allotetrapoloid *Bulinus-truncatus* (Gastropoda, Planorbidae) populations. *Genetics Research,* 62(2), 89-100. | |
| 546 | Njiokou, F., Mouafo, J. B., Teukeng, F., Njine, T., Ekobo, A. S., & Jarne, P., (2000). The influence of self-fertilization and pairing on life-history traits in the freshwater snail Bulinus forskalii (Gastropoda, Planorbidae). *Acta Tropica,* 76(2,18), 159-167. | |
| 547 | Njiokou, F., Yimta Tsemo, L. C., & Same Ekobo, A., (2004). Dynamics of intestinal schistosomiasis in Cameroon: evolution of transmission in the mixed zone of Nkolmebanga, Lekie. Medecine tropicale. *Revue du Corps de Sante Colonial,* 64(4), 351-4. | |
| 548 | Nwoko, O. E., Kalinda, C., Manyangadze, T., & Chimbari, M. J., (2022). Species Diversity, Distribution, and Abundance of Freshwater Snails in KwaZulu-Natal, South Africa. *Water,* 14(14), 2267. | |
| 549 | Nwoko, O.E., Manyangadze, T., & Chimbari, M. J., (2023). Spatial and seasonal distribution of human *Schistosomiasis* intermediate host snails and their interactions with other freshwater snails in 7 districts of KwaZulu-Natal province, South Africa. *Scientific Reports,* 13(1), 1-11. | |
| 550 | Nzalawahe, J., Kassuku, A. A., Stothard, J. R., Coles, G. C., & Eisler, M. C., (2015). Associations between trematode infections in cattle and freshwater snails in highland and lowland areas of Iringa Rural District, Tanzania. *Parasitology,* 142(11), 1430-1439. | |
| 551 | Oberholzer, G., & Van Eeden, J. A., (1967). The freshwater molluscs of the Kruger National Park. *Koedoe*, 10, 1-42. | |
| 552 | O'Brien, G. C., Mor, C., Buhl-Nielsen, E., Dickens, Christopher W.S., Olivier, A.-L., Cullis, J., Shrestha, P., Pitts, H., Baleta, H., & Rea, D., (2021). The nature of our mistakes, from promise to practice: water stewardship for sustainable hydropower in Sub-Saharan Africa. River Research and Applications, 37(10), 1538-1547. https://doi.org/10.1002/rra.3849 | |
| 553 | Odeniran, P. O., Omolabi, K. O., & Ademola, I. O., (2020). Economic model of bovine fasciolosis in Nigeria: an update. *Tropical Animal Health and Production,* 52, 3359–3363. | |
| 554 | Odongoaginya, E. I., (1992). A preliminary-study on intermediate snail hosts of Schistosomes in river Enyau, Arua district, Uganda. *East African Medical Journal,* 69(6), 316-318. | |
| 555 | Ofoezie, I. E., (1999). Distribution of freshwater snails in the man-made Oyan Reservoir, Ogun State, Nigeria. *Hydrobiologia,* 416, 181–191. | |
| 556 | Ojo, J. A., Adedokun, S. A., Akindele, A. A., Olorunfemi, A. B., Otutu, O. A., Ojurongbe, T. A., Thomas, B. N., Velavan, T. P., & Ojurongbe, O., (2021). Prevalence of urogenital and intestinal *Schistosomiasis* among school children in South-west Nigeria. *Plos Neglected Tropical Diseases,* 15(7), e0009628. | |
| 557 | Okafor, F. C., (1990)a. Distribution of freshwater gastropods in the lower River Niger and Cross River Basins of southeastern Nigeria with reference to their trematode infections. *Beitraege zur Tropischen Landwirtschaft und Veterinaermedizin,* 28(2), 207-216. | |
| 558 | Okafor, F. C., (1990)b. Schistosoma-haematobium cercariae transmission patterns in freshwater systems of Anambra state Nigeria. *Angewandte Parasitologie,* 31(3):159-166. | |
| 559 | O'Keeffe, J. H., (1985). Population Biology of the Freshwater Snail *Bulinus globosus* on the Kenya Coast. II. Feeding and Density Effects on Population Parameters. *Journal of Applied Ecology,* 22(1), 85-90. | |
| 560 | Okeke, O.C., & Ubachukwu, P.O., (2017). Trematode infections of the freshwater snail *Biomphalaria pfeifferi* from a south-east Nigerian community with emphasis on cercariae of Schistosoma. *Journal of Helminthology* 91(3), 295-301. | |
| 561 | Okoye, C. O., Echude, D., Chiejina, C. O., Andong, F. A., Okoye, K. C., Ugwuja, S. E., Ezeonyejiaku, C. D., & Eyo, J., (2022). Physicochemical Changes and Abundance of Freshwater Snails in Anambra River (Nigeria) During the Rainy Season. *Ecological Chemistry and Engineering,* 29(2), 169-181. | |
| 562 | Oladejo, M. K., Oloyede, O. O., & Morenikeji, O. A., (2021). The abundance, distribution and diversity of invasive and indigenous freshwater snails in a section of the Ogunpa River, southwest Nigeria. *Molluscan Research,* 41(3), 222-234. | |
| 563 | Olkeba, B. K., Boets, P., Mereta, S. T., Mandefro, B. Debesa, G., Ahmednur, M., Ambelu, A., Korma, W., & Goethals, P. L. M., (2022). Malacological and Parasitological Surveys on Ethiopian Rift Valley Lakes: Implications for Control and Elimination of Snail-Borne Diseases. *International Journal of Environmental Research and Public Health,* 19(1), 142. | |
| 564 | Olkeba, B. K., Boets, P., Mereta, S. T., Mesfin, Y., Muleta, A. G., Argaw, A., & Goethals, P. L. M., (2020). Environmental and biotic factors affecting freshwater snail intermediate hosts in the Ethiopian Rift Valley region. *Parasites Vectors,* 13(1), 1-13. | |
| 565 | Olofintoye, L. K., & Odaibo, A. B., (1996). Influence of ecological factors in the population and infection dynamics of *Bulinus globosus* and *Biomethalaria pfeifferi* in the river Odo-Ona, Ibadan, Nigeria. *Helminthologia,* 33(2), 81-86. | |
| 566 | Ololade, I. A., Oladoja, N. A., Ololade, O. O., Saliu, T. D., Alabi, A. B., Obadawo, S. B., & Anifowose, M. M., (2020). Bioaccumulation and toxic potencies of polycyclic aromatic hydrocarbons in freshwater biota from the Ogbese River, Nigeria. *Environmental monitoring and assessment,* 193(1), 8. | |
| 567 | Onabamiro, S. D., (1972). Studies in Schistosomiasis in Sierra Leone .2. seasonal fluctuation in population density of *Bulinus-(Physopsis)-globosus* and *Bulinus-forskalii* in a Schistosomiasis endemic town in Sierra Leone. *Annals of Tropical Medicine and Parasitology,* 66(3), 375. | |
| 568 | Onwona K. M., Peng, F. J., Hogarh, J. N., & Van den Brink, P. J., (2021). Linking Macroinvertebrates and Physicochemical Parameters for Water Quality Assessment in the Lower Basin of the Volta River in Ghana. *Environmental management,* 68(6), 928-936. | |
| 569 | Opara, K. N., Eteye, U. W., Ameh Yaro, C., Alkazmi, L., Udoidung, N. I., Chikezie, F. M., Bassey, B. E., & Batiha, G. E. S., (2021). Prevalence, Risk Factors, and Coinfection of Urogenital Schistosomiasis and Soil-Transmitted Helminthiasis among Primary School Children in Biase, Southern Nigeria. *Journal of Parasitology Research,* 2021(3), 1-12. | |
| 570 | Opisa, S., Odiere, M. R., Jura, W. G. Z. O., Karanja, D. M. S., & Mwinzi, P. N. M., (2011). Malacological survey and geographical distribution of vector snails for schistosomiasis within informal settlements of Kisumu City, western Kenya. *Parasites & Vectors,* 4(1), 226-234. | |
| 571 | Orabi, O., & Khalifa, M. M., (2020). Biota sediment accumulation and bioconcentration factors of trace metals in the snail Melanoides tuberculata form the agricultural drains of the Manzala Lagoon, Egypt. *Environmental Science and Pollution Research,* 27, 17754–17761. | |
| 572 | Ouldabdallahi, M., Ouldbezeid, M., Diop, C., Dem, E., & Lassana, K., (2010). Épidémiologie des bilharzioses humaines en Mauritanie. L’exemple de la rive droite du fleuve Sénégal. *Bulletin de la société de pathologie exotique volume,* 103, 317–322. | |
| 573 | Outa, J. O., & Avenant-Oldewage, A., (2023). Integrated characterisation of Daubaylia burnupiae n. sp. (Nematoda: Daubayliidae) from a freshwater gastropod in South Africa, with comments on the biology of Daubaylia spp. *International Journal For Parasitology-Parasites and Wildlife,* 20, 96-107. | |
| 574 | Outa, J. O., Sattmann, H., Köhsler, M., Walochnik, J., & Jirsa, F., (2020). Diversity of digenean trematode larvae in snails from Lake Victoria, Kenya: First reports and bioindicative aspects. *Acta Tropica,* 206, 105437. | |
| 575 | Owiny, M. O., Obonyo, M. O., Gatongi, P. M., & Fèvre, E. M., (2019). Prevalence and spatial distribution of Trematode cercariae in Vector Snails within different Agro-Ecological Zones in Western Kenya. *Pan African Medical Journal,* 32, 1-12. | |
| 576 | Oyetunde T. O., & Odaibo, A. B., (2012). Preliminary study on ecology of *Bulinus jousseaumei* in *Schistosoma haematobium* endemic rural community of Nigeria. *African Journal of Ecology,* 51(3), 441-446. | |
| 577 | Pacenovsky, J., Zahor, Z., & Krupicer, I., (1987). The 1st finding of Paramphistomum-daubneyi (dinnik, 1962) in cattle in the territory of Algeria. *Veterinarni Medicina,* 32(6), 379-384. | |
| 578 | Palmer, M., Covich, A., Finlay, B., Gibert, J., Hyde, K., Johnson, R., Kairesalo, T., Lake, S., Lovell, C., Naiman, R., Ricci, C., Sabater, F., & Strayer, D., (1997). Biodiversity and ecosystem processes in freshwater sediments. *Ambio*, 26, 571–577. | |
| 579 | Pamba, H. O., & Roberts, J. M. D., (1979). *Schistosomiasis* in and around lake Naivasha, Kenya - 7 years surveillance. *East African Medical Journal,* 56(6), 255-262. | |
| 580 | Pamba, H.O., (1974). *Schistosomiasis* in Nyanza-province-Kenya-1-Rusinga-Island. *East African Medical Journal,* 51(8), 594-599. | |
| 581 | [Papa, F., Creteaux, J. F., Grippa, M., Robert, E., Trigg, M., Tshimanga, R. M., Kitambo, B., Paris, A., Carr, A., Fleischmann, A. S., de Fleury, M., Gbetkom, P. G., Calmettes, B., & Calmant, S., (2023). Water Resources in Africa under Global Change: Monitoring Surface Waters from Space. *Surveys in Geophysics*, 44, 43-93. https://doi.org/10.1007/s1071202209700-9](https://doi.org/10.1007/s1071202209700-9) | |
| 582 | Paperna, I., (1970). Study of an outbreak of schistosomiasis in the newly formed Volta lake in Ghana. *Zeitschrift fur Tropenmedizin und Parasitologie,* 21(4), 411-25. | |
| 583 | Parent, M., & Lietar, J., (1955). Biological studies of mollusca in Jadotville. *Annales de la Societe Belge de Medecine Tropicale,* (1920) 35(1), 59-67. | |
| 584 | Parent, M., (1963). The biology of the mollusks of Jadotville, Katanga. *Bulletin de la Societe de Pathologie Exotique,* 56(2), 189-197. | |
| 585 | [Paugy, D., (2010). An historical review of African freshwater ichthyology. *Freshwater Reviews*, 3, 1-33. https://doi.org/10.1608/FRJ-3.1.1](https://doi.org/10.1608/FRJ-3.1.1) | |
| 586 | Pedersen, U. B., Stendel, M., Midzi, N., Mduluza, T., Soko, W., Stensgaard, A. S., Vennervald, B. J., Mukaratirwa, S., & Kristensen, T.K., (2014). Modelling climate change impact on the spatial distribution of fresh water snails hosting trematodes in Zimbabwe. *Parasites & Vectors,* 7(1), 536. | |
| 587 | Pelseneer, P., (1886). Mollusques recueillis par M. Le Capitaine Storms dans la region du Tanganyika. *Bulletin du Musée Royal d'histoire Naturelle de Belgique*, 4, 103–15. | |
| 588 | Pennance, T., Ame, S. M., Amour, A. K., Suleiman, K. R., Cable, J., & Webster, B. L., (2021). The detection of Schistosoma bovis in livestock on Pemba Island, Zanzibar: A preliminary study. *Current Research in Parasitology & Vector-Borne Diseases,* 1, 100056. | |
| 589 | Pennance, T., Ame, S. M., Amour, A. K., Suleiman, K. R., Muhsin, M. A., Kabole, F., Ali, Said, M., Archer, J., Allan, F., Emery, A., Rabone, M., Knopp, S., Rollinson, D., Cable, J., & Webster, B. L., (2022). Transmission and diversity of Schistosoma haematobium and S. bovis and their freshwater intermediate snail hosts Bulinus globosus and B. nasutus in the Zanzibar Archipelago, United Republic of Tanzania. *PLoS Neglected Tropical Diseases,* 16(7), 1-21. | |
| 590 | Pennance, T., Ame, S. M., Amour, K. A., Khamis, R. S., Allan, F., Rollinson, D., & Webster, B. L., (2018). Occurrence of Schistosoma bovis on Pemba Island, Zanzibar: implications for urogenital *Schistosomiasis* transmission monitoring. *Parasitology,* 145(13), 1727-1731. | |
| 591 | Perez-Saez, J., Mande, T., & Rinaldo, A., (2019). Space and time predictions of schistosomiasis snail host population dynamics across hydrologic regimes in Burkina Faso. *Geospatial Health,* 14(2), 306-313. | |
| 592 | Picquet, M., Ernould, J.C., Vercruysse, J., Southgate, V.R., Mbaye, A., Sambou, B., Niang, M., & Rollinson, D., (1996). The epidemiology of human schistosomiasis in the Senegal River basin. *Transactions of the Royal Society of Tropical Medicine and Hygiene,* 90(4), 340-346. | |
| 593 | Pilsbry, H. A., (1919). A review of the land mollusks of the Belgian Congo chiefly based on the collections of the American Museum Congo Expedition, 1909-1915. *Bulletin of the American Museum of Natural History* 50(i-x), 1-370. | |
| 594 | Pilsbry, H. A., & Bequaert, J., (1927). The aquatic mollusks of the Belgian Congo. With a geographical and ecological account of Congo malacology. *Bulletin of the American Museum of Natural History,* 53, 69–602. | |
| 595 | Pitchford, R. J., (1958)a. Bilharziasis in Swaziland. *The Bulletin of the World Health Organization,* 18(5-6), 735–750. | |
| 596 | Pitchford, R.J., (1958)b. Bilharziasis survey in Bechuanaland. *Bulletin of the World Health Organization,* 18(5-6), 1050–1052. | |
| 597 | Plam, M., Jorgensen, A., Kristensen, T. K., & Madsen, H., (2008). Sympatric *Biomphataria* species (Gastropoda: Planorbidae) in Lake Albert, Uganda, show homoplasies in shell morphology. *African Zoology,* 43(1), 34-44. | |
| 598 | Polderman, A. M., Mpamila, K., & Gryseels, B., (1982). On the distribution and control of Schistosomiasis mansoni in Maniema Zaire. *Acta Leidensia,* 49,17-30. | |
| 599 | Polderman, A. M., Mpamila, K., Manshande, J. P., & Bouwhuishoogerwerf, M. L., (1985). Methodology and interpretation of parasitological surveillance of intestinal *Schistosomiasis* in Maniema, Kivu-Province, Zaire. *Annales de la Societe Belge de Medecine Tropicale,* 65(3), 243-249. | |
| 600 | Pretorius, S. J., Jennings, A. C., Coertze, D. J., & Van Eeden, J. A., (1975). Aspects of the freshwater Mollusca of the Pongola River flood plain pans. *South African Journal of Science,* 71, 208-212. | |
| 601 | Pretorius, S. J., Joubert, P. H., & De Kock, K. N., (1989). A review of the *Schistosomiasis* risk in South African dams. *Water SA,* 15(2), 133-136. | |
| 602 | Pringle, G., Otieno, I. H., & Chimtawi, M. B., (1971). Morphology, susceptibility to *Schistosoma-haematobium* and genetic relationships of *Bulinus-(Physopsis)-globosus-globosus* and b-(p)-nasutus-nasutus from north-eastern Tanzania. *Annals of Tropical Medicine and Parasitology,* 65(2), 211-9. | |
| 603 | Prinsloo, J. F., & Van Eeden, J. A., (1973). The distribution of the freshwater molluscs in Lesotho, with particular reference to the intermediate host of *Fasciola hepatica. Wetenskaplike Bydraes van die Potchefstroomse Universiteit, B, Natuurwetenskappe*, 57, 13 pp. | |
| 604 | Raw, J.L., Rishworth, G.M., Perissinotto, R., & Adams, J.B., (2018). Population fluctuations of *Cerithidea decollata* (Gastropoda: Potamididae) in mangrove habitats of the St Lucia Estuary, South Africa. *African Journal of Marine Science,* 40(4), 461-466. | |
| 605 | Reid, A. J., Carlson, A. K., Creed, I. F., Eliason, E. J., Gell, P. A., Johnson, P. T. J., & Cooke, S. J., (2019). Emerging threats and persistent conservation challenges for freshwater biodiversity. *Biological Reviews*, 94(3), 849–873. | |
| 606 | Ripert, C. L., & Raccurt, C. P., (1987). The impact of small dams on parasitic diseases in Cameroon. *Parasitology Today* 3(9), 287-289. | |
| 607 | Ripert, C., Py, E., (1966). Study of the focus of urinary *Schistosomosis* of Khemis-el-Kachena Algeria human *Schistosoma-haematobium* snails. *Bulletin de la Societe de Pathologie Exotique,* 59(5), 849-859. | |
| 608 | Rollinson, D., & Southgate, V.R., (1979). Enzyme analyses of *Bulinus africanus* group snails (Mollusca: Planorbidae) from Tanzania. *Transactions of the Royal Society of Tropical Medicine and Hygiene,* 73(6), 667-672. | |
| 609 | Rollinson, D., de Clercq, D., Sacko, M., Traoré, M., Sene, M., Southgate, V. R., & Vercruysse, J., (1997). Observations on compatibility between *Bulinus truncatus* and *Schistosoma haematobium* in the Senegal River Basin. *Annals of Tropical Medicine & Parasitology,* 91(4), 371. 8. | |
| 610 | Rollinson, D., Kane, R. A., & Lines, J. R. L., (1989). An analysis of fertilization in *Bulinus-cernicus* (Gastropoda, Planorbidae). *Journal of Zoology,* 217, 295-310. | |
| 611 | Rowel, C., Fred, B., Betson, M., Sousa-Figueiredo, J. C., Kabatereine, N. B., & Stothard, J. R., (2015). Environmental Epidemiology of Intestinal *Schistosomiasis* in Uganda: Population Dynamics of *Biomphalaria* (Gastropoda: Planorbidae) in Lake Albert and Lake Victoria with Observations on Natural Infections with Digenetic Trematodes. *Biomed Research International,* 2015, 717261. | |
| 612 | Sabadini, L., & Marill, F. G., (1953). Urinary bilharziasis in Algeria. *Bulletin de l'Academie Nationale de Medecine,* 137 (30-31), 521-534. | |
| 613 | Sachs, R., & Cumberlidge, N., (1989). Isolation of microcercous cercariae from snails caught in an endemic focus of paragonimus-uterobilateralis in liberia, west-Africa. *Tropical medicine and parasitology,* 40(1), 69-72. | |
| 614 | Saladin, B., Degremont, A., & Weiss, N., (1976). Isoelectric focusing in the taxonomy of Bulinid snails. *Acta Tropica,* 33(4), 376-379. | |
| 615 | Salawu, O. T., & Odaibo, A. B., (2014). The bionomics and diversity of freshwater snails species in Yewa North, Ogun State, Southwestern Nigeria. *Helminthologia,* 51, 337–344. | |
| 616 | Sands, A. F., Riedel, F., Gummersbach, V. S., & Albrecht, C. (2022). Against the Flow: The Colonisation of the Lesotho Highlands by Freshwater Limpets. *Frontiers in Environtal Science* 10, 914272. https://doi:10.3389/fenvs.2022.914272 | |
| 617 | Sama, M. T., & Ratard, R. C., (1994). Water contact and *Schistosomiasis* infection in Kumba, South-Western Cameroon. *Annals of Tropical Medicine and Parasitology,* 88(6), 629-634. | |
| 618 | Saoud, M. F. A., (1966). Susceptibility of some planorbid snails to infection with *Schistosoma rodhaini* from Kenya. *Journal of Helminthology,* 40(3/4), 379-384. | |
| 619 | Sato, M. O., Rafalimanantsoa, A., Ramarokoto, C., Rahetilahy, A. M., Ravaniarimbinina, P., Kawai, S., Minamoto, T., Sato, M., Kirinoki, M., Rasolofo, V., De Calan, M., & Chigusa, Y., (2018). Usefulness of environmental DNA for detecting *Schistosoma mansoni* occurrence sites in Madagascar. *International Journal of Infectious Diseases,* 76, 130–136. | |
| 620 | Saugrain, J., (1968). Malacologic fauna and Bilharzia in the Central African Republic Bromphalaria *Bulinus Schistosoma-mansoni*. *Bulletin de la Societe de Pathologie Exotique,* 61(1), 44-52. | |
| 621 | Sayer, C., Máiz-Tomé, L., Leonard, A., Kishe, M., Natugonza, V., Whitney, C., Omondi, R., Nshutiyayesu, S., & Kabuye, C., (2018). The importance of freshwater species to livelihoods in the Lake Victoria Basin. In: C. A. Sayer, L. Máiz-Tomé, & W. R. T. Darwall (Eds.), *Freshwater biodiversity in the Lake Victoria Basin: Guidance for species conservation, site protection, climate resilience and sustainable livelihoods* (pp. 136–151). International Union for the Conservation of Nature. | |
| 622 | Schols, R., Mudavanhu, A., Carolus, H., Hammoud, C., Muzarabani, K. C., Barson, M., Huyse, T., (2020). Exposing the Barcoding Void: An Integrative Approach to Study Snail-Borne Parasites in a One Health Context. *Frontiers in Veterinary Science* 7, 605280. https://doi:10.3389/fvets.2020.60528 | |
| 623 | Schols, R., Carolus, H., Hammoud, C., Muzarabani, K.C., Barson, M., & Huyse, T., (2021). Invasive snails, parasite spillback, and potential parasite spillover drive parasitic diseases of Hippopotamus amphibius in artificial lakes of Zimbabwe. *BMC Biology,* 19(1). | |
| 624 | Schols, R., Vanoverberghe, I., Huyse, T., & Decaestecker, E., (2023). Host-bacteriome transplants of the schistosome snail host Biomphalaria glabrata reflect species-specific associations. *FEMS Microbiology Ecology*, 99, 1–9. https://DOI:10.1093/femsec/fiad101 | |
| 625 | Schultheiß, R., Van Bocxlaer, B., Wilke, T. & Albrecht, C., (2009). Old fossils–young species: evolutionary history of an endemic gastropod assemblage in Lake Malawi. *Proceedings of the Royal Society*, B, 276, 2837-2846. | |
| 626 | Schultheiß, R. Wilke, T., Jørgensen & Albrecht, C., (2011). The birth of an endemic species flock: demographic history of the Bellamya group (Gastropoda, Viviparidae) in Lake Malawi. *Biological Journal of the Linnean Society,* 102, 130-143. | |
| 627 | Schultheiß, R., Van Bocxlaer, B., Riedel, F., von Rintelen, T., & Albrecht, C., (2014). Disjunct distributions of freshwater snails testify to a central role of the Congo system in shaping biogeographical patterns in Africa. *Evolutionary Biology,* 14(1), 1-23. | |
| 628 | Schutte, C. H. J., Evans, A. C., & Joubert, J. J., (1995). Epidemiology and control of schistosomiasis mansoni in communities living on the Cuando River floodplain of East Caprivi, Namibia. *Annals of Tropical Medicine and Parasitology,* 89(6), 631-644. | |
| 629 | Schutte, C.H.J., & Frank, G.H., (1964). Observations on the distribution of freshwater Mollusca and chemistry of the natural waters in the South-eastern Transvaal and adjacent Northern Swaziland. *Bulletin of the World Health Organisation* 30, 389-400. | |
| 630 | Schwetz, J., & Dartevelle, E., (1948). Sur l’origine des mollusques thalassoides du Lac Tanganyika. Revue historique et analytique. *Memoires de la Institut Royal Colonial Belge, Sciences Naturelles et Medicales,* 16(7), 1–58. | |
| 631 | Schwetz, J., (1950). Sur le transmission de *Schistosoma mansoni* par les plan-orbes fluviatiles du Congo oriental (troisieme etude). *Annales de la Societe Belge de Medecine Tropicale,* 30, 585-593. | |
| 632 | Schwetz, J., (1951)a. Preliminary note on bilharziosis at Sakania (Katanga, Belgian Congo). *Annales de la Societe belge de Medecine Tropicale,* (1920) 31(1), 93-102. | |
| 633 | Schwetz, J., (1951)b. Sur le probleme actuel des bilharzioses humaines au Congo beige. *Bulletin de la Societe de Pathologie Exotique,* 44(3-4), 195-202. | |
| 634 | Schwetz, J., (1952)a. A third focus of *Schistosoma rodhaini* Brumpt in the Belgian Congo. *Annales de la Societe Belge de Medecine Tropicale,* (1920) 32(6), 673-7. | |
| 635 | Schwetz, J., (1952)b. Planorbis tanganikanus; Planorbis tanganyicensis du lac Tanganika est transmetteur de Schistosoma mansoni. *Annales de la Societe belge de medecine tropicale,* (1920) 32(6), 665-71. | |
| 636 | Schwetz, J., (1953). On the causes of confusion in the nomenclature of the mollusks which transmit schistosomiasis in Ethiopian Africa and the necessity and means of ending it. *Bulletin de la Société de pathologie exotique,* 46(5), 765-783. | |
| 637 | Schwetz, J., (1954)a. Observations on the comparative behavior of the planor-bid-*Schistosoma mansoni* and *Physopsis-S. haematobium* complexes in separate or mixed foci of *S. mansoni and S. haematobium.* *Bulletin de la Societe de Pathologie Exotique,*47(2), 332-338. | |
| 638 | Schwetz, J., (1954)b. On two schistosomes of wild rodents of the Belgian Congo: *Schistosoma rodhaini* Brumpt, 1931; and *Schistosoma mansoni* var. Rodentorum Schwetz, 1953a, Schwetz, 1953b; and their relationship to *S. mansoni* of man. *Transactions of the Royal Society of Tropical Medicine and Hygiene,* 48(1), 89-92. | |
| 639 | Schwetz, J., (1956)a. Demonstration sur une collection de mollusques, varies, africains, lacustres et fluviatiles, hotes intermediaires de schistosomes humains et animaux; presentation de la collection. *Bulletin de la Societe de pathologie exotique et de ses filiales,* 49(6), 1178-94. | |
| 640 | Schwetz, J., (1956)b. The effect of climatic variations on pulmonate gastropoda, compared with that of molluscicides. *Transactions of the Royal Society of Tropical Medicine and Hygiene,* 50(6), 579-582. | |
| 641 | Seddon, M., Appleton, C. C., Van Damme, D., & Graf, D., (2011*).* Freshwater molluscs of Africa: diversity, distribution, and conservation*.* In: Darwall, W., Smith, K.; Allen, D.; Holland, R; Harrison, I and Brooks, E (Eds), *The diversity of life in African freshwaters: underwater, under threat. An analysis of the status and distribution of freshwater species throughout mainland Africa.* IUCN, Gland, Switzerland: 92–125. | |
| 642 | Sellin, B., Simonkovich, E., & Roux, J., (1980). Etude de la repartition des mollusques, hotes intermediaires des schistosomes en Afrique de l'ouest. Premiers resultats. *Medecine Tropicale,* 40(1), 31-39. | |
| 643 | Senghor, B., Diaw, O. T., Doucoure, S., Seye, M., Talla, I., Diallo, A., Bâ, C. T., & Sokhna, C., (2015). Study of the snail intermediate hosts of urogenital schistosomiasis in Niakhar, region of Fatick, West central Senegal. *Parasites & Vectors,* 8(1), 1-8. | |
| 644 | Sengupta, M. E., Kristensen, T. K., Madsen, H., & Jorgensen, A., (2009). Molecular phylogenetic investigations of the Viviparidae (Gastropoda: Caenogastropoda) in the lakes of the Rift Valley area of Africa. *Molecular Phylogenetics and Evolution,* 52(3), 797-805. | |
| 645 | [Shiff, C., (2017). Why Reinvent the Wheel? Lessons in *Schistosomiasis* Control from the Past. *PLoS Neglected Tropical Diseases*, 11(10), e0005812. https://doi.org/10.1371/journal.pntd.0005812](https://doi.org/10.1371/journal.pntd.0005812) | |
| 646 | Shift, C.J., (1960). Observations on the capability of freshwater vector snails to survive dry conditions. *Journal of Tropical Medicine and Hygiene,*63, 89-93. | |
| 647 | [Shivambu, T. C., Shivambu, N., & Downs, C. T., (2020). Exotic gastropods for sale: an assessment of land and aquatic snails in the South African pet trade. *Management of Biological Invasions*, 11(3), 512–524. https://doi.org/10.3391/mbi.2020.11.3.1](https://doi.org/10.3391/mbi.2020.11.3.1) | |
| 648 | Shoukry, Nahla M., El-Assal, Faiza M., Mansour, & Noshy S., (1997). Susceptibility of three successive snail generations from positive and negative laboratory bred *Biomphalaria alexandrina* from different localities in Egypt to infection with *Schistosoma mansoni* from Giza. *Journal of the Egyptian Society of Parasitology,* 27(2), 317-329. | |
| 649 | Sijun, L., Si-Ming Zhang, S. K., Buddenborg, E. S., & Loker, B. C. B., (2021). Virus-derived sequences from the transcriptomes of two snail vectors of schistosomiasis, *Biomphalaria pfeifferi* and *Bulinus globosus* from Kenya. *PeerJ* 9. | |
| 650 | [Simaika, J. P., Chakona, A., & van Dam, A. A., (2021). Editorial: towards the sustainable use of African Wetlands. Frontiers in Environmental Science, 9, 658871. https://doi.org/10.3389/fenvs.2021.658871](https://doi.org/10.3389/fenvs.2021.658871) | |
| 651 | Simonet, P., (1952). Observations nouvelles sur la pathologie des indigenes dans l'annexe de la Savura (Beni Abbes, Sud Orandis). *Archives de l'Institut Pasteur d'Algérie,* 30(2), 134-145. | |
| 652 | Sitnikova, T. Ya., (2021). New Data on the Reproductive System of Syrnolopsis minuta Bourguignat 1885 (Gastropoda, Cerithioidea) from Lake Tanganyika, Africa. *Biology Bulletin,* 48, 1189-1192. | |
| 653 | Siwela, A. H., Nyathi, C. B., & Naik, Y. S., (2010). A comparison of metal levels and antioxidant enzymes in freshwater snails, *Lymnaea natalensis*, exposed to sediment and water collected from Wright Dam and Lower Mguza Dam, Bulawayo, Zimbabwe. *Ecotoxicology and Environmental Safety,* 73(7), 1728-1732. | |
| 654 | Skowno, A. L., Poole, C. J., Raimondo, D. C., Sink, K. J., Van Deventer, H., Van Niekerk, L., Harris, L. R., Smith Adao, L. B., Tolley, K. A., Zengeya, T.A., Foden, W. B., Midgley, G. F., & Driver, A., (2019). *National Biodiversity Assessment 2018*: The status of South Africa’s ecosystems and biodiversity. Synthesis Report. South African National Biodiversity Institute, an entity of the Department of Environment, Forestry and Fisheries, Pretoria. pp. 1–214. | |
| 655 | Smith, E. A., (1880)a. Diagnoses of new shells from Lake Tanganyika and East Africa. *Annals and Magazine of Natural History*, 6, 425–430. | |
| 656 | Smith, E. A., (1880)b. On the shells of Lake Tanganyika and of the neighbourhood of Ujiji, Central Africa. *Proceedings of the Zoological Society of London*, 1880, 344–352. | |
| 657 | Smith, E. A., (1881). Descriptions of two new species of shells from Lake Tanganyika. *Proceedings of the Zoological Society of London*, 49(3), 558–561. | |
| 658 | Smith, E. A., (1889). Diagnosis of new shells from Lake Tanganyika. *Annals and Magazine of Natural History*, 6,173–175. | |
| 659 | Smith, E. A., (1904). Some remarks on the Mollusca of Lake Tanganyika. *Proceedings of the Malacological Society of London,* 6(2), 77–104. | |
| 660 | Smith, V. G. F., (1982). Distribution of snails of medical and veterinary importance in an organically polluted watercourse in Nigeria. *Annals of Tropical Medicine and Parasitology,* 76(5):539-546. | |
| 661 | Smithers, S. R., (1956). On the ecology of schistosome vectors in the Gambia, with evidence of their rôle in transmission. *Transactions of the Royal Society of Tropical Medicine and Hygiene,* 50(4), 354-358. | |
| 662 | Smithers, S. R., (1957). The occurrence of *Schistosoma mansoni* in the Gambia. *Annals of Tropical Medicine and Parasitology,*51, 359-363. | |
| 663 | Smithers, S. R., (1958). Attempted control of Bulinus senegalensis Muller, a vector of Schistosoma haematobium in the Gambia. *Annals of Tropical Medicine & Parasitology,* 52(3), 315-319. | |
| 664 | Sodeman, W. A., (1972). The Reaction of Schistosome Vector Snails to Direct Current. *The Journal of Parasitology,* 58(4), 842-843. | |
| 665 | Sodeman, W. A., (1973). Distribution of Schistosome vector snails in central Liberia. *Annals of tropical medicine and parasitology,* 67(3), 357-365. | |
| 666 | Sodeman, W. A., (1979). Longitudinal-study of Schistosome vector snail populations in Liberia. *American journal of tropical medicine and hygiene,* 28(3), 531-538. | |
| 667 | Sodeman, W. A., Rodrick, G. E., & Vincent, A. L., (1980). Lampyridae larva - a natural predator of Schistosome vector snails in Liberia. *American journal of tropical medicine and hygiene* 29(2), 319-319. | |
| 668 | Sorensen, L. V., Jorgensen, A., & Kristensen, T. K., (2005). Molecular diversity and phylogenetic relationships of the gastropod genus Melanoides in Lake Malawi. *African Zoology,* 40(2), 179-191. | |
| 669 | Sourrouille, P., Debain, C., & Jarne, P., (2002). Microsatellite variation in the freshwater snail Physa acuta. *Molecular Ecology Notes,* 3(1), 21-23. | |
| 670 | Southgate, V. R., & Knowles, R. J., (1975). The intermediate hosts of Schistosoma bovis in Western Kenya. *Transactions of the Royal Society of Tropical Medicine and Hygiene,* 69(3), 356-357. | |
| 671 | Southgate, V. R., & Knowles, R. J., (1977)a. On the intermediate hosts of Schistosoma haematobium from Western Kenya. *Transactions of the Royal Society of Tropical Medicine and Hygiene,* 71(1), 82-83. | |
| 672 | Southgate, V. R., & Knowles, R. J., (1977)b. Schistosoma-margrebowiei-Leroux, 1933 - morphology of egg, miracidium and cercaria, compatibility with species of bulinus, and development in Mesocricetus-auratus. *Zeitschrift fur Parasitenkunde-Parasitology Research,* 54(3), 233-250. | |
| 673 | Southgate, V. R., (1997). *Schistosomiasis* in the Senegal river basin: Before and after the construction of the dams at Diama, Senegal and Manantali, Mali and future prospects. *Journal of Helminthology,* 71(2), 125-132. | |
| 674 | Standley, C. J., & Stothard, J. R., (2012). DNA Barcoding of Schistosome Cercariae Reveals a Novel Sub-Lineage within *Schistosoma rodhaini* From Ngamba Island Chimpanzee Sanctuary, Lake Victoria. *Journal of Parasitology,* 98(5), 1049-1051. | |
| 675 | Standley, C. J., Goodacre, S. L., Wade, C. M., & Stothard, J. R., (2014). The population genetic structure of *Biomphalaria choanomphala* in Lake Victoria, East Africa: implications for *Schistosomiasis* transmission. *Parasites & Vectors,* 7, 524. | |
| 676 | Standley, C. J., Vounatsou, P., Gosoniu, L., Mckeon, C., Adriko, M., Kabatereine, N. B., & Stothard, J. R., (2013). Micro-scale investigation of intestinal schistosomiasis transmission on Ngamba and Kimi islands, Lake Victoria, Uganda. *Acta Tropica,* 128(2), 353-364. | |
| 677 | Standley, C. J., Vounatsou, P., Gosoniu, L., Jorgensen, A., Adriko, M., Lwambo, N. J. S., Lange, C. N., Kabatereine, N. B., & Stothard, J. R., (2012). The distribution of *Biomphalaria* (Gastropoda: Planorbidae) in Lake Victoria with ecological and spatial predictions using Bayesian modelling. *Hydrobiologia,* 683(1), 249-264. | |
| 678 | Standley, C. J., Wade, C. M., & Stothard, J. R., (2011). A Fresh Insight into Transmission of *Schistosomiasis*: A Misleading Tale of *Biomphalaria* in Lake Victoria. *PlosOne,* 6(10),e26563. | |
| 679 | Starmühlner, F., (1969). Die Gastropoden des Madagassischen Binnengewässer. *Malacologia*, 8, 1-434. | |
| 680 | Starmühlner, F., (1976)a. Contribution to the knowledge of the freshwater fauna of the Isle of Anjouan (Comores). *Cashiers de l’Office de la Recherche Scientifique et Technique Outre-Mer, série Hydrobiologie,* 10, 255-265. | |
| 681 | Starmühlner, F., (1976)b. Contribution to the knowledge of the freshwater fauna of running waters of Mauritius. *Bulletin of the Mauritius Institute*, 8, 105-128. | |
| 682 | Starmühlner, F., (1977). Contribution to the knowledge of the freshwater fauna of La Reunion (Mascarene). *Cashiers de l’Office de la Recherche Scientifique et Technique Outre-Mer, série Hydrobiologie*, 11, 239-250. | |
| 683 | Starmühlner, F., (1983). Results of the Hydrobiological Mission 1974 of the Zoological Institute of the University of Vienna. 8. Contributions to the knowledge of the freshwater gastropods of the Indian Ocean Islands (Seychelles, Comores, Mascarene Archipelagos). *Annalen des Naturhistorischen Museums, Wien*, 84B, 127-249. | |
| 684 | Stauffer, J. R., Arnegard, M. E., Cetron, M., Sullivan, J. J., Chitsulo, L. A., Turner, G. F., Chiotha, S., & McKaye, K. R., (1997). Controlling vectors and hosts of parasitic diseases using fishes - A case history of schistosomiasis in Lake Malawi. *Bioscience,* 47(1), 41-49. | |
| 685 | Stauffer, J. R., Madsen, H., & Rollinson, D., (2014). Introgression in Lake Malawi: Increasing the Threat of Human Urogenital Schistosomiais? *Ecohealth,* 11(2), 251-254. | |
| 686 | Stauffer, J. R., Madsen, H., Konings, A., Bloch, P., Ferreri, C. P., Likongwe, J., McKaye, K. R., & Black, K. E., (2007). Taxonomy: A Precursor to Understanding Ecological Interactions among Schistosomes, Snail Hosts, and Snail-Eating Fishes. *Transactions of the american fisheries society,* 136(4), 1136-1145. | |
| 687 | Stauffer, J. R., Madsen, H., McKaye, K., Konings, A., Bloch, P., Ferreri, C. P., Likongwe, & Makaula, P., (2006). *Schistosomiasis* in Lake Malawi: Relationship of fish and intermediate host density to prevalence of human infection. *Ecohealth,* 3(1), 22-27. | |
| 688 | Stauffer, J. R., Madsen, H., Webster, B., Black, K., Rollinson, D., & Konings, A., (2008). Schistosoma haematobium in Lake Malawi: susceptibility and molecular diversity of the snail hosts Bulinus globous and B. nyassanus. *Journal of Helminthology,* 82(4), 377-382. | |
| 689 | Steiner, F., Ignatius, R., Friedrich-Jaenicke, B., Dieckmann, S., Harms, G., Poppert, S., & Mockenhaupt, F.P., (2013). Acute Schistosomiasis in European Students Returning From Fieldwork at Lake Tanganyika, Tanzania. *Journal of Travel Medicine,* 20(6), 380-383. | |
| 690 | Stensgaard, A.S., Jorgensen, A., Kabatereine, N.B., Rahbek, C., & Kristensen, T.K., (2006). Modeling freshwater snail habitat suitability and areas of potential snail-borne disease transmission in Uganda. *Geospatial Health,* 1(1), 93-104. | |
| 691 | Stothard, J. R., & Rollinson, D., (1997). Partial DNA sequences from the mitochondrial cytochrome oxidase subunit I (COI) gene can differentiate the intermediate snail hosts *Bulinus globosus* and *B-nasutus* (Gastropoda: Planorbidae). *Journal of Natural History,* 31(5), 727-737. | |
| 692 | Stothard, J. R., Ameri, H., Khamis, I. S., Blair, L., Nyandindi, U. S., Kane, R. A., Johnston, D. A., Webster, B. L., & Rollinson, D., (2013). Parasitological and malacological surveys reveal urogenital *Schistosomiasis* on Mafia Island, Tanzania to be an imported infection. *Acta Tropica,* 128(2), 326-333. | |
| 693 | Stothard, J. R., Brémond, P., Andriamaro, L., Loxton, N. J., Sellin, B., Sellin, E., & Rollinson, D., (2000). Molecular characterization of the freshwater snail Lymnaea natalensis (Gastropoda : Lymnaeidae) on Madagascar with an observation of an unusual polymorphism in ribosomal small subunit genes. *Journal of Zoology,* 252, 303-315. | |
| 694 | Stothard, J. R., Loxton, N. J., & Rollinson, D., (2006). Freshwater snails on Mafia Island, Tanzania with special emphasis upon the genus *Bulinus* (Gastropoda: Planorbidae). *Journal of Zoology,* 257(3), 353-364. | |
| 695 | Stothard, J. R., Loxton, N., Rollinson, D., Mgeni, A. F., Khamis, S., Ameri, H., Ramsan, M., & Saviolo, L., (2000). The transmission status of Bulinus on Zanzibar Island (Unguja), with implications for control of urinary schistosomiasis. *Annals of Tropical Medicine & Parasitology,* 94(1), 87-94. | |
| 696 | Stuart, S. N., Richard, J. A., & Jenkins, M., (1990). *Biodiversity in Sub-Saharan Africa and Its Islands:* Conservation, Management, and Sustainable Use. Publisher, IUCN – The World Conservation Union. ISBN 2-8317-0021-3 | |
| 697 | Sturany, R., (1898). Catalog der bisher bekannt gewordenen Südafrikanischen Land- und Süsswasser-Mollusken mit besonderer Berücksichtigung des von Dr. Penther gesammelten Materiales. *Denkschriften der Mathematisch-Naturwissenschaftlichen Classe der Kaiserlichen Akademie für Wissenschaften,* 67, 537-642. | |
| 698 | Sturrock, R. F., Karamsadkar, S. J., & Ouma, J., (1979). Schistosome infection-rates in field snails - schistosoma-mansoni in biomphalaria-pfeifferi from kenya. *Annals of tropical medicine and parasitology,* 73(4), 369-375. | |
| 699 | Tabetderraz, O., Belkaid, M., & Ouchfoun, A. (1977). A new site of urinary Bilharziasis in Algeria. *Bulletin de la Societe de Pathologie Exotique,* 70 (3), 224-227. | |
| 700 | [Tabo, Z., Neubauer, T. A., Tumwebaze, I., Stelbrink, B., Breuer, L., Hammoud, C., & Albrecht, C., (2022). Factors controlling the distribution of intermediate host snails of Schistosoma in Crater Lakes in Uganda: A machine learning approach. *Frontiers in Environmental Science*, 10, 871735. https://doi.org/10.3389/fenvs.2022.871735](https://doi.org/10.3389/fenvs.2022.871735) | |
| 701 | Tabo, Z., Breuer, L., Fabia, C., Samuel, G., & Albrecht, C., (2024). A machine learning approach for modeling the occurrence of the major intermediate hosts for *Schistosomiasis* in East Africa. *Scientific Reports,* 14, 4274 https://doi.org/10.1038/s41598-024-54699-1 | |
| 702 | Takahashi, T., Watanabe, K., Munehara, H., Rüber, L., & Hori, M., (2009). Evidence for divergent natural selection of a Lake Tanganyika cichlid inferred from repeated radiations in body size. *Molecular Ecology,* 18(14), 3110-3119. | |
| 703 | Takougang, I., Barbazan, P., Tchounwou, P. B., & Noumi, E., (2008). The value of the freshwater snail dip scoop sampling method in macroinvertebrates bioassessment of sugar mill wastewater pollution in Mbandjock, Cameroon. *International Journal of Environmental Research and Public Health,* 5(1), 68-75. | |
| 704 | Takougang, I., Ekobo, A.S., & Enyong, P., (1994). Ecological impact of the proposed hydroelectric dam of Memveele (Cameroon) on vector-borne diseases. *Bulletin de la Societe de Pathologie Exotique,* 87(4), 261-266. | |
| 705 | Takougang, I., Meli, J., Wabo Poné, J., & Angwafo, F., (2007). Community acceptability of the use of low-dose niclosamide (Bayluscide®), as a molluscicide in the control of human *Schistosomiasis* in Sahelian Cameroon. *Annals of Tropical Medicine & Parasitology,* 101(6), 479-486. | |
| 706 | Tandina, F., Doumbo, S. N., & Doumbo, O.K., (2016). Epidemiology of *Schistosomiasis* in the periurban area of Sotuba, 10 years mass treatment began in Mali. *Medecine et sante tropicales,*26(1), 51-56. | |
| 707 | Taybi, A. F., Gloer, P., & Mabrouki, Y., (2023). A new genus and new species of springsnails (Gastropoda, Hydrobiidae) from north-East Morocco. *Nature Conservation Research,* 8(3). | |
| 708 | Tchakonte, S., Nana, P. A., Tamsa, A. A., Tchatcho, N. L. N. T., Koji, E., Onana, F. M., & Ajeagah, G. A., (2023). Using machine learning models to assess the population dynamic of the freshwater invasive snail *Physa acuta* Draparnaud, 1805 (Gastropoda: Physidae) in a tropical urban polluted streams-system. *Limnologica*, 99, 126049. | |
| 709 | Tchernov, E., (1971). Freshwater molluscs of the Sinai Peninsula. *Israel Journal of Zoology,* 20, 209-221. | |
| 710 | Tchounwou, P. B., Englande, A. J., & Malek, E. A., (1991)a. Toxicity evaluation of ammonium-sulfate and urea to 3 developmental stages of fresh-water snails. *Archives of Environmental Contamination and Toxicology,* 21(3), 359-364. | |
| 711 | Tchounwou, P. B., Englande, A. J., & Malek, E. A., (1991)b. Toxicity evaluation of Bayluscide and Malathion to 3 developmental stages of fresh-water snails. *Archives of Environmental Contamination and Toxicology,* 21(3), 351-358. | |
| 712 | Teesdale, C., (1962). Ecological observations on the molluscs of significance in the transmission of bilharziasis in Kenya. *Bulletin of the World Health Organization,* 27(6), 759–782. | |
| 713 | Tian-Bi, Y. N. T., Konan, J. N. K., Sangaré, A., Ortega-Abboud, E., Utzinger, J., N’Goran, E. K., & Jarne, P., (2019)a. Spatio-temporal population genetic structure, relative to demographic and ecological characteristics, in the freshwater snail *Biomphalaria pfeifferi* in Man, western Côte d’Ivoire. *Genetica,* 147, 33–45. | |
| 714 | Tian-Bi, Y. N. T., Webster, B., Konan, C. K., Allan, F., Diakité, N. R., Ouattara, M., Salia, D., Koné, A., Kakou, A. K., Rabone, M., Coulibaly, J. T., Knopp, S., Meïté, A., Utzinger, J., N'Goran, E. K., & Rollinson, D., (2019)b. Molecular characterization and distribution of Schistosoma cercariae collected from naturally infected bulinid snails in northern and central Côte d'Ivoire. *Parasites Vectors,* 12(1), 117. | |
| 715 | Trienekens, S. C. M., Faust, C. L., Besigye, F., Pickering, L., Tukahebwa, E. M., Seeley, J., & Lamberton, P. H. L., (2022). Variation in water contact behaviour and risk of *Schistosoma mansoni* (re)infection among Ugandan school-aged children in an area with persistent high endemicity. *Parasites & Vectors,* 15(1). | |
| 716 | [Tumwebaze, I., Clewing, C., Chibwana, F. D., Kipyegon, J. K., & Albrecht, C., (2022). Evolution and Biogeography of Freshwater Snails of the Genus *Bulinus* (Gastropoda) in Afromontane Extreme Environments. *Frontiers in Environmental Science,* 10. https://doi.org/10.3389/fenvs.2022.902900](https://doi.org/10.3389/fenvs.2022.902900) | |
| 717 | Upatham, E.S., koura, M., & Awad, A.H., (1981). Studies on the transmission of *Schistosoma-haematobium* and the bionomics of *Bulinus* (ph) abyssinicus in the Somali-Democratic Republic. *Annals of Tropical Medicine and Parasitology,* 75(1), 63-69. | |
| 718 | Utzinger, J., & Tanner, M., (2000). Microhabitat Preferences of Biomphalaria pfeifferi and Lymnaea natalensis in a Natural and a Man-made Habitat in Southeastern Tanzania. *Memórias do Instituto Oswaldo Cruz,* 95(3), 287-294. | |
| 719 | Utzinger, J., Mayombana, C., Mez, K., & Tanner, M., (1997)a. Evaluation of chemical and physical-morphological factors as potential determinants of *Biomphalaria pfeifferi* (Krauss, 1848) distribution. *Memorias Do Instituto Oswaldo Cruz* 92, (3), 323-328. | |
| 720 | Utzinger, J., Mayombana, C., Smith, T., & Tanner, M., (1997)b.  Spatial microhabitat selection by *Biomphalaria pfeifferi* in a small perennial river in Tanzania. *Hydrobiologia,* 356(1-3), 53-60. | |
| 721 | [Utzinger, J., Raso, G., Brooker, S., De Savigny, D., Tanner, M., Ørnbjerg, N., Singer, B. H., & N’goran, E. K., (2009). *Schistosomiasis* and neglected tropical diseases: towards integrated and sustainable control and a word of caution. *Parasitology,* 136(13), 1859–1874. https://doi.org/10.1017/s0031182009991600](https://doi.org/10.1017/s0031182009991600) | |
| 722 | Van Bocxlaer, B., (2005). Changes in the malacofauna of Lake Malawi since mid-Holocene times. *Geologica Belgica,* 8 (3), 124. | |
| 723 | Van Bocxlaer, B., (2020). Paleoecological insights from fossil freshwater mollusks of the Kanapoi Formation (Omo-Turkana Basin, Kenya). *Journal of Human Evolution,* 140, 102341. | |
| 724 | Van Bocxlaer, B., & Albrecht, C., (2015). Ecosystem change and establishment of an invasive snail alter gastropod communities in long-lived Lake Malawi. *Hydrobiologia,* 744(1), 307-316. | |
| 725 | Van Bocxlaer, B. & Van Damme, D., (2009). Palaeobiology and evolution of the Late Cenozoic freshwater molluscs of the Turkana Basin: Iridinidae Swainson, 1840 and Etheriidae Deshayes, 1830 (Bivalvia: Etheroidea). *Journal of Systematic Palaeontology,* 7 (2), 129-161. | |
| 726 | Van Bocxlaer, B., Van Damme, D. & Feibel, C. S., (2008). Gradual versus punctuated equilibrium evolution in the Turkana Basin molluscs: Evolutionary events or Biological Invasions? *Evolution,* 62(3), 511-520. | |
| 727 | Van Bocxlaer, B., Verschuren, D., Schettler, G., & Kröpelin, S., (2011). Modern and early Holocene mollusc fauna of the Ounianga lakes (northern Chad): implications for the palaeohydrology of the central Sahara. *Journal of Quaternary Science,* 26(4), 433-447. | |
| 728 | Van Bocxlaer, B., Schultheiß, R., Plisnier, P.-D., & Albrecht, C. (2012). Does the decline of gastropods in deep water herald ecosystem change in Lakes Malawi and Tanganyika? Freshwater Biology, 57, 1733–1744. https://doi:10.1111/j.1365-2427.2012.02828.x | |
| 729 | Van Bocxlaer, B., Ortiz-Sepulveda, C. M., Gurdebeke, P. R., & Vekemans, X., (2020). Adaptive divergence in shell morphology in an ongoing gastropod radiation from Lake Malawi. *BMC Evolutionary Biology,* 20(1), DOI10.1186/s12862-019-1570-5 | |
| 730 | Van Bocxlaer, B., Clewing, C., Etimosundja, J. P. M., Kankonda, A., Wembo Ndeo, O., & Albrecht, C., (2015). Recurrent camouflaged invasions and dispersal of an Asian freshwater gastropod in tropical Africa. *Evolutionary Biology,* 15 (1), 1-18. | |
| 731 | Van Damme, D. & Pickford, M., (1999). The Late Cenozoic Viviparidae (Mollusca, Gastropoda) of the Albertine Rift Valley (Uganda-Zaire). *Hydrobiologia,* 390, 171 217 | |
| 732 | Van Damme, D. & Van Bocxlaer, B., (2009). Freshwater molluscs of the Nile Basin, past and present. Pp. 585-630, in: Dumont, H. J. (Ed.) The Nile: Origin, environment, limnology and human use. Monographiae Biologicae. Springer Verlag: Dordrecht, Netherlands. | |
| 733 | Van Damme, D., & Pickford, M., (1995). The late Cenozoic ampullariidae (mollusca, gastropoda) of the Albertine Rift Valley (Uganda-Zaire). Hydrobiologia 316(1), 1-32. | |
| 734 | Van Damme, D., & Pickford, M., (2003). The late Cenozoic Thiaridae (Mollusca, Gastropoda, Cerithioidea) of the Albertine Rift Valley (Uganda-Congo) and their bearing on the origin and evolution of the Tanganyikan thalassoid malacofauna. Hydrobiologia 498(1-3), 1-83. | |
| 735 | Van Damme, D., (1984). *Freshwater Mollusca of Northern Africa. Developments in Hydrobiology* 25, Pp. i-xii, 1-164. Dr. W. Junk Publishers: Dordrecht, Netherlands. | |
| 736 | van der Deure, T., Maes, T., Huyse, T., & Stensgaard, A.-S. (2024). Climate change could fuel urinary schistosomiasis transmission in Africa and Europe. *Global Change Biology*, 30, e17434. https://doi.org/10.1111/gcb.17434 | |
| 737 | Van Eeden, J. A., & Brown, D. S., (1966). Colonization of Fresh Waters in the Republic of South Africa by *Lymnaea columella* Say (Mollusca : Gastropoda). *Nature volume,* 210, 1172–1173. | |
| 738 | Van Eeden, J. A., & Combrinck, C., (1966). Distribution trends of four species of freshwater snails in South Africa, with special reference to the intermediate hosts of bilharzia. *Zoologica Africana*, 2, 95-109. | |
| 739 | van Klink, R., Bowler, D. E., Gongalsky, K. B., Swengel, A. B., Gentile, A., & Chase, J. M., (2020). Meta-analysis reveals declines in terrestrial but increases in freshwater insect abundances. *Science*, 368(6489), 417–420. | |
| 740 | Van Rensburg, C. J., King, P. H., & van As, J. G., (2016). Furcocercous cercariae shed by the freshwater snails Pila occidentalis (Mousson, 1887) and *Biomphalaria pfeifferi* (Krauss, 1848) in the Okavango Delta, Botswana. *African Journal of Aquatic Science,* 41(2), 193-203. | |
| 741 | Vassiliades, G., (1978). Snail resistance to dryness - case of Limnaea-natalensis, an intermediate host of Fasciola-gigantica in Senegal. *Revue d'elevage et de Medecine Veterinaire des Pays Tropicaux,* 31(1), 57-62. | |
| 742 | Vera, C., Bremond, P., & Sellin, B., (1995). Seasonal fluctuations in population-densities of *Bulinus-senegalensis* and *b-truncatus* (Planorbidae) in temporary pools in a focus of Schistosoma-haematobium in Niger - implications for control. *Journal of Molluscan Studies,* 61, 79-88. | |
| 743 | Vercruysse, J., Southgate, V.R., & Mungomba, I.M., (1994). Studies on transmission and schistosome interactions in Senegal, Mali and Zambia. *Tropical and geographical medicine,* 46(4), 220-226. | |
| 744 | Vicary, M.P.V., (1981). Decouvert recente d'un foyer de bilharziose a *Schistosoma mansoni* dans une region d'altitude au Rwanda. *Medecine Tropicale,* 41(6), 653-656. | |
| 745 | Visser, L.G.,Polderman, A.M., & Stuiver, P.C., (1995). Outbreak of *Schistosomiasis* among Travelers Returning from Mali, West Africa. *Clinical Infectious Diseases,* 20(2), 280-285. | |
| 746 | Vörösmarty, C. J., McIntyre, P. B., Gessner, M. O., Dudgeon, D., Prusevich, A., Green, P., Glidden, S., Bunn, S. E., Sullivan, C. A., Liermann, C. R., & Davies, P. M., (2010). Global threats to human water security and river biodiversity. *Nature*, 467, 555-561. | |
| 747 | Vrijenhoek, R. C., & Graven, M. A., (1992). Population-genetics of Egyptian *Biomphalaria-alexandrina* (Gastropoda, Planorbidae). *Journal of Heredity,* 83(4), 255-261. | |
| 748 | Wallace, H. A., Clyde R. W., Aida R., & Paul, L. P., (1975). The Moroccan Food Snail, Helix aspersa, as a Source of Salmonella. *Applied Microbiology,* 29(3), 328-330. | |
| 749 | Walz Y., Wegmann, M., Dech, S., Vounatsou, P., Poda, J. N., N'Goran, E. K., Utzinger, J., & Raso, G., (2015)a. Modeling and Validation of Environmental Suitability for Schistosomiasis Transmission Using Remote Sensing. *PLoS Neglected Tropical Diseases,* 9(11), 1-22. | |
| 750 | Walz, Y., Wegmann, M., Leutner, B., Dech, S., Vounatsou, P., N'Goran, E. K. N., Raso, G., & Utzinger, J., (2015)b. Use of an ecologically relevant modelling approach to improve remote sensing-based *Schistosomiasis* risk profiling. *Geospatial Health,* 10(2), 271-279. | |
| 751 | Wanas, M. Q. A., Abou-Senna, F.M., & Al-Shareef, A. E. D. M. F., (1993). Studies on larval digenetic trematodes of Xiphidiocercariae from some Egyptian fresh water snails. *Journal of the Egyptian Society of Parasitology,* 23(3), 829-850. | |
| 752 | Watson, J. M., (1954). Distribution, importance and prevention of urinary bilharziasis in the valley of the Tigris and Euphrates rivers. *Journal Medical Libanais,* 5(1), 13-29. | |
| 753 | Webbe, G., & Msangi, A. S., (1958). Observations on three species of *Bulinus* on the east coast of Africa. *Annals of Tropical Medicine and Parasitology,* 52(3), 302-314. | |
| 754 | Webster, B. L., Webster, J. P., Gouvras, A. N., Garba, A., Lamine, M. S., Diaw, O. T., Seye, M. M., Tchuenté, L. A. T., Simoonga, C., Mubila, L., Mwanga, J. R., Lwambo, N. J. S., Kabatereine, N. B., Lange, C. N., Kariuki, C., Mkoji, G. M., Rollinson, D., & Stothard, J. R., (2013). DNA ‘barcoding’ of *Schistosoma mansoni* across sub-Saharan Africa supports substantial within locality diversity and geographical separation of genotypes. *Acta Tropica,* 128(2), 250-260. | |
| 755 | Weigand, A. M., & Plath, M., (2014). Prey preferences in captivity of the freshwater crab Potamonautes lirrangensis from Lake Malawi with special emphasis on molluscivory. *Hydrobiologia,* 739(1), 145-153. | |
| 756 | Wembo, Ndeo, O., Clewing, C., Stelbrink, B., & Albrecht, C., (2020). Lake Tanganyika endemic gastropods also occur in the Lukuga River. *Journal of Great Lakes Research* 46, 1162-1167. | |
| 757 | Wembo Ndeo, O., Hauffe, T., Delicado, D., Kankonda Busanga, A., & Albrecht, C., (2017). Mollusk communities of the central Congo River shaped by combined effects of barriers, environmental gradients, and species dispersal. *Journal of Limnology,* 76(3), 503-513. | |
| 758 | Wembo Ndeo, O., Kangela, V., Mathe Saliki, F., Yende Grevisse, R., & Kankonda, A., (2018). Endemic gastropods of the Central Congo River: A conservation assessment. *International Journal of Fisheries and Aquatic Studies,* 6(4), 277-281. | |
| 759 | Wen, S. T., & Chu, K. Y., (1984). Preliminary Schistosomiasis survey in the lower Volta river below Akosombo dam, Ghana. *Annals of tropical medicine and parasitology,* 78(2), 129-133. | |
| 760 | West, K., & Michel, E., (2000). The dynamics of endemic diversification: molecular phylogeny suggests an explosive origin of the thiarid gastropods of Lake Tanganyika. *Advances in Ecological Research,* 31, 331-354 | |
| 761 | Wibauxcharlois, M., Yelnik, A., & Ripert, C., (1982). An epidemiological-study of urinary Bilharziasis in the rice fields of Yagoua (North Cameroon) .2. Distribution and Ecology of the Intermediate Hosts. *Bulletin de la Societe de Pathologie Exotique,* 75(1), 72-93. | |
| 762 | Wight, C. A., (1963). The freshwater gastropod Mollusca of Angola. *Bulletin of the British Museum (Natural History), Zoology,* 10, 449-528. | |
| 763 | Wijers, D. J., & Munanga, P. N., (1971). *Schistosomiasis* on Mfangano Island (South Nyanza, Kenya). *East African Medical Journal,* 48(3), 135-40. | |
| 764 | Wilken, G. B., & Appleton, C. C., (1991). Avoidance responses of some indigenous and exotic fresh-water Pulmonate snails to leech predation in South Africa. *South African Journal of Zoology,* 26(1), 6-10. | |
| 765 | Wilkinson, S., Emery, A. M., Khamis, I. S., Mgeni, A. F., Stothard, J. R., & Rollinson, D., (2007). Spatial and temporal population genetic survey of Bulinus globosus from Zanzibar: an intermediate host of *Schistosoma* *haematobium. Journal of Zoology,* 272(3), 329-339. | |
| 766 | Williams, S. N., & Hunter, P. J., (1967). Distribution of Biomphalaria Species in Sudan. *Nature* 215, 1408. | |
| 767 | Williams, S. N., & Hunter, P. J., (1968). The distribution of *Bulinus* and *Biomphalaria* in Khartoum and Blue Nile Provinces, Sudan. *Bulletin of the World Health Organization*, 39(6), 949–954. | |
| 768 | Wilson, A. B., Glaubrecht, M. & Meyer, A., (2004). Ancient lakes as evolutionary reservoirs: evidence from the thalassoid gastropods of Lake Tanganyika. *Proceedings of the Royal Society of London*, B, 271, 529-536. | |
| 769 | Woldemichael, T. T., (1990). Susceptibility of *Biomphalaria-pfeifferi* from amibara irrigation scheme to *Schistosoma-mansoni* parasites from Adua and Gursume, Ethiopia. *Ethiopian medical journal,* 28(3), 145-147. | |
| 770 | Woodward, S. P. (1859). On some new freshwater shells from Central Africa. *Proceedings of the Zoological Society of London*, 1859, 348-349. | |
| 771 | Woolhouse, M. E. J., & Chandiwana, S. K., (1990). Population Biology of the Freshwater Snail *Bulinus globosus* in the Zimbabwe Highveld. *Journal of Applied Ecology,* 27(1), 41-59. | |
| 772 | Woolhouse, M. E. J., (1992). Population Biology of the Freshwater Snail *Biomphalaria pfeifferi* in the Zimbabwe Highveld. *Journal of Applied Ecology,* 29(3), 687-694. | |
| 773 | World Health Organization (WHO), (1958). *Bilharziasis. Bulletin of the World Health Organization,* 18(5-6). | |
| 774 | [World Health Organization (WHO), (2012). *Elimination of* *Schistosomiasis*. Sixty-fifth World Health Assembly, WHA65. 21, Agenda item 13.11, 26 May 2012. Available at: http://apps.who.int/gb/ebwha/pdf_files/WHA65/A65_R21-en.pdf (Accessed January 2024).](http://apps.who.int/gb/ebwha/pdf_files/WHA65/A65_R21-en.pdf) | |
| 775 | [World Health Organization (WHO), (2016). *Schistosomiasis.* Available at: https://www.who.int/features/factfiles/schistosomiasis/en/ (Accessed January 2024).](https://www.who.int/features/factfiles/schistosomiasis/en/) | |
| 776 | Wrable, M., (2017). Exploring the Association Between Remotely Sensed Environmental Parameters and Surveillance Disease Data: *An Application to the Spatiotemporal Modelling of Schistosomiasis in Ghana.* Tufts University. | |
| 777 | Wrable, M., Liss, A., Kulinkina, A., Koch, M., Biritwum, N.K., Ofosu, A., Kosinski, K.C., Gute, D.M., & Naumova, E.N., (2016). Linking satellite remote sensing based environmental predictors to disease: *an application to the spatiotemporal modelling of schistosomiasis in Ghana*. 23rd Congress of the International-Society-for-Photogrammetry-and-Remote-Sensing (ISPRS) 2016 \| XXIII ISPRS Congress, Commission VIII 41 (B8), 215-221. | |
| 778 | Wright, C. A., & Bennett, M. S., (1967). Studies on *Schistosoma haematobium* in the laboratory I. A strain from Durban, Natal, South Africa. *Transactions of the Royal Society of Tropical Medicine and Hygiene* 61(2), 221-227. | |
| 779 | Wright, C. A., (1956). *Bulinus (Pyrgophysa) forskalii* (Ehrenberg) as a Vector of *Schistosoma haematobium*. *Nature,* 177, 43. | |
| 780 | Wright, C. A., (1963). The freshwater gastropod Mollusca of Angola. *Bulletin of the British Museum (Natural History), Zoology,* 10, 449-528. | |
| 781 | Wright, C. A., (1965). The freshwater gastropod molluscs of West Cameroon. *Bulletin of the British Museum (Natural History), Zoology,* 13, 75-98. | |
| 782 | Wright, C. A., (1980). Biology and systematics of the molluscan hosts of *Schistosomiasis* in the Mediterranean Basin in perspective. *Parassitologia* (Rome), 22(3), 257-262. | |
| 783 | Wright, C. A., Klein, J., & Eccles, D. H., (1967). Endemic species of *Bulinus* (Mollusca - Planorbidae) in Lake Malawi (=Lake Nyasa). *Journal of Zoology,* 151:199 | |
| 784 | Yacoubi, B., Zekhnini, A, Rondelaud, D., Vignoles, P., Dreyfuss, G., Cabaret, J., & Moukrim, A., (2007). Habitats of Bulinus truncatus and Planorbarius metidjensis, the intermediate hosts of urinary schistosomosis, under a semiarid or an arid climate. *Parasitology Research,* 101, 311–316. | |
| 785 | Yapi, Y., Ngoran, K. E., & Bellec, C., (1994). Population-dynamics of Indoplanorbis-exustus (Deshayes, 1834) Gastropoda, Planorbidae), an exotic fresh-water snail recently discovered at Yamoussoukro (ivory-coast). *Journal of Molluscan Studies,* 60, 83-87. | |
| 786 | Yigezu, G., Mandefro, B., Mengesha, Y., Yewhalaw, D., Beyene, A., Ahmednur, M., Abdie, Y., Kloos, H., & Mereta, T. S., (2018). Habitat suitability modelling for predicting potential habitats of freshwater snail intermediate hosts in Omo-Gibe river basin, Southwest Ethiopia. *Ecological Informatics,* 45, 70-80. | |
| 787 | Yonge, C. M., (1938). The prosobranchs of Lake Tanganyika. *Nature*, 142, 464–466. | |
| 788 | Younes, A., El-Sherief, H., Gawish, F., & Mahmoud, M., (2017). Biological control of snail hosts transmitting *Schistosomiasis* by the water bug, Sphaerodema urinator. *Parasitology Research,* 116, 1257–1264. | |
| 789 | Yousif, F., Kamel, G., & Mohamed, S., (1993). Ecology of *Biomphalaria alexandrina* the snail vector of *Schistosoma mansoni* in Egypt. J*ournal of the Egyptian Society of Parasitology,* 23(1), 29-42. | |
| 790 | Youssef, M. M., Mansour, N. S., & Boulos, L. M., (1987). Studies on some developmental stages in the life cycle of *Pygidiopsis genata* Looss 1907 (Trematoda: Heterophidae) from Egypt. *Journal of the Egyptian Society of Parasitology,*17(2), 463-474. | |
| 791 | Zein-Eddine, R., Djuikwo-Teukeng, F. F., Dar, Y., Dreyfuss, G., & Van den Broeck, F., (2017). Population genetics of the *Schistosoma* snail host *Bulinus truncatus* in Egypt. *Acta Tropica,* 172, 36-43. | |
